# Parallel Synthesis of Peptide-Like Macrocycles Containing Imidazole-4,5-dicarboxylic Acid

**DOI:** 10.3390/molecules17055346

**Published:** 2012-05-08

**Authors:** Zhigang Xu, Kraig A. Wheeler, Paul W. Baures

**Affiliations:** 1 Department of Pharmacology and Toxicology, The University of Arizona, Tucson, AZ 85724, USA; E-Mail: zxu@pharmacy.arizona.edu; 2 Department of Chemistry, Eastern Illinois University, Charlestown, IL 61920, USA; E-Mail: kawheeler@eiu.edu; 3 Department of Chemistry, Keene State College, Keene, NH 03435, USA

**Keywords:** macrocycle, scaffold, library synthesis, imidazole-4,5-dicarboxylic acid

## Abstract

We prepared a series of peptide-like 14-membered macrocycles containing an imidazole-4,5-dicarboxylic acid scaffold by using known coupling reagents and protecting group strategies. Yields of the purified macrocycles were poor on average, yet seemingly independent of amino acid substitution or stereochemistry. The macrocycles retain some level of conformational variability as observed by both molecular modeling and X-ray crystallography. These macrocycles represent a new class of structures for further development and for future application in high-throughput screening against a variety of biological targets.

## 1. Introduction

Natural products contain a wide variety of conformationally-constrained, macrocyclic, peptide-based rings that present both backbone and side chain pharmacophoric groups for interacting with biological targets [1]. Such biologically-active compounds are generally used by plants and animals in chemical warfare in order to gain an advantage against competitors or assaults [2]. Peptide based macrocycles from marine sources continue to be investigated for their biological activity [2,3], as are similar compounds from plants [4].

Medicinal chemists have long used macrocyclization as a tool in drug discovery. A classic illustration of this technique was the discovery of potent cyclic peptide somatostatin mimics three decades ago [5,6]. The use of cyclization has since developed into a general paradigm in peptidomimetic drug design that has been widely used in the discovery of biologically-active compounds for studying the cell [7]. Indeed, there are both natural product and synthetic macrocyclic peptides with known anticancer use or potential [8], thereby illustrating their functional similarity.

Oxazoles and thiazoles, as well as oxazoline and thiazoline rings, are commonly found in natural product macrocyclic peptides [9,10]. Several research groups have reported on the synthesis of macrocyclic peptides containing heterocyclic amino acids [11,12,13,14,15,16]. Other heterocyclic amino acids that have been incorporated into cyclic peptides include pyridine [17] and 1,4-triazole [18].

In addition to the potency and selectivity gained by macrocyclization of peptides, cyclic peptides also resist proteolytic degradation and other metabolism, plus they are more cell permeable than the corresponding linear peptides [19,20,21,22,23,24,25,26,27]. These are important considerations for compounds to be valuable as chemical tools for studying the cell.

We now report the synthesis and characterization of a peptide-like macrocyclic library that contains imidazole-4,5-dicarboxylic acid, thereby yielding a unique compound class out of inspiration from macrocyclic natural products and known paradigms in drug discovery. The final compounds are 14-membered macrocycles with variations in stereochemistry and amino acid side chains. The side chain variations are limited in comparison with those possible, but nonetheless illustrate the ability to form the macrocycles regardless of stereochemistry or larger side chains. Indeed, macrocycles are underrepresented in screening libraries due in part to their synthetic challenge [28,29], and this work is an important first step in the development of imidazole-4,5-dicarboxylic acid-based macrocycles for screening efforts. The value of the imidazole-4,5-dicarboxylic acid scaffold in drug discovery has been previously reported [30], as have other libraries for screening efforts that were built around this scaffold [31,32,33].

The library members obey Lipinski’s rule-of-five (RO5) for drug-like compounds [34,35,36], and also have limited conformational flexibility that is favorable for pharmacokinetic properties [37]. Regardless, tool-like compounds that are outside the RO5 in molecular weight, hydrogen bond donors or acceptors, and Log P values are still valuable for use in the early screening and chemical genomic research [38].

It is important to note that these macrocycles represent a new compound class and are different from the small compound libraries previously reported that used the imidazole-4,5-dicarboxylic acid scaffold [31,32,33]. Their features mimic those of known bioactive natural products, and we anticipate that such compounds would have reasonable pharmacokinetic properties. Thus, we reason the macrocycles will be valuable for screening efforts in a variety of bioassays and these results will be reported in time.

## 2. Results and Discussion

### 2.1. Synthesis

**Chart 1:**
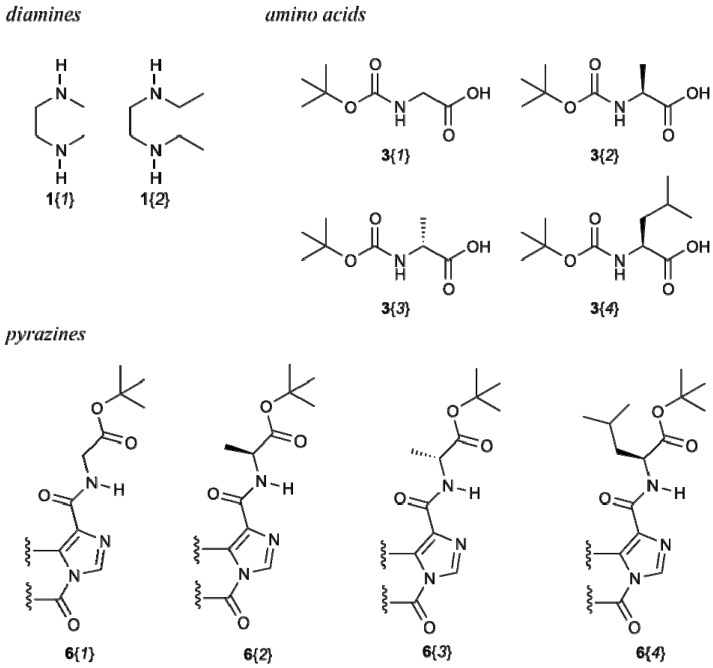
Building blocks for the macrocycles.

The building blocks for the synthesis of the macrocycles are shown in Chart 1.

**Scheme 1:**
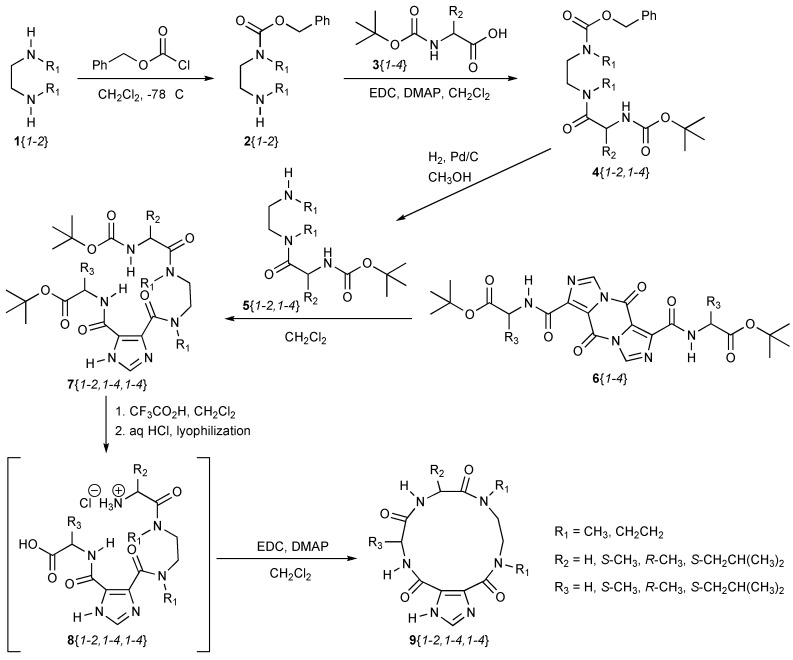
Synthetic strategy for the macrocycle synthesis.

The synthesis of the macrocycles begins with commercially available *N*,*N*′-dialkylalkanediamines, **1**{*1-2*}, as shown in Scheme 1. These are monoprotected with benzyloxycarbonyl chloride by using an excess of the diamine to yield **2**{*1-2*} following workup and purification as similarly reported [39]. However, this method worked poorly for **1**{*1*} and resulted in significant amounts of diprotected diamine. An alternative synthesis was therefore used that employed *N*-Boc protection first [40] followed by benzyloxycarbonyl chloride protection to give the orthogonally protected diamine. Subsequent removal of the *N*-Boc protecting group with CF_3_CO_2_H in CH_2_Cl_2_ [31] yielded **2**{*1*}. These intermediates were coupled to the Boc-amino acid building blocks, **3**{*1-4*}, with EDC and DMAP in CH_2_Cl_2_ [29], providing chemset **4**. Hydrogenolysis of chemset **4** in MeOH [31] yielded chemset **5** following workup. The pyrazine building blocks, **6**{*1-4*}, were prepared as previously described [33], and reacted with chemset **5** in CH_2_Cl_2_ to yield acyclic chemset **7** following purification by column chromatography. The stoichiometry of the reaction between chemset **5** and chemset **6** is 2:1, respectively, and yields 2 equivalents of chemset **7**. The *tert*-butyl ester and *N*-Boc protecting groups were removed by using CF_3_CO_2_H in CH_2_Cl_2_ [31]. The trifluoroacetate was exchanged for a chloride by adding dilute HCl, freezing the solution, and lyophilizing to give chemset **8**. Macrocyclic ring closure to chemset **9** was affected by dissolving chemset **8** in CH_2_Cl_2_ and adding EDC with DMAP [31]. The final macrocycles were also purified by column chromatography and characterized by both LC-MS and ^1^H-NMR spectroscopy.

No extensive effort was made to optimize the synthesis of these macrocycles, and the yields to purified macrocycles are poor across the series, averaging 12 ± 11% yield, with the best yield being 37% for both **9**{*1,1,4*} and **9**{*1,4,3*}. A single reaction trying to form the macrocycle **9**{*1,1,2*} with HBTU and Et_3_N in DMF was attempted [41], but no product was observed by LC-MS analysis. Indeed, no final product was identified for nine attempted macrocyclization reactions, and the starting material was not isolated for an additional four macrocycles, leaving 19 of 32 planned macrocycles being synthesized in the study. The yields to the chromatographically-pure macrocycles are given in Table 1. Importantly, we did not observe a clear trend relating stereochemistry or size of the side chain in preventing the formation of the macrocycle, so we reason it would be possible to expand this library in the future to include greater diversity in these areas. This result is nonetheless an important and critical first step in the goal of targeting screening libraries of these and closely related compounds, especially given the general challenge in the synthesis of macrocycles [28,29].

The final macrocycles all contain three hydrogen bond donors and five hydrogen bond acceptors, thereby conforming to the principles of fewer than five hydrogen bond donors and 10 hydrogen bond acceptors as outlined in Lipinski’s rule-of-five (RO5) for drug-like compounds [34,35,36]. The final macrocycles molecular weights and cLogP values are provided in Table 2. The molecular weights are all less than 500 g/mol, with cLogP values ranging from −1.70 to 3.31, clearly below the desired limit of 5.

### 2.2. Characterization

X-ray crystallography was done on **9**{*2*,*4*,*1*}, **9**{*2*,*4*,*3*}, and **9**{*2*,*1*,*3*} in order to gain insights on the conformation of the macrocyclic ring system and select crystal data is provided in Table 3. Interestingly, four different conformers were observed in the asymmetric unit for **9**{*2*,*4*,*1*} and these are labeled conformer A, conformer B, conformer C, and conformer D in the crystal structure. There are also four conformers (labeled conformers A–D) in the X-ray crystal structure of **9**{*2*,*4*,*3*} and two conformers (labeled conformers A and B) in the X-ray crystal structure of **9**{*2*,*1*,*3*}.

A labeling diagram for the macrocycles, averaged torsion angles with deviations for the four conformers A–D of both **9{***2*,*4*,*1***}** and **9{***2*,*4*,*3***}**, the averaged torsion angles with deviations for the two conformers A and B in **9{***2*,*1*,*3***}**, an illustration of conformer A in **9{***2*,*4*,*1***}** shown in a ball-and-stick representation, as well as the comparable torsion angles observed in a Spartan’06 [42,43] energy-minimized structure of **9{***2*,*4*,*1***}** are all given in Table 4.

The Ψ torsion angle (N2-C3-C4-N5; −60.3 ± 13.8°) of the glycine residue in conformers A–D of **9**{*2*,*4*,*1*} is a principal site of observed variability between the conformers found in the crystal structure, as evidenced by the value of the deviation in this angle compared to others in the macrocycle. This is not particularly surprising as glycine is quite flexible relative to the other natural amino acids. Yet, it was surprising to find considerable variability in both the ϕ (C4-N5-C6-C7; −108.4 ± 12.5°) and Ψ (N5-C6-C7-N8; 106.1 ± 7.6°) torsion angles of the leucine, along with striking similarity in the torsion angles of the *N*,*N*′-diethylethylenediamine linker. We would have expected the diamine torsion angles to be the observed site of differences more so than the amino acid residues. In hindsight, we reason this has some relation to the strong intramolecular 7-membered hydrogen bond [44,45], which is seen in each of the conformers of the asymmetric units across the crystal structures, although crystal packing influences cannot be ruled out. The observed NH^…^O distances of this hydrogen bond across conformers A–D in the X-ray crystal structures of both **9**{*2*,*4*,*1*} and **9{***2*,*4*,*3***}**, as well as for conformers A and B in **9**{*2*,*1*,*3*} are provided in Table 5. The comparable values observed in the energy-minimized structure of **9**{*2*,*4*,*1*} is also given in Table 5.

The ϕ,Ψ torsion angles of the leucine (−109.0 ± 6.8°, 111.1 ± 9.1°) and D-alanine (138.8 ± 6.4°, −66.3 ± 3.0°) in **9**{*2*,*4*,*3*} are also principal sites of variability in the conformers A–D observed in this X-ray crystal structure, as again evidenced by the larger deviations in these angles as compared with other torsion angles. Smaller deviations for the respective ϕ,Ψ torsion angles of the glycine (−95.2 ± 1.7°, 112.0 ± 5.7°) and D-alanine (134.1 ± 4.0°, −77.4 ± 0.3°) residues are observed in the two conformers (A and B) of **9**{*2*,*1*,*3*}, although the greater of these deviations are still significant when compared the other torsion angles of the macrocycle.

**Figure 1:**
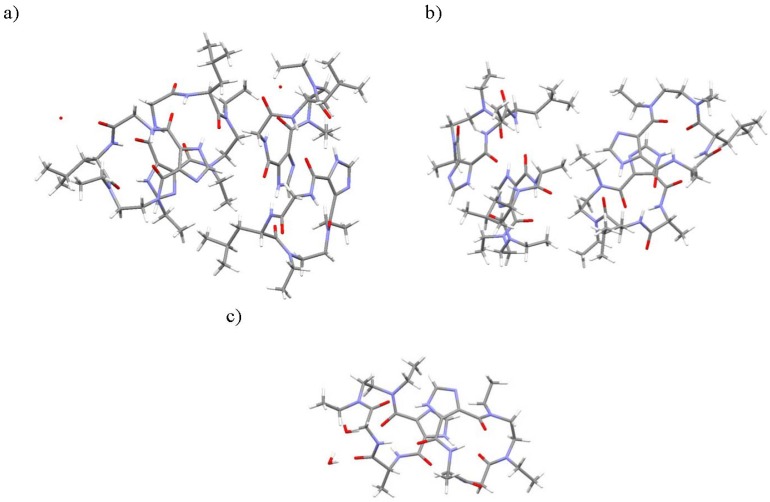
Asymmetric units in the crystal structures of (**a**) **9**{*2*,*4*,*1*}, (**b**) **9**{*2*,*4*,*3*}, and (**c**) **9**{*2*,*1*,*3*}.

As indicated, the intramolecular hydrogen bond is observed in all of the crystal structures, just as previously noted across a wide variety of imidazole-4,5-dicarboxylic acid derivatives [44,45]. The amino acid from chemset **6**{*1-4*} provides the donor NH (N2) in this intramolecular hydrogen bond, with the acceptor (C12=O) coming from the scaffold itself. The macrocycles then form intermolecular dimers in the solid-state, with the imidazole NH and other amide carbonyl from the scaffold (C1=O) forming NH^…^O hydrogen bonds to C7=O and N5-H, respectively, from the amino acid chemset **3**{*1-4*} in the synthetic scheme. These interactions can be observed in the asymmetric units from the X-ray crystal structures as illustrated in Figure 1.

Molecular modeling with Spartan’06 was done on **9**{*2*,*4*,*1*} in order to compare the torsion angles observed in the energy-minimized structure with the averaged torsion angles from the four conformers (A–D) in the X-ray crystal structure (Table 4). Interestingly, a majority of the torsion angles in the energy-minimized model deviate from the observed ranges seen across conformers A–D of **9**{*2*,*4*,*1*}, and only two torsion angles connecting the intramolecular hydrogen bond acceptor (C12=O) with the imidazole ring fall into the observed range. Yet, the parameters for the intramolecular hydrogen bond in the energy-minimized structure do not compare with the ranges of the four conformers (A–D) in the crystal structure (Table 5). While the source of these differences is not clear at this time, the variability in the torsion angles is considered advantageous in the future screening applications of the macrocycle libraries. The thinking is that the macrocycle will provide overall preorganization of the scaffold, but retain enough torsion angle variability about the amino acid residues to help optimize the binding interactions of side chain pharmacophores at targets.

## 3. Experimental

### 3.1. General

All apparatus was oven-dried and cooled in a desiccator. Reagents were purchased from commercial suppliers and used without further purification except reagent grade CH_2_Cl_2_ that was distilled from CaH_2_ before use. Thin layer chromatography (TLC) was performed on 250 µm glass-backed silica gel plates and visualized using UV. Column chromatography was performed on silica gel (Merck, grade 9385, 230–400 mesh, 60 Å). The columns were prepared in plastic 20 or 30 mL syringe bodies and filled to approximately 2/3 of their capacity with silica gel.

### 3.2. LC-MS Analysis

Characterization of the purity and identity of the library members and intermediates was carried out by liquid chromatography-mass spectrometry (LC-MS) using a Varian 500-MS LC Ion Trap mass spectrometer. Solutions of the compounds were prepared at an approximate concentration of 1 mg/mL by first adding <10% by volume of CH_2_Cl_2_ to dissolve the sample and then diluting to the final volume with HPLC grade methanol. Five microliters of the sample was injected onto a Polaris 5 mm C18-A (50 × 2.0 mm) HPLC column and eluted with a gradient of CH_3_CN/H_2_O containing 0.1% CH_3_CO_2_H at a 0.2 mL/min flow rate. Compounds were detected at 214 nm. The gradient was as follows: 0 min., 4:6 CH_3_CN/H_2_O → 1 min., 4:6 CH_3_CN/H_2_O → 6 min., 9:1 CH_3_CN/H_2_O → 8 min., 9:1 CH_3_CN/H_2_O → 9 min., 4:6 CH_3_CN/H_2_O → 10 min., 4:6 CH_3_CN/H_2_O. The mass spectrum was recorded for the entire elution time by using ESI detection from 50–800 (*m*/*z*) with the following parameters: capillary voltage at 60.0, RF loading at 100%, drying gas at 250 °C, spray chamber at 50 °C, nebulizer gas at 50.0 psi, drying gas at 25 psi, and damping gas at 0.8 mL/min.

### 3.3. NMR Spectroscopy

^1^H-NMR and ^13^C-NMR spectra were recorded for the intermediates at 400 MHz and 100.5 MHz, respectively, in CDCl_3_ with CHCl_3_ as the internal reference for ^1^H (δ 7.24) and CDCl_3_ as the internal reference for ^13^C (δ 77.00) or DMSO-*d*_6_ with DMSO as the internal reference for ^1^H (δ 2.49) and DMSO-*d*_6_ as the internal reference for ^13^C (δ 39.50). The ^1^H-NMR spectra of the final macrocycles were determined at 270 MHz in MeOH-*d*_4_ with MeOH as the internal reference for ^1^H (δ 3.34). The spectra for the final macrocycles are provided in the ESI. Coupling constants are expressed in Hertz (Hz). Data are reported in this sequence: (1) chemical shifts (ppm); (2) spin multiplicities given as, s (singlet), bs (broad singlet), d (doublet), t (triplet), q (quartet), quintet, sextet and m (multiplet); (3) coupling constant *J* (Hz); and (4) integration.

### 3.4. X-ray Crystallography

Samples for X-ray diffraction were obtained from the slow evaporation of a wet methanolic solution. Data using CuKα radiation were collected with a Bruker APEX II CCD and integrated with SAINT and XPREP in order to provide the observable reflections [*F*o > 4sig(*F*o)]. Crystal stability was monitored by measuring three standard reflections after every 97 reflections with no significant decay in observed intensities. A*θ*-2*θ* scanning technique was used for peak collection with Lorenz and polarization corrections applied. Hydrogen atom positions were located from difference Fourier maps, and a riding model with fixed thermal parameters [*uij* = 1.2*Uij*(eq)] for the atom to which they are bonded] was used for subsequent refinements. The SHELXL-93 package [46] was used for data reduction, structure solution, and refinement.

CCDC 874142-874144 contain the supplementary crystallographic data for this paper. These data can be obtained free of charge via www.ccdc.cam.ac.uk/conts/retrieving.html (or from the CCDC, 12 Union Road, Cambridge CB2 1EZ, UK; fax: +44 1223 336033; e-mail: deposit@ccdc.cam.ac.uk).

### 3.5. Molecular Modeling

Spartan’06 [42,43] was used to calculate a low-energy conformation for **9**{*2*,*4*,*1*}. A 100-membered conformer library was generated and the 10 lowest energy conformers were minimized with PM3 semi-empirical methods. The lowest energy conformer from the molecular modeling was then compared with the four conformers (A–D) observed in the solid-state by X-ray crystallography.

### 3.6. Synthetic Procedures

Procedures are provided for the protection of the *N*,*N*′-dialkylalkanediamines, along with general methods for the remaining intermediates and final macrocycles. A specific example for each intermediate and for one macrocycle is also provided here. The remaining synthetic details and analytical data are provided in the ESI.

**2**{*1*}. Alternative procedure: To an ice-cooled solution of *N*,*N*′-dimethylethylenediamine (7.4 mL, 68.8 mmol) in dry THF (60 mL) was added a solution of Boc_2_O (5.0 g, 22.9 mmol) in dry THF (40 mL). The reaction mixture was stirred at 0 °C for 30 min. then warmed to room temperature (rt) and stirred another 19.5 h. The reaction mixture was filtered and the filtrate concentrated under vacuum. The residue was dissolved in EtOAc, washed with brine (3 × 50 mL), dried over MgSO_4_, and concentrated under vacuum to yield 3.83 g (85%) of a colorless oil used without further purification. The residue from above was dissolved in 40 mL of dry THF and cooled to 0 °C before concurrently adding Et_3_N (2.84 mL, 20.4 mmol) and benzyl chloroformate (2.86 mL, 20.4 mmol) dropwise over 20 min. The reaction was warmed to rt and stirred another 3 h. The solvent was removed under vacuum. The residue was dissolved in EtOAc (100 mL), washed with brine (3 × 30 mL), dried over MgSO_4_, and concentrated under vacuum to yield 6.35 g (97%) of a colorless oil. TLC R*_f_* (EtOAc/Hex 1:1) = 0.54; ^1^H-NMR (CDCl_3_) δ 7.32 (m, 5H), 5.11 (s, 2H), 3.30 (s, 4H), 2.58 (s, 4H), 1.70 (m, 2H), 1.48 (s, 1H), 1.06 (m, 6H); LC-MS (Method A) 5.25 min; ESI MS *m*/*z* 345 [M+Na]^+^.

To a solution of the above intermediate (6.35 g, 19.7 mmol) in 20 mL of acetone was added 36% aqueous HCl (20 mL) dropwise at rt. The reaction mixture was stirred for 16 h before removing the solvent under vacuum. The residue was dissolved in EtOAc (100 mL), washed with sat. NaHCO_3_, brine, dried over MgSO_4_, and concentrated under vacuum to yield 1.20 g (27%) of a pale yellow oil. LC-MS (Method A) 1.19 min; ESI MS *m*/*z* 223 [M+H]^+^.

**2**{*2*}. To a dry roundbottom flask was added 40 mL of CH_2_Cl_2_ and *N*,*N*′-diethylethylenediamine (10.1 mL, 70.4 mmol). To this stirred solution at −78 °C was added a solution of benzyl chloroformate (1.0 mL, 7.04 mmol) in 40 mL of CH_2_Cl_2_ over 1 h. The reaction was stirred for 1.5 h at −78 °C before warming to rt and stirring for another 18 h. The reaction was filtered and the filtrate washed with brine (3 × 50 mL), dried over MgSO_4_, and concentrated under vacuum. The product was purified by column chromatography using a gradient of EtOAc/MeOH (19:1 → 3:2), yielding 1.40 g (80%) of a brown oil. TLC R*_f_* (EtOAc) = 0.05; ^1^H-NMR (CDCl_3_) δ 7.27–7.33 (m, 5H), 5.11 (s, 2H), 3.32 (m, 4H), 2.59–2.76 (m, 4H), 1.00–1.12 (m, 6H); LC-MS (Method A) 1.45 min; ESI MS *m*/*z* 251 [M+H]^+^.

**4**{*1-2,1-4*}. General Method. To a solution of the *N*,*N*′-dialkylalkanediamine in CH_2_Cl_2_ was added the Boc-amino acid, EDC^.^HCl, and DMAP at rt. The reaction was stirred for a period of time before removing the solvent under vacuum. The product was purified by column chromatography on silica gel.

**4**{*1,1*}. Example. As described using **2**{*1*} (2.00 g, 9.01 mmol) in 100 mL of CH_2_Cl_2_ with **3**{*1*} (1.58 g, 9.01 mmol), EDC^.^HCl (3.79 g, 19.82 mmol), and DMAP (0.22 g, 1.80 mmol) at rt. The reaction was stirred for 16 h and the product was purified by column chromatography on silica gel using EtOAc/hexanes (1:3 → 1:0), as the gradient, yielding 2.94 g (86%) of a colorless oil. TLC R*_f_* (EtOAc/Hex 1:1) = 0.37; ^1^H-NMR (CDCl_3_) δ 7.26–7.33 (m, 5H), 5.38 (s, 1H), 5.08 (d, 2H), 3.73–3.81 (m, 2H), 3.41–3.54 (m, 4H), 2.77–2.97 (m, 6H), 1.43 (s, 9H); LC-MS (Method A) 3.49 min; ESI MS *m*/*z* 380 [M+H]^+^.

**5**{*1-2,1-4*}. General Method. A suspension of one intermediate, **4**{*1-4,1-4*}, and 5% Pd/C in CH_3_OH was stirred at rt under 1 atm of H_2_. The reaction was stirred until complete, filtered to remove the Pd/C, and concentrated under vacuum. The product was used without further purification.

**5**{*1,1*}. Example. As described using **4**{*1,1*} (2.90 g, 7.65 mmol) and 5% Pd/C (0.40 g) in 120 mL of CH_3_OH at rt. The reaction was stirred for 11 h and workup yielded 1.80 g (96%) of a colorless oil. ^1^H-NMR (CDCl_3_) δ 5.49 (s, 1H), 3.92 (q, *J* = 8.0 Hz, 2H), 3.49 (t, *J* = 6.4 Hz, 1H), 3.30 (t, *J* = 6.4 Hz, 1H), 2.94 (d, *J* = 2.8 Hz, 3H), 2.72 (m, 2H), 2.41 (d, *J* = 3.6 Hz, 3H), 1.42 (s, 9H), 1.28 (s, 1H); LC-MS (Method A) 1.20 min; ESI MS *m*/*z* 246 [M+H]^+^.

**6{***1*-*4***}**. Synthesis was done as previously described [33]. Briefly, to a dry round-bottom flask under argon was added dry CH_2_Cl_2_. To this stirred solvent at −78 °C were added, in order, the pyrazine diacid chloride (1 equiv.), the amino acid ester hydrochloride salt (2 equiv.), and *N,N*-diethylaniline (4 equiv.). The resulting solution was held at −78 °C for 30 min before stirring at room temperature. The reaction was washed against water (3×) and the organic fraction was dried over MgSO_4_, filtered and concentrated. The residue was suspended in boiling EtOAc, stirred for 15 min and then cooled at 0 °C. The solid product **6{***1-4*} was collected by vacuum filtration and washed with EtOAc. The final product was characterized by ^1^H-NMR spectroscopy.

**6{***1***}**. Example. ^1^H-NMR (CDCl_3_) δ 8.64 (s, 2H), 8.59 (t, *J* = 4.8 Hz, 2H), 4.17 (d, *J* = 4.8 Hz, 2H), 1.49 (s, 18H).

**7**{*1-2,1-4,1-4*}. General Method. To a suspension of one amino acid ester substituted pyrazine, **6**{*1-4*} in CH_2_Cl_2_ was added one intermediate, **5**{*1-2,1-4*} at rt. The reaction was stirred until complete, the solvent removed under vacuum, and the product purified by column chromatography on silica gel.

**7**{*1,1,1*}. Example. As described using **6**{*1*} (0.20 g, 0.40 mmol) and **5**{*1,1*} (0.25 g, 1.00 mmol) in 20 mL of CH_2_Cl_2_ at rt. The reaction was stirred for 19.5 h. Column chromatography using a gradient of EtOAc/Hex (1:1 → 1:0) followed by EtOAc/MeOH (1:19 → 4:1) yielded 0.25 g (63%) of a white solid. TLC R*_f_* (EtOAc/MeOH 9:1) = 0.25; ^1^H-NMR (CDCl_3_) δ 12.26 (m, 1H), 10.50–10.93 (m, 1H), 7.58 (d, *J* = 5.6 Hz, 1H), 5.56 (t, *J* = 11.2 Hz, 1H), 3.64–4.19 (m, 8H), 2.85–3.42 (m, 6H), 1.47 (s, 9H), 1.39 (s, 9H); ^13^C-NMR (CDCl_3_) δ 169.0, 168.9, 168.4, 168.3, 168.2, 168.1, 165.9, 165.8, 165.4, 159.7, 159.5, 158.6, 155.8, 136.5, 135.4, 134.8, 134.6, 134.5, 134.4, 133.8, 130.7, 129.7, 129.3, 129.2, 129.0, 81.9, 79.5, 79.3, 52.9, 49.5, 48.6, 47.9, 47.2, 46.7, 46.6, 45.9, 45.4, 42.6, 42.4, 42.3, 41.9, 41.6, 39.0, 38.6, 35.9, 35.6, 35.0, 34.5, 34.2, 34.0, 28.3, 28.0; LC-MS (Method A) 1.67 min; ESI MS *m*/*z* 497 [M+H]^+^.

**8**{*1-2,1-4,1-4*}. General Method. To a solution of one intermediate, **7**{*1-2,1-4,1-4*} in CH_2_Cl_2_ was added trifluoroacetic acid at rt. The reaction was stirred until complete and the solvent removed under vacuum. The residue was taken up in aqueous HCl and lyophilized to dryness, yielding a material that was used without further purification.

**8**{*1,1,1*}. Example. As described using **7**{*1,1,1*} (210 mg, 0.423 mmol), 5 mL of CH_2_Cl_2_ and 1 mL of TFA. The reaction was stirred for 13 h. LC-MS (Method A) 1.14 min; ESI MS *m*/*z* 341 [M+H]^+^.

**9**{*1-2,1-4,1-4*}. General Method. To 20 mL of CH_2_Cl_2_ in a dry roundbottom flask was added dropwise over 1 h a solution of one intermediate, **7**{*1-32*} (1 equiv.) in 20 mL of CH_2_Cl_2_ concurrent with the dropwise addition of a solution containing EDC^.^HCl (2.2 equiv.) and DMAP (0.2 equiv.) in 20 mL of CH_2_Cl_2_. The resulting solution was stirred for a period of time before removing the solvent under vacuum and purifying the macrocycle by column chromatography on silica gel. The chemset numbers for the final products and their associated names are provided in Table 6.

**9**{*1,1,1*}. Example. As described using **8**{*1,1,1*} (72 mg, 0.210 mmol), EDC^.^HCl (89 mg, 0.466 mmol), and DMAP (5 mg, 0.042 mmol). The reaction was stirred for 10.5 h. Column chromatography using a gradient of EtOAc/Hex (1:1 → 1:0) followed by EtOAc/MeOH (1:19 → 3:2) yielded 4.9 mg (7%) of a white solid. TLC R*_f_* (EtOAc/MeOH 1:1) = 0.25; LC-MS (Method A) 1.21 min; ESI MS *m*/*z* 323 [M+H]^+^.

## 4. Conclusions

Macrocyclic peptides are useful tools in the drug discovery, owing in part to the conformational preorganization of their pharmacophores, reduced proteolytic degradation and metabolism, as well as increased bioavailability compared to acyclic peptides. Imidazole-4,5-dicarboxylic acid is a useful scaffold for drug discovery that is readily diversified with amines to yield imidazole-4,5-dicarboxamides. We now illustrate the ability of such derivatives to be further modified into 14-membered peptide-like macrocyclic rings. These final products are natural product-like and the synthesis accepts modifications in stereochemistry and amino acid side chains. The yields to the chromatographically pure final products are poor at this time, but efforts to optimize the macrocyclization and extend the types of substituents presented by the macrocyclic ring will be undertaken. The products may also be valuable in screening collections looking for hits against a wide variety of drug targets, and these efforts too will be reported on in due time.

## Figures and Tables

**Table 1:** Purified yields to the final macrocycles, chemset **9**.

**R_1_ = Me**				
	**R_2_ = H**	**R_2_ = (*S*)-CH_3_**	**R_2_ = (*R*)-CH_3_**	**R_2_ = (*S*)-CH_2_CH(CH_3_)_2_**
**R_3_ = H**	**9**{*1,1,1*}; 7%	**9**{*1,2,1*}; —	**9**{*1,3,1*}; —	**9**{*1,4,1*}; —
**R_3_ = (*S*)-CH_3_**	**9**{*1,1,2*}; 7%	**9**{*1,2,2*}; 32%	**9**{*1,3,2*}; 22%	**9**{*1,4,2*}; 14%
**R_3_ = (*R*)-CH_3_**	**9**{*1,1,3*}; 17%	**9**{*1,2,3*}; —	**9**{*1,3,3*}; —	**9**{*1,4,3*}; 37%
**R_3_ = (*S*)-CH_2_CH(CH_3_)_2_**	**9**{*1,1,4*}; 37%	**9**{*1,2,4*}; 12%	**9**{*1,3,4*}; 11%	**9**{*1,4,4*}; ‡
**R_1_ = Et**				
	**R_2_ = H**	**R_2_ = (*S*)-CH_3_**	**R_2_ = (*R*)-CH_3_**	**R_2_ = (*S*)-CH_2_CH(CH_3_)_2_**
**R_3_ = H**	**9**{*2,1,1*}; —	**9**{*2,2,1*}; —	**9**{*2,3,1*}; 3%	**9**{*2,4,1*}; 8%
**R_3_ = (*S*)-CH_3_**	**9**{*2,1,2*}; 9%	**9**{*2,2,2*}; 7%	**9**{*2,3,2*}; 10%	**9**{*2,4,2*}; 11%
**R_3_ = (*R*)-CH_3_**	**9**{*2,1,3*}; 8%	**9**{*2,2,3*}; 8%	**9**{*2,3,3*}; 9%	**9**{*2,4,3*}; —
**R_3_ = (*S*)-CH_2_CH(CH_3_)_2_**	**9**{*2,1,4*}; ‡	**9**{*2,2,4*}; ‡	**9**{*2,3,4*}; ‡	**9**{*2,4,4*}; —

‡: No starting material was available; —: No product was identified.

**Table 2:** Molecular weights and cLogP values for the macrocycles.

cmpd	MW (g/mol)	cLogP		cmpd	MW (g/mol)	cLogP
**9**{*1,1,1*}	322.32	−1.70		**9**{*2,1,1*}	350.37	−0.64
**9**{*1,1,2*}	336.35	−1.36		**9**{*2,1,2*}	364.40	−0.12
**9**{*1,1,3*}	336.35	−1.36		**9**{*2,1,3*}	364.40	−0.12
**9**{*1,1,4*}	378.43	0.10		**9**{*2,1,4*}	406.40	1.33
**9**{*1,2,1*}	336.35	−1.36		**9**{*2,2,1*}	364.40	−0.12
**9**{*1,2,2*}	350.37	−0.84		**9**{*2,2,2*}	378.43	0.40
**9**{*1,2,3*}	350.37	−0.84		**9**{*2,2,3*}	378.43	0.40
**9**{*1,2,4*}	392.45	0.62		**9**{*2,2,4*}	420.51	1.85
**9**{*1,3,1*}	336.35	−1.36		**9**{*2,3,1*}	364.40	−0.12
**9**{*1,3,2*}	350.37	−0.84		**9**{*2,3,2*}	378.43	0.40
**9**{*1,3,3*}	350.37	−0.84		**9**{*2,3,3*}	378.43	0.40
**9**{*1,3,4*}	392.45	1.85		**9**{*2,3,4*}	420.51	1.85
**9**{*1,4,1*}	378.43	0.10		**9**{*2,4,1*}	406.48	1.33
**9**{*1,4,2*}	392.45	0.62		**9**{*2,4,2*}	420.51	1.85
**9**{*1,4,3*}	434.53	0.62		**9**{*2,4,3*}	420.51	1.85
**9**{*1,4,4*}	434.53	2.08		**9**{*2,4,4*}	462.59	3.31

**Table 3:** Selected crystal data collection and refinement data.

Crystal data	9{*2*,*4*,*1*}	9{*2*,*4*,*3*}	9{*2*,*1*,*3*}
**formula**	C_19_H_30_N_6_O_4_ + 0.16O	C_20_H_32_N_6_O_4_	C_16_H_24_N_6_O_4_ + H_2_O
**weight (g mol^−1^)**	409.03	420.51	382.43
**crystal size (mm)**	0.45 × 0.38 × 0.28	0.45 × 0.13 × 0.07	0.35 × 0.07 × 0.05
**crystal system**	monoclinic	monoclinic	orthorhombic
**space group**	P2_1_	P2_1_	P2_1_2_1_2_1_
***a* (Å)**	13.7441(2)	14.0826(7)	8.6170(1)
***b* (Å)**	14.4322(2)	13.9531(7)	17.9394(3)
***c* (Å)**	21.3948(3)	22.4144(12)	24.4406(4)
**β (°)**	94.192(1)	96.766(3)	-
**cell volume (Å^3^)**	4,232.5(1)	4,373.7(4)	3,778.1(1)
**Z**	8	8	8
**temp (K)**	100(2)	100(2)	100(2)
**S**	1.017	1.093	0.994

**Table 4:**
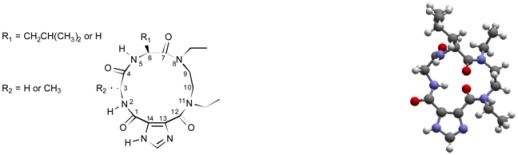
Crystallographic details for **9**{*2*,*4*,*1*}, **9**{*2*,*4*,*3*}, and **9**{*2*,*1*,*3*}.

Compound						
Torsion Angle	9{*2*,*4*,*1*} ‡	9{*2*,*4*,*3*} ‡		9{*2*,*1*,*3*} †	Minimized 9{2,4,1} §	
1-2-3-4	131.2 ± 4.3°	138.8 ± 6.4°	134.1 ±4.0°			**109.6°**
2-3-4-5	−60.3 ± 13.8°	−66.3 ± 3.0°	−77.4 ± 0.3°			**−82.8°**
3-4-5-6	164.2 ± 4.5°	165.5 ± 5.2°	165.0 ± 1.6°			**156.0°**
4-5-6-7	−108.4 ± 12.5°	−109.0 ± 6.8°	−95.2 ± 1.7°			**−93.7°**
5-6-7-8	106.1 ± 7.6°	111.1 ± 9.1°	112.0 ± 5.7°			**129.1°**
6-7-8-9	175.4 ± 3.2°	177.1 ± 2.3°	174.7 ± 1.8°			**−176.5°**
7-8-9-10	102.2 ± 1.7°	100.8 ± 5.4°	98.5 ± 3.9°			**95.5°**
8-9-10-11	−75.6 ± 4.2°	−77.0 ± 4.2°	−71.9 ± 0.9°			**−89.4°**
9-10-11-12	96.3 ± 0.9°	99.4 ± 2.9°	103.6 ± 3.1°			**90.5°**
10-11-12-13	176.1 ± 1.0°	175.4 ± 3.0°	173.3 ± 0.2°			**−175.0°**
11-12-13-14	147.2 ± 3.9°	145.9 ± 3.1°	137.3 ± 1.6°			149.8°
12-13-14-1	−2.2 ± 1.2°	−3.8 ± 1.9°	−1.1 ± 4.1°			−2.6°
13-14-1-2	15.7 ± 4.5°	13.6 ± 4.3°	16.3 ± 0.9°			**39.9°**
14-1-2-3	−167.9 ± 2.1°	−166.9 ± 2.7°	−166.4 ± 0.3°			**−163.5°**

‡ Four conformers were observed in the X-ray crystal structure and the data presented is the average with the observed deviation in these torsion angles; † Two conformers were observed in the X-ray crystal structure and the data presented is the average with the observed deviation in these torsion angles; § The emboldened torsion angles are outside the range observed in conformers A–D for the crystal structure of **9**{*2*,*4*,*1*}; The ball-and-stick illustration is Conformer A from the X-ray crystal structure of **9**{*2*,*4*,*1*}.

**Table 5:** Intramolecular hydrogen bonding details for **9**{*2*,*4*,*1*}, **9**{*2*,*4*,*3*}, and **9**{*2*,*1*,*3*}.

Measurement	Molecule in Asymmetric Unit			
	9{*2*,*4*,*1*}	9{*2*,*4*,*3*}	9{*2*,*1*,*3*}	Minimized 9{*2*,*4*,*1*}
N2^…^O(=C12)	2.69 ± 0.02 Å	2.72 ± 0.02 Å	2.78 ± 0.01 Å	2.74 Å
N2-H^…^O(=C12)	166.8 ± 3.5°	160.9 ± 2.6°	161.7 ± 5.9°	151.4°
C12=O^…^H(-N2)	106.2 ± 0.8°	106.4 ± 1.3°	103.4 ± 2.1°	114.4°

**Table 6:** Final product numbers and nomenclature.

compound	Name
**9**{*1,1,1*}	cyclo(*N*,*N*′dimethylethylenediamine-glycyl-glycyl-4,5-dicarbonyl)-1*H*-imidazole
**9**{*1,1,2*}	cyclo(*N*,*N*′dimethylethylenediamine-glycyl-*S*-alanyl-4,5-dicarbonyl)-1*H*-imidazole
**9**{*1,1,3*}	cyclo(*N*,*N*′dimethylethylenediamine-glycyl*-R*-alanyl-4,5-dicarbonyl)-1*H*-imidazole
**9**{*1,1,4*}	cyclo(*N*,*N*′dimethylethylenediamine-glycyl-*S*-leucyl-4,5-dicarbonyl)-1*H*-imidazole
**9**{*1,2,1*}	cyclo(*N*,*N*′dimethylethylenediamine-*S*-alanyl-glycyl-4,5-dicarbonyl)-1*H*-imidazole
**9**{*1,2,2*}	cyclo(*N*,*N*′dimethylethylenediamine-*S*-alanyl-*S*-alanyl-4,5-dicarbonyl)-1*H*-imidazole
**9**{*1,2,3*}	cyclo(*N*,*N*′dimethylethylenediamine-*S*-alanyl-*R*-alanyl-4,5-dicarbonyl)-1*H*-imidazole
**9**{*1,2,4*}	cyclo(*N*,*N*′dimethylethylenediamine-*S*-alanyl-*S*-leucyl-4,5-dicarbonyl)-1*H*-imidazole
**9**{*1,3,1*}	cyclo(*N*,*N*′dimethylethylenediamine-*R*-alanyl-glycyl-4,5-dicarbonyl)-1*H*-imidazole
**9**{*1,3,2*}	cyclo(*N*,*N*′dimethylethylenediamine-*R*-alanyl-*S*-alanyl-4,5-dicarbonyl)-1*H*-imidazole
**9**{*1,3,3*}	cyclo(*N*,*N*′dimethylethylenediamine-*R*-alanyl-*R*-alanyl-4,5-dicarbonyl)-1*H*-imidazole
**9**{*1,3,4*}	cyclo(*N*,*N*′dimethylethylenediamine-*R*-alanyl-*S*-leucyl-4,5-dicarbonyl)-1*H*-imidazole
**9**{*1,4,1*}	cyclo(*N*,*N*′dimethylethylenediamine-*S*-leucyl-glycyl-4,5-dicarbonyl)-1*H*-imidazole
**9**{*1,4,2*}	cyclo(*N*,*N*′dimethylethylenediamine-*S*-leucyl-*S*-alanyl-4,5-dicarbonyl)-1*H*-imidazole
**9**{*1,4,3*}	cyclo(*N*,*N*′dimethylethylenediamine-*S*-leucyl-*R*-alanyl-4,5-dicarbonyl)-1*H*-imidazole
**9**{*1,4,4*}	cyclo(*N*,*N*′dimethylethylenediamine-*S*-leucyl-*S*-leucyl-4,5-dicarbonyl)-1*H*-imidazole
**9**{*2,1,1*}	cyclo(*N*,*N*′diethylethylenediamine-glycyl-glycyl-4,5-dicarbonyl)-1*H*-imidazole
**9**{*2,1,2*}	cyclo(*N*,*N*′diethylethylenediamine-glycyl-*S*-alanyl-4,5-dicarbonyl)-1*H*-imidazole
**9**{*2,1,3*}	cyclo(*N*,*N*′diethylethylenediamine)-glycyl*-R*-alanyl-4,5-dicarbonyl)-1*H*-imidazole
**9**{*2,1,4*}	cyclo(*N*,*N*′diethylethylenediamine-glycyl-*S*-leucyl-4,5-dicarbonyl)-1*H*-imidazole
**9**{*2,2,1*}	cyclo(*N*,*N*′diethylethylenediamine-*S*-alanyl-glycyl-4,5-dicarbonyl)-1*H*-imidazole
**9**{*2,2,2*}	cyclo(*N*,*N*′diethylethylenediamine-*S*-alanyl-*S*-alanyl-4,5-dicarbonyl)-1*H*-imidazole
**9**{*2,2,3*}	cyclo(*N*,*N*′diethylethylenediamine-*S*-alanyl-*R*-alanyl-4,5-dicarbonyl)-1*H*-imidazole
**9**{*2,2,4*}	cyclo(*N*,*N*′diethylethylenediamine-*S*-alanyl-*S*-leucyl-4,5-dicarbonyl)-1*H*-imidazole
**9**{*2,3,1*}	cyclo(*N*,*N*′diethylethylenediamine-*R*-alanyl-glycyl-4,5-dicarbonyl)-1*H*-imidazole
**9**{*2,3,2*}	cyclo(*N*,*N*′diethylethylenediamine-*R*-alanyl-*S*-alanyl-4,5-dicarbonyl)-1*H*-imidazole
**9**{*2,3,3*}	cyclo(*N*,*N*′diethylethylenediamine-*R*-alanyl-*R*-alanyl-4,5-dicarbonyl)-1*H*-imidazole
**9**{*2,3,4*}	cyclo(*N*,*N*′diethylethylenediamine-*R*-alanyl-*S*-leucyl-4,5-dicarbonyl)-1*H*-imidazole
**9**{*2,4,1*}	cyclo(*N*,*N*′diethylethylenediamine-*S*-leucyl-glycyl-4,5-dicarbonyl)-1*H*-imidazole
**9**{*2,4,2*}	cyclo(*N*,*N*′diethylethylenediamine-*S*-leucyl-*S*-alanyl-4,5-dicarbonyl)-1*H*-imidazole
**9**{*2,4,3*}	cyclo(*N*,*N*′diethylethylenediamine-*S*-leucyl-*R*-alanyl-4,5-dicarbonyl)-1*H*-imidazole
**9**{*2,4,4*}	cyclo(*N*,*N*′diethylethylenediamine-*S*-leucyl-*S*-leucyl-4,5-dicarbonyl)-1*H*-imidazole

## Supplementary Materials

Supplementary materials can be accessed at: http://www.mdpi.com/1420-3049/17/5/5346/s1.
