# Supplementary material for: Parallel Synthesis of Peptide-Like Macrocycles Containing Imidazole-4,5-dicarboxylic Acid
Source: Molecules. 2012 May 8;17(5):5346–62. doi: 10.3390/molecules17055346 (PMC6268944; doi:10.3390/molecules17055346)

Supplemental Material

Page S1. Table of Contents.

Experimental Section

Page S2. Synthesis of **2**{*1*} and **2**{*2*}.
Page S2–S4. Synthesis of **4**{*1-2,1-4*}.
Page S4–S5. Synthesis of **5**{*1-2,1-4*}.
Page S5. Synthesis of **6**{*1-4*}.
Page S5–S11. Synthesis of **7**{*1-2,1-4,1-4*}.
Page S11–S13. Synthesis of **8**{*1-2,1-4,1-4*}.
Page S13–S17. Synthesis of **9**{*1-2,1-4,1-4*}.

Supporting Analytical Data

Page S17. **Figure S1.** LC-MS of **9**{*1,1,1*}.
Page S18. **Figure S2.** ^{1}H-NMR Spectra of **9**{*1,1,1*}.
Page S19. **Figure S3.** LC-MS of **9**{*1,1,2*}.
Page S20. **Figure S4.** ^{1}H-NMR Spectra of **9**{*1,1,2*}.
Page S21. **Figure S5.** LC-MS of **9**{*1,1,3*}.
Page S22. **Figure S6.** ^{1}H-NMR Spectra of **9**{*1,1,3*}.
Page S23. **Figure S7.** LC-MS of **9**{*1,1,4*}.
Page S24. **Figure S8.** ^{1}H-NMR Spectra of **9**{*1,1,4*}.
Page S25. **Figure S9.** LC-MS of **9**{*1,2,2*}.
Page S26. **Figure S10.** ^{1}H-NMR Spectra of **9**{*1,2,2*}.
Page S27. **Figure S11.** LC-MS of **9**{*1,2,4*}.
Page S28. **Figure S12.** ^{1}H-NMR Spectra of **9**{*1,2,4*}.
Page S29. **Figure S13.** LC-MS of **9**{*1,3,2*}.
Page S30. **Figure S14.** ^{1}H-NMR Spectra of **9**{*1,3,2*}.
Page S31. **Figure S15.** LC-MS of **9**{*1,3,4*}.
Page S32. **Figure S16.** ^{1}H-NMR Spectra of **9**{*1,3,4*}.
Page S33. **Figure S17.** LC-MS of **9**{*1,4,2*}.
Page S34. **Figure S18.** ^{1}H-NMR Spectra of **9**{*1,4,2*}.
Page S35. **Figure S19.** LC-MS of **9**{*1,4,3*}.
Page S36. **Figure S20.** ^{1}H-NMR Spectra of **9**{*1,4,3*}.
Page S37. **Figure S21.** LC-MS of **9**{*2,1,2*}.
Page S38. **Figure S22.** ^{1}H-NMR Spectra of **9**{*2,1,2*}.
Page S39. **Figure S23.** LC-MS of **9**{*2,1,3*}.
Page S40. **Figure S24.** ^{1}H-NMR Spectra of **9**{*2,1,3*}.
Page S41. **Figure S25.** LC-MS of **9**{*2,2,2*}.
Page S42. **Figure S26.** ^{1}H-NMR Spectra of **9**{*2,2,2*}.
Page S43. **Figure S27.** LC-MS of **9**{*2,2,3*}.
Page S44. **Figure S28.** ^{1}H-NMR Spectra of **9**{*2,2,3*}.
Page S45. **Figure S29.** LC-MS of **9**{*2,3,1*}.
Page S46. **Figure S30.** ^{1}H-NMR Spectra of **9**{*2,3,1*}.
Page S47. **Figure S31.** LC-MS of **9**{*2,3,2*}.
Page S48. **Figure S32.** ^{1}H-NMR Spectra of **9**{*2,3,2*}.
Page S49. **Figure S33.** LC-MS of **9**{*2,3,3*}.
Page S50. **Figure S34.** ^{1}H-NMR Spectra of **9**{*2,3,3*}.
Page S51. **Figure S35.** LC-MS of **9**{*2,4,1*}.
Page S52. **Figure S36.** ^{1}H-NMR Spectra of **9**{*2,4,1*}.
Page S53. **Figure S37.** LC-MS of **9**{*2,4,2*}.
Page S54. **Figure S38.** ^{1}H-NMR Spectra of **9**{*2,4,2*}.

Synthesis

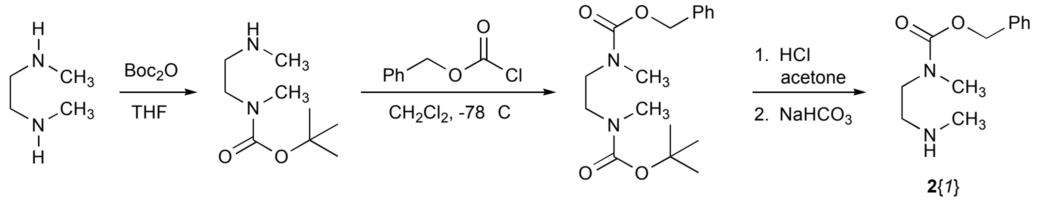

2{*1*}

2{*1*}. Alternative procedure: To an ice-cooled solution of *N*,*N*'-dimethylethylenediamine (7.4 mL, 68.8 mmol) in dry THF (60 mL) was added a solution of Boc$_2$O (5.0 g, 22.9 mmol) in dry THF (40 mL). The reaction mixture was stirred at 0 °C for 30 min. then warmed to rt and stirred another 19.5 h. The reaction mixture was filtered and the filtrate concentrated under vacuum. The residue was dissolved in EtOAc, washed with brine (3 × 50 mL), dried over MgSO$_4$, and concentrated under vacuum to yield 3.83 g (85%) of a colorless oil used without further purification. LC-MS = 1.19 min; ESI MS *m*/*z* 189 $[M+H]^+$.

The residue from above was dissolved in 40 mL of dry THF and cooled to 0 °C before concurrently adding Et$_3$N (2.84 mL, 20.4 mmol) and benzyl chloroformate (2.86 mL, 20.4 mmol) dropwise over 20 min. The reaction was warmed to rt and stirred another 3 h. The solvent was removed under vacuum. The residue was dissolved in EtOAc (100 mL), washed with brine (3 × 30 mL), dried over MgSO$_4$, and concentrated under vacuum to yield 6.35 g (97%) of a colorless oil. TLC R_f (EtOAc/Hex 1:1) = 0.54; ^{1}H-NMR (CDCl$_3$) δ 7.32 (m, 5H), 5.11 (s, 2H), 3.30 (s, 4H), 2.58 (s, 4H), 1.70 (m, 2H), 1.48 (s, 1H), 1.06 (m, 6H); LC-MS = 5.25 min; ESI MS *m*/*z* 345 $[M+Na]^+$.

To a solution of the above intermediate (6.35 g, 19.7 mmol) in 20 mL of acetone was added 36% aqueous HCl (20 mL) dropwise at rt. The reaction mixture was stirred for 16 h before removing the solvent under vacuum. The residue was dissolved in EtOAc (100 mL), washed with sat. NaHCO$_3$, brine, dried over MgSO$_4$, and concentrated under vacuum to yield 1.20 g (27%) of a pale yellow oil. LC-MS = 1.19 min; ESI MS *m*/*z* 223 $[M+H]^+$.

2{*2*}. To a dry roundbottom flask was added 40 mL of CH$_2$Cl$_2$ and *N*,*N*'-diethylethylenediamine (10.1 mL, 70.4 mmol). To this stirred solution at −78 °C was added a solution of benzyl chloroformate (1.0 mL, 7.04 mmol) in 40 mL of CH$_2$Cl$_2$ over 1 h. The reaction was stirred for 1.5 h at −78 °C before warming to rt and stirring for another 18 h. The reaction was filtered and the filtrate washed with brine (3 × 50 mL), dried over MgSO$_4$, and concentrated under vacuum. The product was purified by column chromatography using a gradient of EtOAc/MeOH (19:1 → 3:2), yielding 1.40 g (80%) of a brown oil. TLC R_f (EtOAc) = 0.05; ^{1}H-NMR (CDCl$_3$) δ 7.27–7.33 (m, 5H), 5.11 (s, 2H), 3.32 (m, 4H), 2.59–2.76 (m, 4H), 1.00–1.12 (m, 6H); LC-MS = 1.45 min; ESI MS *m*/*z* 251 $[M+H]^+$.

4{*1-2,1-4*}. To a solution of the *Z*-*N*,*N*'-dialkylalkanediamine in CH$_2$Cl$_2$ was added the Boc-amino acid, EDC•HCl, and DMAP at rt. The reaction was stirred for a period of time before removing the solvent under vacuum. The product was purified by column chromatography on silica gel.

4{*1,1*}. As described using **2**{*1*} (2.00 g, 9.01 mmol) in 100 mL of CH_2Cl_2 with **3**{*1*} (1.58 g, 9.01 mmol), EDC•HCl (3.79 g, 19.82 mmol), and DMAP (0.22 g, 1.80 mmol) at rt. The reaction was stirred for 16 h and the product was purified by column chromatography on silica gel using EtOAc/hexanes (1:3 → 1:0), as the gradient, yielding 2.94 g (86%) of a colorless oil. TLC R_f (EtOAc/Hex 1:1) = 0.37; ^{1}H-NMR ($CDCl_3$) δ 7.26–7.33 (m, 5H), 5.38 (s, 1H), 5.08 (d, 2H), 3.73–3.81 (m, 2H), 3.41–3.54 (m, 4H), 2.77–2.97 (m, 6H), 1.43 (s, 9H); LC-MS = 3.49 min; ESI MS *m/z* 380 $[M+H]^+$.

4{*1,2*}. As described using **2**{*1*} (0.33 g, 1.50 mmol) in 40 mL of CH_2Cl_2 with **3**{*2*} (0.28 g, 1.50 mmol), EDC•HCl (0.63 g, 3.30 mmol), and DMAP (25 mg, 0.30 mmol) at rt. The reaction was stirred for 18.5 h and the product was purified by column chromatography on silica gel using EtOAc/hexanes (1:3 → 1:0), as the gradient, yielding 0.42 g (72%) of a colorless oil. TLC R_f (EtOAc) = 0.54; ^{1}H-NMR ($CDCl_3$) δ 7.32–7.33 (m, 5H), 5.39 (s, 1H), 5.05 (m, 2H), 4.52 (m, 1H), 3.22–3.85 (m, 4H), 2.84–3.05 (m, 6H), 1.40 (s, 9H), 1.21 (d, J = 6.8 Hz, 3H); LC-MS = 4.11 min; ESI MS *m/z* 394 $[M+H]^+$.

4{*1,3*}. As described using **2**{*1*} (0.44 g, 2.00 mmol) in 40 mL of CH_2Cl_2 with **3**{*3*} (0.38 g, 2.00 mmol), EDC•HCl (0.84 g, 4.40 mmol), and DMAP (49 mg, 0.40 mmol) at rt. The reaction was stirred for 12.5 h and the product was purified by column chromatography on silica gel using EtOAc/hexanes (1:3 → 1:0), as the gradient, yielding 0.59 g (75%) of a colorless oil. TLC R_f (EtOAc/Hex 1:1) = 0.20; ^{1}H-NMR ($CDCl_3$) δ 7.32 (m, 5H), 5.39 (s, 1H), 5.05 (m, 2H), 4.52 (m, 1H), 3.22–3.84 (m, 4H), 2.84–3.05 (m, 6H), 1.40 (s, 9H), 1.22 (d, 3H); LC-MS = 4.11 min; ESI MS *m/z* 394 $[M+H]^+$.

4{*1,4*}. As described using **2**{*1*} (0.44 g, 2.00 mmol) in 40 mL of CH_2Cl_2 with **3**{*4*} (0.50 g, 2.00 mmol), EDC•HCl (0.84 g, 4.40 mmol), and DMAP (49 mg, 0.40 mmol) at rt. The reaction was stirred for 13 h and the product was purified by column chromatography on silica gel using EtOAc/hexanes (1:3 → 1:0), as the gradient, yielding 0.81 g (93%) of a colorless oil. TLC R_f (EtOAc/Hex 1:1) = 0.28; ^{1}H-NMR ($CDCl_3$) δ 7.32 (m, 5H), 5.02–5.12 (m, 3H), 4.57 (s, 1H), 3.23–3.84 (m, 4H), 2.83–3.08 (m, 6H), 1.41–1.71 (m, 3H), 1.40 (s, 9H), 0.88–0.97 (m, 6H); LC-MS = 6.01 min; ESI MS *m/z* 436 $[M+H]^+$.

4{*2,1*}. As described using **2**{*2*} (0.70 g, 2.80 mmol) in 40 mL of CH_2Cl_2 with **3**{*1*} (0.49 g, 2.80 mmol), EDC•HCl (1.18 g, 6.16 mmol), and DMAP (68 mg, 0.56 mmol) at rt. The reaction was stirred for 15 h and the product was purified by column chromatography on silica gel using EtOAc/hexanes (1:3 → 1:0), as the gradient, yielding 1.05 g (92%) of a colorless oil. TLC R_f (EtOAc/Hex 1:1) = 0.34; ^{1}H-NMR ($CDCl_3$) δ 7.32 (s, 5H), 5.43 (s, 1H), 5.10 (s, 2H), 3.82 (m, 2H), 3.04–3.47 (m, 8H), 1.42 (s, 9H), 0.98–1.15 (m, 6H); LC-MS = 5.03 min; ESI MS *m/z* 408 $[M+H]^+$.

4{*2,2*}. As described using **2**{*2*} (0.70 g, 2.80 mmol) in 40 mL of CH_2Cl_2 with **3**{*2*} (0.53 g, 2.80 mmol), EDC•HCl (1.18 g, 6.16 mmol), and DMAP (68 mg, 0.56 mmol) at rt. The reaction was stirred for 17 h and the product was purified by column chromatography on silica gel using EtOAc/hexanes (1:3 → 1:0), as the gradient, yielding 1.06 g (90%) of a colorless oil. TLC R_f (EtOAc/Hex 1:1) = 0.42; ^{1}H-NMR ($CDCl_3$) δ 7.32 (s, 5H), 5.33 (s, 1H), 5.10 (s, 2H), 4.52 (s, 1H), 3.19–3.64 (m, 8H), 1.40 (s, 9H), 0.97–1.25 (m, 9H); LC-MS = 4.82 min; ESI MS *m/z* 422 $[M+H]^+$.

4{*2,3*}. As described using **2**{*2*} (0.70 g, 2.80 mmol) in 40 mL of CH_2Cl_2 with **3**{*3*} (0.53 g, 2.80 mmol), EDC•HCl (1.18 g, 6.16 mmol), and DMAP (68 mg, 0.56 mmol) at rt. The reaction was stirred for 18 h and the product was purified by column chromatography on silica gel using EtOAc/hexanes (1:3 → 1:0), as the gradient, yielding 1.01 g (86%) of a colorless oil. TLC R_f (EtOAc/Hex 1:1) = 0.42; ^{1}H-NMR ($CDCl_3$) δ 7.32 (s, 5H), 5.33 (s, 1H), 5.10 (s, 2H), 4.52 (s, 1H), 3.19–3.64 (m, 8H), 1.40 (s, 9H), 0.97–1.25 (m, 9H); LC-MS = 4.82 min; ESI MS *m*/*z* 422 $[M+H]^+$.

4{*2,4*}. As described using **2**{*2*} (0.70 g, 2.80 mmol) in 40 mL of CH_2Cl_2 with **3**{*4*} (0.70 g, 2.80 mmol), EDC•HCl (1.18 g, 6.16 mmol), and DMAP (68 mg, 0.56 mmol) at rt. The reaction was stirred for 18 h and the product was purified by column chromatography on silica gel using EtOAc/hexanes (1:3 → 1:0), as the gradient, yielding 1.10 g (85%) of a colorless oil. TLC R_f (EtOAc/Hex 1:1) = 0.55; ^{1}H-NMR ($CDCl_3$) δ 7.27–7.33 (m, 5H), 5.10 (m, 3H), 4.56 (s, 1H), 3.25–3.63 (m, 8H), 1.68 (s, 1H), 1.40 (s, 9H), 1.23 (m, 2H), 0.89–1.11 (m, 12H); LC-MS = 6.93 min; ESI MS *m*/*z* 464 $[M+H]^+$.

5{*1-2,1-4*}. A suspension of one intermediate, **4**{*1-2,1-4*}, and 5% Pd/C in CH_3OH was stirred at rt under 1 atm of H_2. The reaction was stirred until complete, filtered to remove the Pd/C, and concentrated under vacuum. The product was used without further purification.

5{*1,1*}. As described using **4**{*1,1*} (2.90 g, 7.65 mmol) and 5% Pd/C (0.40 g) in 120 mL of CH_3OH at rt. The reaction was stirred for 11 h and workup yielded 1.80 g (96%) of a colorless oil. ^{1}H-NMR ($CDCl_3$) δ 5.49 (s, 1H), 3.92 (q, *J* = 8.0 Hz, 2H), 3.49 (t, *J* = 6.4 Hz, 1H), 3.30 (t, *J* = 6.4 Hz, 1H), 2.94 (d, *J* = 2.8 Hz, 3H), 2.72 (m, 2H), 2.41 (d, *J* = 3.6 Hz, 3H), 1.42 (s, 9H), 1.28 (s, 1H); LC-MS = 1.20 min; ESI MS *m*/*z* 246 $[M+H]^+$.

5{*1,2*}. As described using **4**{*1,2*} (0.56 g, 1.43 mmol) and 5% Pd/C (0.10 g) in 40 mL of CH_3OH at rt. The reaction was stirred for 10.5 h and workup yielded 0.35 g (95%) of a colorless oil. ^{1}H-NMR ($CDCl_3$) δ 5.48 (m, 1H), 4.43 (m, 1H), 3.23–3.46 (m, 2H), 2.92 (d, 3H), 2.65 (m, 2H), 2.28 (d, *J* = 6.8 Hz, 3H), 1.72 (s, 1H), 1.27 (s, 9H), 1.15 (dd, *J* = 2.4, 6.8 Hz, 3H); LC-MS = 1.15 min; ESI MS *m*/*z* 260 $[M+H]^+$.

5{*1,3*}. As described using **4**{*1,3*} (0.59 g, 1.50 mmol) and 5% Pd/C (0.10 g) in 40 mL of CH_3OH at rt. The reaction was stirred for 10.5 h and workup yielded 0.38 g (98%) of a colorless oil. ^{1}H-NMR ($CDCl_3$) δ 5.48 (m, 1H), 4.43 (m, 1H), 3.23–3.46 (m, 2H), 2.92 (d, 3H), 2.65 (m, 2H), 2.28 (d, *J* = 6.8 Hz, 3H), 1.72 (s, 1H), 1.27 (s, 9H), 1.15 (dd, *J* = 2.4, 6.8 Hz, 3H); LC-MS = 1.15 min; ESI MS *m*/*z* 260 $[M+H]^+$.

5{*1,4*}. As described using **4**{*1,4*} (0.81 g, 1.86 mmol) and 5% Pd/C (0.10 g) in 40 mL of CH_3OH at rt. The reaction was stirred for 10.5 h and workup yielded 0.54 g (96%) of a colorless oil. ^{1}H-NMR ($CDCl_3$) δ 9.28 (d, 1H), 5.50 (d, *J* = 8.0 Hz, 1H), 4.49 (m, 1H), 3.62–3.95 (m, 4H), 3.16 (s, 3H), 2.70 (s, 3H), 1.67 (m, 1H), 1.41–1.49 (m, 2H), 1.38 (s, 9H), 0.89 (q, *J* = 6.8 Hz, 6H); LC-MS = 1.20 min; ESI MS *m*/*z* 302 $[M+H]^+$.

5{*2,1*}. As described using **4**{*2,1*} (1.00 g, 2.46 mmol) and 5% Pd/C (0.10 g) in 40 mL of CH_3OH at rt. The reaction was stirred for 17.5 h and workup yielded 0.67 g (100%) of a colorless oil. ^{1}H-NMR

($CDCl_3$) δ 5.52 (s, 1H), 3.95 (m, 2H), 3.21–3.46 (m, 4H), 2.5 5–2.78 (m, 4H), 1.42 (s, 9H), 1.26 (s, 1H), 0.95–1.18 (m, 6H); LC-MS = 1.11 min; ESI MS *m/z* 274 $[M+H]^+$.

5{*2,2*}. As described using **4**{*2,2*} (1.02 g, 2.42 mmol) and 5% Pd/C (0.10 g) in 40 mL of CH_3OH at rt. The reaction was stirred for 17.5 h and workup yielded 0.64 g (97%) of a colorless oil. ^{1}H-NMR ($CDCl_3$) δ 5.36 (s, 1H), 4.56 (m, 1H), 3.21–3.55 (m, 4H), 2.61–2.83 (m, 4H), 1.41 (s, 9H), 1.28 (m, 3H), 1.05–1.21 (m, 6H); LC-MS = 1.13 min; ESI MS *m/z* 288 $[M+H]^+$.

5{*2,3*}. As described using **4**{*2,3*} (1.00 g, 2.38 mmol) and 5% Pd/C (0.10 g) in 40 mL of CH_3OH at rt. The reaction was stirred for 17.5 h and workup yielded 0.63 g (92%) of a colorless oil. ^{1}H-NMR ($CDCl_3$) δ 5.36 (s, 1H), 4.56 (m, 1H), 3.21–3.55 (m, 4H), 2.61–2.83 (m, 4H), 1.41 (s, 9H), 1.28 (m, 3H), 1.05–1.21 (m, 6H); LC-MS = 1.13 min; ESI MS *m/z* 288 $[M+H]^+$.

5{*2,4*}. As described using **4**{*2,4*} (1.06 g, 2.29 mmol) and 5% Pd/C (0.10 g) in 40 mL of CH_3OH at rt. The reaction was stirred for 17.5 h and workup yielded 0.71 g (94%) of a colorless oil. ^{1}H-NMR ($CDCl_3$) δ 5.13 (d, *J* = 9.2 Hz, 1H), 4.58 (q, *J* = 6.0 Hz, 1H), 3.18–3.57 (m, 4H), 2.61–2.85 (m, 4H), 1.70 (m, 1H), 1.50 (m, 2H), 1.40 (s, 9H), 1.05–1.24 (m, 6H), 0.90 (q, *J* = 6.8 Hz, 6H); LC-MS = 1.48 min; ESI MS *m/z* 330 $[M+H]^+$.

5,10-dioxo-5*H*,10*H*-diimidazo{1,5-a:1',5'-d}pyrazine-1,6-dicarbonyl Amino Acid Esters, **6{*1-4*}**. Briefly, to a dry round-bottom flask under argon was added dry CH_2Cl_2. To this stirred solvent at −78 °C were added, in order, the pyrazine diacid chloride (1 equiv.), the amino acid ester hydrochloride salt (2 equiv.), and *N,N*-diethylaniline (4 equiv.). The resulting solution was held at −78 °C for 30 min before stirring at room temperature. The reaction was washed against water (3×) and the organic fraction was dried over $MgSO_4$, filtered and concentrated. The residue was suspended in boiling EtOAc, stirred for 15 min and then cooled at 0 °C. The solid product **6{*1-4*}** was collected by vacuum filtration and washed with EtOAc. The final product was characterized by ^{1}H-NMR spectroscopy.

6{*1*}. ^{1}H-NMR ($CDCl_3$) δ 8.64 (s, 2H), 8.59 (t, *J* = 4.8 Hz, 2H), 4.17 (d, *J* = 4.8 Hz, 2H), 1.49 (s, 18H).

6{*2*}. ^{1}H-NMR ($CDCl_3$) δ 8.68 (d, *J* = 7.2 Hz, 2H), 8.63 (s, 2H), 4.70 (dt, *J* = 6.8, 7.2 Hz, 2H), 1.51 (d, *J* = 6.8 Hz, 6H), 1.48 (s, 18H).

6{*3*}. ^{1}H-NMR ($CDCl_3$) δ 8.68 (d, *J* = 7.2 Hz, 2H), 8.63 (s, 2H), 4.70 (dt, *J* = 6.8, 7.2 Hz, 2H), 1.51 (d, *J* = 6.8 Hz, 6H), 1.48 (s, 18H).

6{*4*}. ^{1}H-NMR ($CDCl_3$) δ 8.63 (s, 2H), 8.50 (d, *J* = 8.4 Hz, 2H), 4.76 (m, 2H), 1.75 (m, 6H), 1.47 (s, 18H), 0.97 (d, *J* = 6.0 Hz, 12H).

7{*1-2,1-4,1-4*}. To a suspension of one amino acid ester substituted pyrazine, **6**{*1-4*} in CH_2Cl_2 was added one intermediate, **5**{*1-2,1-4*} at rt. The reaction was stirred until complete, the solvent removed under vacuum, and the product purified by column chromatography on silica gel.

7{*1,1,1*}. As described using **6**{*1*} (0.20 g, 0.40 mmol) and **5**{*1,1*} (0.25 g, 1.00 mmol) in 20 mL of CH_2Cl_2 at rt. The reaction was stirred for 19.5 h. Column chromatography using a gradient of

EtOAc/Hex (1:1 → 1:0) followed by EtOAc/MeOH (1:19 → 4:1) yielded 0.25 g (63%) of a white solid. TLC R_f (EtOAc/MeOH 9:1) = 0.25; ^{1}H-NMR ($CDCl_3$) δ 12.26 (m, 1H), 10.5 0–10.93 (m, 1H), 7.58 (d, *J* = 5.6 Hz, 1H), 5.56 (t, *J* = 11.2 Hz, 1H), 3.64–4.19 (m, 8H), 2.85–3.42 (m, 6H), 1.47 (s, 9H), 1.39 (s, 9H); ^{13}C-NMR ($CDCl_3$) δ 169.0, 168.9, 168.4, 168.3, 168.2, 168.1, 165.9, 165.8, 165.4, 159.7, 159.5, 158.6, 155.8, 136.5, 135.4, 134.8, 134.6, 134.5, 134.4, 133.8, 130.7, 129.7, 129.3, 129.2, 129.0, 81.9, 79.5, 79.3, 52.9, 49.5, 48.6, 47.9, 47.2, 46.7, 46.6, 45.9, 45.4, 42.6, 42.4, 42.3, 41.9, 41.6, 39.0, 38.6, 35.9, 35.6, 35.0, 34.5, 34.2, 34.0, 28.3, 28.0; LC-MS = 1.67 min; ESI MS *m*/*z* 497 [M+H]$^+$.

7{*1,1,2*}. As described using **6**{*2*} (0.21 g, 0.40 mmol) and **5**{*1,1*} (0.25 g, 1.00 mmol) in 20 mL of CH_2Cl_2 at rt. The reaction was stirred for 19.5 h. Column chromatography using a gradient of EtOAc/Hex (1:1 → 1:0) followed by EtOAc/MeOH (1:19 → 4:1) yielded 0.30 g (75%) of a white solid. TLC R_f (EtOAc/MeOH 9:1) = 0.28; ^{1}H-NMR ($CDCl_3$) δ 12.22 (m, 1H), 10.57–10.96 (m, 1H), 7.58 (d, *J* = 7.6 Hz, 1H), 5.54 (d, 1H), 4.53 (m, 1H), 3.69–4.19 (m, 6H), 2.90–3.42 (m, 6H), 1.55 (m, 3H), 1.44 (s, 9H), 1.38 (s, 9H); ^{13}C-NMR ($CDCl_3$) δ 171.4, 171.3, 169.0, 168.9, 165.8, 165.4, 159.1, 159.0, 158.9, 158.8, 155.8, 136.3, 135.3, 134.7, 134.6, 134.5, 134.4, 133.8, 130.9, 130.7, 129.9, 129.4, 129.2, 82.0, 81.6, 79.5, 52.9, 49.5, 49.4, 48.6, 47.3, 46.7, 45.5, 42.3, 38.6, 36.0, 35.6, 35.0, 34.6, 34.2, 34.0, 28.3, 28.0, 18.0, 17.8; LC-MS = 1.91 min; ESI MS *m*/*z* 511 [M+H]$^+$.

7{*1,1,3*}. As described using **6**{*3*} (0.21 g, 0.40 mmol) and **5**{*1,1*} (0.25 g, 1.00 mmol) in 20 mL of CH_2Cl_2 at rt. The reaction was stirred for 19.5 h. Column chromatography using a gradient of EtOAc/Hex (1:1 → 1:0) followed by EtOAc/MeOH (1:19 → 4:1) yielded 0.29 g (70%) of a white solid. TLC R_f (EtOAc/MeOH 9:1) = 0.28; ^{1}H-NMR ($CDCl_3$) δ 12.22 (m, 1H), 10.57–10.96 (m, 1H), 7.58 (d, *J* = 7.6 Hz, 1H), 5.54 (d, 1H), 4.53 (m, 1H), 3.69–4.19 (m, 6H), 2.90–3.42 (m, 6H), 1.55 (m, 3H), 1.44 (s, 9H), 1.38 (s, 9H); ^{13}C-NMR ($CDCl_3$) δ 171.4, 171.3, 169.0, 168.9, 165.8, 165.4, 159.1, 159.0, 158.9, 158.8, 155.8, 136.3, 135.3, 134.7, 134.6, 134.5, 134.4, 133.8, 130.9, 130.7, 129.9, 129.4, 129.2, 82.0, 81.6, 79.5, 52.9, 49.5, 49.4, 48.6, 47.3, 46.7, 45.5, 42.3, 38.6, 36.0, 35.6, 35.0, 34.6, 34.2, 34.0, 28.3, 28.0, 18.0, 17.8; LC-MS = 1.91 min; ESI MS *m*/*z* 511 [M+H]$^+$.

7{*1,1,4*}. As described using **6**{*4*} (0.25 g, 0.40 mmol) and **5**{*1,1*} (0.25 g, 1.00 mmol) in 20 mL of CH_2Cl_2 at rt. The reaction was stirred for 19.5 h. Column chromatography using a gradient of EtOAc/Hex (1:1 → 1:0) followed by EtOAc/MeOH (1:19 → 4:1) yielded 0.25 g (57%) of a white solid. TLC R_f (EtOAc/MeOH 9:1) = 0.32; ^{1}H-NMR ($CDCl_3$) δ 12.09 (m, 1H), 10.48–10.88 (m, 1H), 7.56 (m, 1H), 5.52 (d, 1H), 4.53 (m, 1H), 3.63–4.18 (m, 6H), 2.94–3.44 (m, 6H), 1.65–1.80 (m, 3H), 1.46 (s, 9H), 1.39 (s, 9H), 0.95 (m, 6H); ^{13}C-NMR ($CDCl_3$) δ 171.4, 171.3, 171.2, 168.9, 165.8, 165.7, 165.4, 165.3, 159.2, 158.9, 158.2, 155.8, 136.2, 135.0, 134.7, 134.6, 134.4, 133.8, 130.9, 130.7, 130.0, 129.7, 129.4, 129.3, 81.9, 81.5, 79.5, 52.9, 52.4, 52.3, 49.6, 48.6, 47.3, 46.8, 45.5, 42.3, 41.4, 41.2, 41.0, 38.8, 36.0, 35.7, 35.0, 34.7, 34.0, 28.4, 28.3, 28.0, 25.0, 22.8, 22.1, 22.0; LC-MS = 3.88 min; ESI MS *m*/*z* 553 [M+H]$^+$.

7{*1,2,1*}. As described using **6**{*1*} (73 mg, 0.146 mmol) and **5**{*1,2*} (83 mg, 0.320 mmol) in 4 mL of CH_2Cl_2 at rt. The reaction was stirred for 19.5 h. Column chromatography using a gradient of EtOAc/Hex (1:1 → 1:0) followed by EtOAc/MeOH (1:19 → 4:1) yielded 129 mg (86%) of a white solid. TLC R_f (EtOAc) = 0.20; LC-MS = 2.41 min; ESI MS *m*/*z* 511 [M+H]$^+$.

7{*1,2,2*}. As described using **6**{*2*} (174 mg, 0.328 mmol) and **5**{*1,2*} (170 mg, 0.656 mmol) in 10 mL of CH_2Cl_2 at rt. The reaction was stirred for 3.5 h. Column chromatography using a gradient of EtOAc/Hex (1:1 → 1:0) followed by EtOAc/MeOH (1:19 → 4:1) yielded 295 mg (86%) of a white solid. TLC R_f (EtOAc/MeOH 9:1) = 0.58; ^{1}H-NMR ($CDCl_3$) δ 12.28 (s, 1H), 10.61–10.96 (m, 1H), 7.58 (d, *J* = 8.0 Hz, 1H), 5.53 (d, *J* = 6.0 Hz, 1H), 4.52 (m, 2H), 3.52–4.13 (m, 4H), 2.97–3.44 (m, 6H), 1.48 (m, 3H), 1.44 (s, 9H), 1.38 (s, 9H), 1.21 (m, 3H); LC-MS = 2.93 min; ESI MS *m/z* 525 $[M+H]^+$.

7{*1,2,3*}. As described using **6**{*3*} (77 mg, 0.146 mmol) and **5**{*1,2*} (83 mg, 0.320 mmol) in 4 mL of CH_2Cl_2 at rt. The reaction was stirred for 19.5 h. Column chromatography using a gradient of EtOAc/Hex (1:1 → 1:0) followed by EtOAc/MeOH (1:19 → 4:1) yielded 136 mg (89%) of a white solid. TLC R_f (EtOAc) = 0.25; LC-MS = 2.93 min; ESI MS *m/z* 525 $[M+H]^+$.

7{*1,2,4*}. As described using **6**{*4*} (90 mg, 0.146 mmol) and **5**{*1,2*} (83 mg, 0.320 mmol) in 4 mL of CH_2Cl_2 at rt. The reaction was stirred for 19.5 h. Column chromatography using a gradient of EtOAc/Hex (1:1 → 1:0) followed by EtOAc/MeOH (1:19 → 4:1) yielded 140 mg (85%) of a white solid. TLC R_f (EtOAc) = 0.38; LC-MS = 4.29 min; ESI MS *m/z* 567 $[M+H]^+$.

7{*1,3,1*}. As described using **6**{*1*} (84 mg, 0.167 mmol) and **5**{*1,3*} (95 mg, 0.367 mmol) in 4 mL of CH_2Cl_2 at rt. The reaction was stirred for 19.5 h. Column chromatography using a gradient of EtOAc/Hex (1:1 → 1:0) followed by EtOAc/MeOH (1:19 → 4:1) yielded 57 mg (34%) of a white solid. TLC R_f (EtOAc) = 0.15; LC-MS = 1.60 min; ESI MS *m/z* 511 $[M+H]^+$.

7{*1,3,2*}. As described using **6**{*2*} (89 mg, 0.167 mmol) and **5**{*1,3*} (95 mg, 0.367 mmol) in 4 mL of CH_2Cl_2 at rt. The reaction was stirred for 19.5 h. Column chromatography using a gradient of EtOAc/Hex (1:1 → 1:0) followed by EtOAc/MeOH (1:19 → 4:1) yielded 47 mg (27%) of a white solid. TLC R_f (EtOAc) = 0.23; LC-MS = 1.98 min; ESI MS *m/z* 525 $[M+H]^+$.

7{*1,3,3*}. As described using **6**{*3*} (89 mg, 0.167 mmol) and **5**{*1,3*} (95 mg, 0.367 mmol) in 4 mL of CH_2Cl_2 at rt. The reaction was stirred for 19.5 h. Column chromatography using a gradient of EtOAc/Hex (1:1 → 1:0) followed by EtOAc/MeOH (1:19 → 4:1) yielded 42 mg (24%) of a white solid. TLC R_f (EtOAc) = 0.23; ^{1}H-NMR ($CDCl_3$) δ 12.60 (d, 1H), 10.58–10.95 (m, 1H), 7.53 (d, *J* = 4.4 Hz, 1H), 5.58 (t, *J* = 8.0 Hz, 1H), 4.50 (m, 2H), 3.48–4.00 (m, 4H), 2.93–3.35 (m, 6H), 1.48 (m, 3H), 1.39 (s, 9H), 1.31 (s, 9H), 1.16 (m, 3H); ^{13}C-NMR ($CDCl_3$) δ 173.4, 173.2, 171.3, 171.2, 165.8, 165.7, 165.4, 165.3, 159.0, 158.9, 155.1, 154.9, 135.2, 134.7, 134.6, 134.5, 133.9, 129.6, 129.4, 129.3, 129.0, 81.4, 79.2, 79.1, 49.8, 49.4, 49.3, 48.4, 47.9, 46.9, 46.5, 46.2, 46.0, 45.4, 38.6, 35.9, 35.8, 35.6, 34.0, 29.5, 28.2, 27.9, 19.4, 18.4, 17.9, 17.8; LC-MS = 1.98 min; ESI MS *m/z* 525 $[M+H]^+$.

7{*1,3,4*}. As described using **6**{*4*} (103 mg, 0.167 mmol) and **5**{*1,3*} (95 mg, 0.367 mmol) in 4 mL of CH_2Cl_2 at rt. The reaction was stirred for 19.5 h. Column chromatography using a gradient of EtOAc/Hex (1:1 → 1:0) followed by EtOAc/MeOH (1:19 → 4:1) yielded 51 mg (45%) of a white solid. TLC R_f (EtOAc) = 0.38; ^{1}H-NMR ($CDCl_3$) δ 12.67 (m, 1H), 10.60–10.89 (q, *J* = 6.8 Hz, 1H), 7.54 (d, *J* = 7.2 Hz, 1H), 5.58 (m, 1H), 4.50 (m, 2H), 3.49–4.07 (m, 4H), 2.93–3.37 (m, 6H), 1.62–1.76 (m, 3H), 1.40 (s, 9H), 1.34 (s, 9H), 1.21 (m, 3H), 0.91 (t, , *J* = 7.2 Hz, 1H); ^{13}C-NMR ($CDCl_3$)

δ 173.4, 173.2, 171.3, 165.9, 165.7, 165.4, 165.3, 159.3, 159.2, 159.1, 155.1, 155.0, 135.1, 134.8, 134.6, 134.4, 134.0, 129.7, 129.5, 129.1, 81.3, 79.3, 79.2, 52.3, 52.2, 49.9, 48.5, 47.9, 47.0, 46.5, 46.3, 46.1, 45.8, 45.5, 41.2, 41.1, 41.0, 38.7, 35.9, 35.7, 34.1, 29.5, 28.2, 27.9, 27.7, 27.6, 24.9, 22.8, 22.7, 21.9, 21.8, 19.4, 18.4; LC-MS = 4.24 min; ESI MS *m*/*z* 567 $[M+H]^+$.

7{*1,4,1*}. As described using **6**{*1*} (102 mg, 0.204 mmol) and **5**{*1,4*} (135 mg, 0.449 mmol) in 4 mL of CH_2Cl_2 at rt. The reaction was stirred for 19.5 h. Column chromatography using a gradient of EtOAc/Hex (1:1 → 1:0) followed by EtOAc/MeOH (1:19 → 4:1) yielded 59 mg (26%) of a white solid. TLC R_f (EtOAc) = 0.30; LC-MS = 3.43 min; ESI MS *m*/*z* 553 $[M+H]^+$.

7{*1,4,2*}. As described using **6**{*2*} (108 mg, 0.204 mmol) and **5**{*1,4*} (135 mg, 0.449 mmol) in 4 mL of CH_2Cl_2 at rt. The reaction was stirred for 19.5 h. Column chromatography using a gradient of EtOAc/Hex (1:1 → 1:0) followed by EtOAc/MeOH (1:19 → 4:1) yielded 69 mg (30%) of a white solid. TLC R_f (EtOAc) = 0.40; LC-MS = 4.35 min; ESI MS *m*/*z* 567 $[M+H]^+$.

7{*1,4,3*}. As described using **6**{*3*} (108 mg, 0.204 mmol) and **5**{*1,4*} (135 mg, 0.449 mmol) in 4 mL of CH_2Cl_2 at rt. The reaction was stirred for 19.5 h. Column chromatography using a gradient of EtOAc/Hex (1:1 → 1:0) followed by EtOAc/MeOH (1:19 → 4:1) yielded 49 mg (21%) of a white solid. TLC R_f (EtOAc) = 0.40; LC-MS = 4.35 min; ESI MS *m*/*z* 567 $[M+H]^+$.

7{*1,4,4*}. No product isolated.

7{*2,1,1*}. As described using **6**{*1*} (139 mg, 0.277 mmol) and **5**{*2,1*} (135 mg, 0.610 mmol) in 4 mL of CH_2Cl_2 at rt. The reaction was stirred for 19 h. Column chromatography using a gradient of EtOAc/Hex (1:1 → 1:0) followed by EtOAc/MeOH (1:19 → 4:1) yielded 146 mg (50%) of a white solid. TLC R_f (EtOAc) = 0.41; ^{1}H-NMR ($CDCl_3$) δ 12.47 (d, 1H), 10.58–10.91 (m, 1H), 7.52 (d, *J* = 8.0 Hz, 1H), 5.61 (s, 1H), 3.63–4.11 (m, 6H), 3.19–3.57 (m, 6H), 1.40 (s, 9H), 1.34 (s, 9H), 1.01 (m, 6H); ^{13}C-NMR ($CDCl_3$) δ 168.5, 168.4, 168.2, 168.1, 165.6, 164.9, 164.8, 159.8, 159.7, 159.6, 155.7, 135.4, 134.9, 134.7, 134.5, 134.4, 134.2, 81.7, 79.3, 79.1, 47.4, 46.3, 46.0, 45.4, 45.0, 44.7, 44.6, 43.6, 43.2, 42.9, 42.5, 42.3, 41.9, 41.4, 41.1, 28.2, 28.0, 27.9, 27.7, 14.5, 14.2, 13.8, 13.5, 12.7, 12.5; LC-MS = 3.31 min; ESI MS *m*/*z* 525 $[M+H]^+$.

7{*2,1,2*}. As described using **6**{*2*} (143 mg, 0.277 mmol) and **5**{*2,1*} (135 mg, 0.610 mmol) in 4 mL of CH_2Cl_2 at rt. The reaction was stirred for 19 h. Column chromatography using a gradient of EtOAc/Hex (1:1 → 1:0) followed by EtOAc/MeOH (1:19 → 4:1) yielded 140 mg (47%) of a white solid. TLC R_f (EtOAc) = 0.46; ^{1}H-NMR ($CDCl_3$) δ 12.54 (d, 1H), 10.59–10.89 (m, 1H), 7.50 (m, 1H), 5.62 (s, 1H), 4.45 (m, 1H), 3.19–4.10 (m, 10H), 1.40 (s, 9H), 1.34 (s, 9H), 1.10 (m, 6H); ^{13}C-NMR ($CDCl_3$) δ 171.3, 171.2, 168.5, 165.7, 165.6, 165.0, 164.9, 159.1, 159.0, 158.9, 155.7, 135.3, 134.9, 134.7, 134.4, 134.2, 129.6, 129.3, 129.2, 128.8, 81.4, 81.3, 79.3, 79.1, 49.3, 49.2, 47.3, 46.3, 45.9, 45.5, 45.3, 45.1, 44.7, 43.6, 43.1, 42.8, 42.5, 42.3, 41.9, 41.4, 41.1, 28.1, 27.9, 27.8, 17.9, 17.8, 17.7, 14.5, 14.3, 13.8, 12.7, 12.5; LC-MS = 2.69 min; ESI MS *m*/*z* 539 $[M+H]^+$.

7{*2,1,3*}. As described using **6**{*3*} (143 mg, 0.277 mmol) and **5**{*2,1*} (135 mg, 0.610 mmol) in 4 mL of CH_2Cl_2 at rt. The reaction was stirred for 19 h. Column chromatography using a gradient of

EtOAc/Hex (1:1 → 1:0) followed by EtOAc/MeOH (1:19 → 4:1) yielded 170 mg (57%) of a white solid. TLC R_f (EtOAc) = 0.46; ^{1}H-NMR ($CDCl_3$) δ 12.58 (d, 1H), 10.57–10.87 (m, 1H), 7.50 (d, *J* = 9.2 Hz, 1H), 5.63 (s, 1H), 4.41 (m, 1H), 3.18–4.09 (m, 10H), 1.37 (s, 9H), 1.32 (s, 9H), 1.22 (m, 6H); ^{13}C-NMR ($CDCl_3$) δ 171.4, 171.3, 168.6, 168.5, 165.8, 165.7, 165.1, 165.0, 159.2, 159.1, 159.0, 155.9, 155.8, 135.4, 135.0, 134.8, 134.5, 134.3, 129.7, 129.4, 129.2, 128.9, 81.4, 79.4, 79.2, 49.4, 49.3, 47.4, 46.3, 46.0, 45.5, 45.4, 45.2, 44.8, 43.7, 43.2, 42.8, 42.6, 42.4, 42.0, 41.5, 41.2, 29.5, 28.2, 27.9, 27.7, 18.0, 17.9, 17.8, 14.6, 14.4, 13.9, 12.8, 12.6; LC-MS = 2.70 min; ESI MS *m*/*z* 539 $[M+H]^+$.

7{*2,1,4*}. No product isolated.

7{*2,2,1*}. As described using **6**{*1*} (127 mg, 0.253 mmol) and **5**{*2,2*} (160 mg, 0.557 mmol) in 4 mL of CH_2Cl_2 at rt. The reaction was stirred for 19 h. Column chromatography using a gradient of EtOAc/Hex (1:1 → 1:0) followed by EtOAc/MeOH (1:19 → 4:1) yielded 135 mg (50%) of a white solid. TLC R_f (EtOAc) = 0.49; ^{1}H-NMR ($CDCl_3$) δ 12.52 (t, 1H), 10.58–10.91 (m, 1H), 7.51 (d, *J* = 7.2 Hz, 1H), 5.58 (q, *J* = 8.0 Hz ,1H), 4.50 (m, 1H), 4.01 (d, *J* = 8.4 Hz, 2H), 3.17–3.88 (m, 8H), 1.38 (s, 9H), 1.31 (s, 9H), 1.21 (m, 9H); ^{13}C-NMR ($CDCl_3$) δ 173.2, 173.1, 172.9, 168.2, 168.1, 165.6, 165.5, 164.9, 164.8, 159.8, 159.7, 155.0, 154.9, 135.2, 134.9, 134.7, 134.4, 134.3, 129.4, 129.3, 129.0, 128.7, 81.7, 81.6, 79.2, 79.0, 47.8, 46.4, 46.0, 45.9, 45.7, 45.4, 45.3, 44.9, 44.6, 44.2, 43.6, 43.3, 43.2, 43.0, 42.8, 42.3, 41.6, 41.1, 28.2, 28.0, 27.9, 27.8, 19.3, 19.2, 18.9, 14.4, 14.3, 14.0, 12.6, 12.5; LC-MS = 2.43 min; ESI MS *m*/*z* 539 $[M+H]^+$.

7{*2,2,2*}. As described using **6**{*2*} (131 mg, 0.253 mmol) and **5**{*2,2*} (160 mg, 0.557 mmol) in 4 mL of CH_2Cl_2 at rt. The reaction was stirred for 19 h. Column chromatography using a gradient of EtOAc/Hex (1:1 → 1:0) followed by EtOAc/MeOH (1:19 → 4:1) yielded 140 mg (50%) of a white solid. TLC R_f (EtOAc) = 0.48; ^{1}H-NMR ($CDCl_3$) δ 12.57 (d, 1H), 10.62–10.88 (m, 1H), 7.51 (d, *J* = 8.0 Hz, 1H), 5.61 (m,1H), 4.46 (m, 2H), 3.19–3.97 (m, 8H), 1.39 (s, 9H), 1.32 (s, 9H), 1.21 (m, 12H); ^{13}C-NMR ($CDCl_3$) δ 173.2, 173.0, 171.3, 171.2, 165.7, 165.6, 165.0, 164.9, 159.1, 158.9, 155.1, 155.0, 135.0, 134.9, 134.7, 134.4, 129.5, 129.3, 128.8, 81.4, 81.3, 81.2, 79.2, 79.0, 49.4, 49.3, 47.8, 46.5, 46.3, 46.0, 45.8, 45.5, 45.3, 45.0, 44.6, 44.2, 43.6, 43.3, 43.2, 43.1, 42.9, 41.6, 41.1, 28.2, 27.8, 19.3, 19.2, 17.9, 17.8, 17.7, 14.4, 14.3, 12.7, 12.5; LC-MS = 3.22 min; ESI MS *m*/*z* 553 $[M+H]^+$.

7{*2,2,3*}. As described using **6**{*3*} (131 mg, 0.253 mmol) and **5**{*2,2*} (160 mg, 0.557 mmol) in 4 mL of CH_2Cl_2 at rt. The reaction was stirred for 19 h. Column chromatography using a gradient of EtOAc/Hex (1:1 → 1:0) followed by EtOAc/MeOH (1:19 → 4:1) yielded 163 mg (61%) of a white solid. TLC R_f (EtOAc) = 0.46; ^{1}H-NMR ($CDCl_3$) δ 12.58 (t, 1H), 10.61–10.90 (m, 1H), 7.50 (d, *J* = 7.6 Hz, 1H), 5.54 (d, 1H), 4.43 (m, 2H), 3.17–3.81 (m, 8H), 1.38 (s, 9H), 1.32 (s, 9H), 1.20 (m, 12H); ^{13}C-NMR ($CDCl_3$) δ 173.1, 172.9, 171.3, 171.2, 165.7, 165.5, 165.0, 164.9, 159.1, 158.9, 155.0, 154.9, 135.1, 134.9, 134.6, 134.4, 129.5, 129.2, 129.1, 128.8, 81.3, 79.2, 79.0, 49.3, 49.2, 47.8, 46.3, 46.0, 45.8, 45.5, 45.3, 44.9, 44.6, 44.1, 43.6, 43.3, 43.1, 42.9, 42.8, 41.5, 41.1, 28.2, 27.8, 27.6, 19.3, 19.2, 17.9, 17.8, 17.7, 14.4, 14.3, 12.7, 12.6, 12.5; LC-MS = 4.37 min; ESI MS *m*/*z* 553 $[M+H]^+$.

7{*2,2,4*}. No product isolated.

7{*2,3,1*}. As described using **6**{*1*} (126 mg, 0.250 mmol) and **5**{*2,3*} (158 mg, 0.549 mmol) in 4 mL of CH_2Cl_2 at rt. The reaction was stirred for 19 h. Column chromatography using a gradient of EtOAc/Hex (1:1 → 1:0) followed by EtOAc/MeOH (1:19 → 4:1) yielded 138 mg (51%) of a white solid. TLC R_f (EtOAc) = 0.49; ^{1}H-NMR ($CDCl_3$) δ 12.50 (t, 1H), 10.61–10.91 (m, 1H), 7.51 (t, J = 6.8 Hz, 1H), 5.58 (q, J = 8.0 Hz ,1H), 4.51 (m, 1H), 4.02 (d, J = 5.2 Hz, 2H), 3.16–3.89 (m, 8H), 1.39 (s, 9H), 1.32 (s, 9H), 1.21 (m, 9H); ^{13}C-NMR ($CDCl_3$) δ 173.2, 173.1, 172.9, 168.3, 168.2, 168.1, 165.7, 165.5, 165.0, 164.8, 159.8, 159.7, 155.0, 135.2, 134.9, 134.7, 134.4, 129.5, 129.3, 129.1, 128.7, 81.7, 81.6, 79.3, 79.2, 79.1, 47.9, 46.4, 46.0, 45.7, 45.5, 45.3, 44.9, 44.6, 44.2, 43.6, 43.3, 43.2, 43.1, 42.9, 42.3, 41.6, 41.1, 28.2, 27.9, 19.3, 19.2, 14.4, 14.3, 12.7, 12.5; LC-MS = 2.43 min; ESI MS *m/z* 539 $[M+H]^+$.

7{*2,3,2*}. As described using **6**{*2*} (129 mg, 0.250 mmol) and **5**{*2,3*} (158 mg, 0.549 mmol) in 4 mL of CH_2Cl_2 at rt. The reaction was stirred for 19 h. Column chromatography using a gradient of EtOAc/Hex (1:1 → 1:0) followed by EtOAc/MeOH (1:19 →4:1) yielded 150 mg (55%) of a white solid. TLC R_f (EtOAc) = 0.48; ^{1}H-NMR ($CDCl_3$) δ 12.57 (t, 1H), 10.61–10.90 (m, 1H), 7.51 (d, J = 7.6 Hz, 1H), 5.53 (d,1H), 4.46 (m, 2H), 3.21–3.83 (m, 8H), 1.38 (s, 9H), 1.32 (s, 9H), 1.20 (m, 12H); ^{13}C-NMR ($CDCl_3$) δ 173.1, 172.9, 171.3, 171.2, 165.7, 165.6, 165.0, 164.9, 159.1, 159.0, 155.0, 135.2, 135.0, 134.9, 134.6, 134.4, 129.5, 129.2, 129.1, 128.8, 81.3, 79.2, 79.0, 49.4, 49.3, 49.2, 47.8, 46.3, 46.0, 45.8, 45.5, 45.3, 45.0, 44.6, 44.1, 43.6, 43.3, 43.1, 42.9, 41.5, 41.1, 28.2, 27.8, 27.7, 19.3, 19.2, 17.9, 17.8, 14.4, 14.3, 12.7, 12.5; LC-MS = 3.19 min; ESI MS *m/z* 553 $[M+H]^+$.

7{*2,3,3*}. As described using **6**{*3*} (129 mg, 0.250 mmol) and **5**{*2,3*} (158 mg, 0.549 mmol) in 4 mL of CH_2Cl_2 at rt. The reaction was stirred for 19 h. Column chromatography using a gradient of EtOAc/Hex (1:1 → 1:0) followed by EtOAc/MeOH (1:19 → 4:1) yielded 177 mg (64%) of a white solid. TLC R_f (EtOAc) = 0.46; ^{1}H-NMR ($CDCl_3$) δ 12.62 (t, 1H), 10.61–10.86 (m, 1H), 7.50 (d, J = 7.6 Hz, 1H), 5.61 (d, 1H), 4.43 (m, 2H), 3.19–3.86 (m, 8H), 1.37 (s, 9H), 1.30 (s, 9H), 1.19 (m, 12H); ^{13}C-NMR ($CDCl_3$) δ 173.2, 173.0, 171.3, 171.2, 165.7, 165.5, 165.0, 164.9, 159.1, 159.0, 158.9, 155.0, 135.1, 134.9, 134.6, 134.5, 134.3, 129.5, 129.2, 128.8, 81.3, 81.2, 79.2, 79.0, 49.3, 49.2, 47.8, 46.4, 46.3, 46.0, 45.8, 45.5, 45.3, 45.0, 44.6, 44.2, 43.6, 43.3, 43.1, 42.8, 41.6, 41.1, 29.4, 28.1, 27.8, 19.2, 19.1, 18.9, 17.8, 17.7, 14.4, 14.3, 14.2, 12.6, 12.5; LC-MS = 3.17 min; ESI MS *m/z* 553 $[M+H]^+$.

7{*2,3,4*}. No product isolated.

7{*2,4,1*}. As described using **6**{*1*} (122 mg, 0.242 mmol) and **5**{*2,4*} (175 mg, 0.532 mmol) in 4 mL of CH_2Cl_2 at rt. The reaction was stirred for 19 h. Column chromatography using a gradient of EtOAc/Hex (1:1 → 1:0) followed by EtOAc/MeOH (1:19 → 4:1) yielded 143 mg (51%) of a white solid. TLC R_f (EtOAc) = 0.59; ^{1}H-NMR ($CDCl_3$) δ 12.55 (m, 1H), 10.62–10.94 (m, 1H), 7.52 (d, J = 6.4 Hz, 1H), 5.39 (m,1H), 4.52 (m, 1H), 4.03 (m, 2H), 3.16–3.89 (m, 8H), 1.4 7–1.65 (m, 3H), 1.40 (s, 9H), 1.32 (s, 9H), 1.21 (m, 6H), 0.86 (m, 6H); ^{13}C-NMR ($CDCl_3$) δ 173.4, 173.3, 173.1, 172.8, 168.2, 168.1, 165.6, 165.5, 165.0, 164.8, 159.8, 155.5, 135.0, 134.9, 134.7, 134.6, 134.4, 129.4, 129.3, 129.0, 128.7, 81.7, 81.6, 79.2, 79.1, 79.0, 48.6, 48.0, 46.4, 45.8, 45.5, 45.4, 45.0, 44.7, 44.2, 43.7, 43.3, 43.1, 42.9, 42.7, 42.4, 41.7, 40.9, 28.2, 28.0, 27.9, 24.5, 23.3, 21.8, 21.6, 14.5, 14.4, 12.7, 12.6; LC-MS = 5.12 min; ESI MS *m/z* 581 $[M+H]^+$.

7{*2,4,2*}. As described using **6**{*2*} (125 mg, 0.242 mmol) and **5**{*2,4*} (175 mg, 0.532 mmol) in 4 mL of CH_2Cl_2 at rt. The reaction was stirred for 19 h. Column chromatography using a gradient of EtOAc/Hex (1:1 → 1:0) followed by EtOAc/MeOH (1:19 → 4:1) yielded 53 mg (18%) of a white solid. TLC R_f (EtOAc) = 0.61; ^{1}H-NMR ($CDCl_3$) δ 12.31 (m, 1H), 10.70–10.92 (m, 1H), 7.55 (d, *J* = 6.8 Hz, 1H), 5.27 (m,1H), 4.49 (m, 2H), 3.21–3.92 (m, 8H), 1.47–1.69 (m, 3H), 1.44 (s, 9H), 1.37 (s, 9H), 1.23 (m, 9H), 0.86 (m, 6H); ^{13}C-NMR ($CDCl_3$) δ 173.4, 173.3, 173.1, 172.9, 171.4, 171.3, 165.6, 165.5, 165.1, 164.9, 159.2, 159.2, 159.1, 155.6, 135.1, 135.0, 134.8, 134.6, 134.6, 134.5, 134.3, 129.6, 129.4, 129.3, 129.0, 81.5, 81.4, 79.4, 79.3, 79.2, 49.5, 49.4, 48.6, 48.1, 46.5, 45.9, 45.7, 45.5, 45.2, 44.8, 44.4, 43.8, 43.4, 43.1, 42.9, 42.7, 41.8, 41.0, 28.3, 28.0, 24.6, 23.4, 21.9, 21.7, 18.0, 17.9, 17.8, 14.5, 14.4, 12.8, 12.7; LC-MS = 5.78 min; ESI MS *m*/*z* 595 $[M+H]^+$.

7{*2,4,3*}. As described using **6**{*3*} (125 mg, 0.242 mmol) and **5**{*2,4*} (175 mg, 0.532 mmol) in 4 mL of CH_2Cl_2 at rt. The reaction was stirred for 19 h. Column chromatography using a gradient of EtOAc/Hex (1:1 → 1:0) followed by EtOAc/MeOH (1:19 → 4:1) yielded 169 mg (58%) of a white solid. TLC R_f (EtOAc) = 0.54; ^{1}H-NMR ($CDCl_3$) δ 12.64 (m, 1H), 10.62–10.86 (q, *J* = 6.8 Hz, 1H), 7.55 (d, *J* = 9.6 Hz, 1H), 5.40 (m,1H), 4.46 (m, 2H), 3.34–3.81 (m, 8H), 1.40–1.65 (m, 3H), 1.38 (s, 9H), 1.30 (s, 9H), 1.16 (m, 9H), 0.85 (m, 6H); ^{13}C-NMR ($CDCl_3$) δ 173.4, 173.3, 173.0, 172.9, 171.3, 171.2, 165.6, 165.5, 165.1, 164.9, 159.1, 159.0, 155.5, 135.0, 134.9, 134.7, 134.6, 134.4, 129.4, 129.2, 129.0, 128.8, 81.3, 81.2, 79.1, 79.0, 49.4, 49.2, 48.5, 48.0, 46.4, 45.8, 45.5, 45.3, 45.0, 44.6, 44.1, 43.7, 43.3, 43.2, 42.9, 42.6, 41.6, 40.9, 28.1, 27.9, 27.7, 24.5, 23.2, 21.8, 21.7, 21.5, 17.8, 17.7, 14.4, 14.3, 12.7, 12.6, 12.5; LC-MS = 5.81 min; ESI MS *m*/*z* 595 $[M+H]^+$.

7{*2,4,4*}. As described using **6**{*4*} (135 mg, 0.242 mmol) and **5**{*2,4*} (175 mg, 0.532 mmol) in 4 mL of CH_2Cl_2 at rt. The reaction was stirred for 19 h. Column chromatography using a gradient of EtOAc/Hex (1:1 → 1:0) followed by EtOAc/MeOH (1:19 → 4:1) yielded 142 mg (46%) of a white solid. TLC R_f (EtOAc) = 0.61; ^{1}H-NMsR ($CDCl_3$) δ 12.64 (m, 1H), 10.67–10.86 (m, 1H), 7.52 (d, *J* = 7.2 Hz, 1H), 5.37 (m,1H), 4.47 (m, 2H), 3.36–3.88 (m, 8H), 1.4 6–1.76 (m, 6H), 1.39 (s, 9H), 1.33 (s, 9H), 1.18 (m, 6H), 0.87 (m, 12H); ^{13}C-NMR ($CDCl_3$) δ 173.4, 173.3, 173.1, 172.9, 171.3, 171.2, 165.6, 165.1, 164.9, 159.4, 159.3, 159.2, 155.6, 155.5, 135.0, 134.7, 134.4, 129.5, 129.3, 129.2, 128.9, 81.3, 81.2, 79.2, 79.1, 52.3, 52.2, 48.6, 47.9, 46.6, 46.4, 45.9, 45.6, 45.5, 45.2, 44.8, 44.4, 43.8, 43.4, 43.2, 42.9, 42.7, 41.8, 41.1, 41.0, 40.9, 29.5, 28.2, 28.1, 27.9, 24.9, 24.6, 23.3, 22.8, 22.7, 21.9, 21.8, 21.6, 14.5, 14.4, 12.8, 12.7, 12.5; LC-MS = 7.56 min; ESI MS *m*/*z* 637 $[M+H]^+$.

8{*1-2,1-4,1-4*}. To a solution of one intermediate, **7**{*1-2,1-4,1-4*} in CH_2Cl_2 was added trifluoroacetic acid at rt. The reaction was stirred until complete and the solvent removed under vacuum. The residue was taken up in aqueous HCl and lyophilized to dryness, yielding a material that was used without further purification.

8{*1,1,1*}. As described using **7**{*1,1,1*} (210 mg, 0.423 mmol), 5 mL of CH_2Cl_2 and 1 mL of TFA. The reaction was stirred for 13 h. LC-MS = 1.14 min; ESI MS *m*/*z* 341 $[M+H]^+$.

8{*1,1,2*}. As described using **7**{*1,1,2*} (275 mg, 0.539 mmol), 5 mL of CH_2Cl_2 and 1 mL of TFA. The reaction was stirred for 13 h. LC-MS = 1.14 min; ESI MS *m*/*z* 355 $[M+H]^+$.

8{*1,1,3*}. As described using **7**{*1,1,3*} (260 mg, 0.510 mmol), 5 mL of CH_2Cl_2 and 1 mL of TFA. The reaction was stirred for 13 h. LC-MS = 1.14 min; ESI MS *m*/*z* 355 $[M+H]^+$.

8{*1,1,4*}. As described using **7**{*1,1,4*} (238 mg, 0.431 mmol), 5 mL of CH_2Cl_2 and 1 mL of TFA. The reaction was stirred for 13.5 h. LC-MS = 1.09 min; ESI MS *m*/*z* 397 $[M+H]^+$.

8{*1,2,1*}. As described using **7**{*1,2,1*} (129 mg, 0.253 mmol), 2 mL of CH_2Cl_2 and 0.5 mL of TFA. The reaction was stirred for 17.5 h. LC-MS = 1.12 min; ESI MS *m*/*z* 355 $[M+H]^+$.

8{*1,2,2*}. As described using **7**{*1,2,2*} (295 mg, 0.563 mmol), 10 mL of CH_2Cl_2 and 1.5 mL of TFA. The reaction was stirred for 10.5 h. LC-MS = 1.08 min; ESI MS *m*/*z* 369 $[M+H]^+$.

8{*1,2,3*}. As described using **7**{*1,2,3*} (136 mg, 0.260 mmol), 2 mL of CH_2Cl_2 and 0.5 mL of TFA. The reaction was stirred for 17.5 h. LC-MS = 1.10 min; ESI MS *m*/*z* 369 $[M+H]^+$.

8{*1,2,4*}. As described using **7**{*1,2,4*} (140 mg, 0.247 mmol), 2 mL of CH_2Cl_2 and 0.5 mL of TFA. The reaction was stirred for 17.5 h. LC-MS = 1.10 min; ESI MS *m*/*z* 411 $[M+H]^+$.

8{*1,3,1*}. As described using **7**{*1,3,1*} (57 mg, 0.112 mmol), 2 mL of CH_2Cl_2 and 0.5 mL of TFA. The reaction was stirred for 17.5 h. LC-MS = 1.28 min; ESI MS *m*/*z* 355 $[M+H]^+$.

8{*1,3,2*}. As described using **7**{*1,3,2*} (47 mg, 0.090 mmol), 2 mL of CH_2Cl_2 and 0.5 mL of TFA. The reaction was stirred for 17.5 h. LC-MS = 1.09 min; ESI MS *m*/*z* 369 $[M+H]^+$.

8{*1,3,3*}. As described using **7**{*1,3,3*} (42 mg, 0.080 mmol), 2 mL of CH_2Cl_2 and 0.5 mL of TFA. The reaction was stirred for 17.5 h. LC-MS = 1.12 min; ESI MS *m*/*z* 369 $[M+H]^+$.

8{*1,3,4*}. As described using **7**{*1,3,4*} (51 mg, 0.090 mmol), 2 mL of CH_2Cl_2 and 0.5 mL of TFA. The reaction was stirred for 17.5 h. LC-MS = 1.13 min; ESI MS *m*/*z* 411 $[M+H]^+$.

8{*1,4,1*}. As described using **7**{*1,4,1*} (59 mg, 0.107 mmol), 2 mL of CH_2Cl_2 and 0.5 mL of TFA. The reaction was stirred for 17.5 h. LC-MS = 1.31 min; ESI MS *m*/*z* 397 $[M+H]^+$.

8{*1,4,2*}. As described using **7**{*1,4,2*} (69 mg, 0.122 mmol), 2 mL of CH_2Cl_2 and 0.5 mL of TFA. The reaction was stirred for 17.5 h. LC-MS = 1.11 min; ESI MS *m*/*z* 411 $[M+H]^+$.

8{*1,4,3*}. As described using **7**{*1,4,3*} (49 mg, 0.087 mmol), 2 mL of CH_2Cl_2 and 0.5 mL of TFA. The reaction was stirred for 17.5 h. LC-MS = 1.10 min; ESI MS *m*/*z* 411 $[M+H]^+$.

8{*1,4,4*}. No starting material available.

8{*2,1,1*}. As described using **7**{*2,1,1*} (146 mg, 0.279 mmol), 2 mL of CH_2Cl_2 and 0.5 mL of TFA. The reaction was stirred for 17.5 h. LC-MS = 1.11 min; ESI MS *m*/*z* 369 $[M+H]^+$.

8{*2,1,2*}. As described using **7**{*2,1,2*} (140 mg, 0.260 mmol), 2 mL of CH_2Cl_2 and 0.5 mL of TFA. The reaction was stirred for 17.5 h. LC-MS = 1.08 min; ESI MS *m*/*z* 383 $[M+H]^+$.

8{*2,1,3*}. As described using **7**{*2,1,3*} (170 mg, 0.316 mmol), 2 mL of CH_2Cl_2 and 0.5 mL of TFA. The reaction was stirred for 17.5 h. LC-MS = 1.10 min; ESI MS *m/z* 383 $[M+H]^+$.

8{*2,1,4*}. No starting material available.

8{*2,2,1*}. As described using **7**{*2,2,1*} (135 mg, 0.251 mmol), 2 mL of CH_2Cl_2 and 0.5 mL of TFA. The reaction was stirred for 17.5 h. LC-MS = 1.09 min; ESI MS *m/z* 383 $[M+H]^+$.

8{*2,2,2*}. As described using **7**{*2,2,2*} (140 mg, 0.254 mmol), 2 mL of CH_2Cl_2 and 0.5 mL of TFA. The reaction was stirred for 17.5 h. LC-MS = 1.10 min; ESI MS *m/z* 397 $[M+H]^+$.

8{*2,2,3*}. As described using **7**{*2,2,3*} (163 mg, 0.295 mmol), 2 mL of CH_2Cl_2 and 0.5 mL of TFA. The reaction was stirred for 17.5 h. LC-MS = 1.09 min; ESI MS *m/z* 397 $[M+H]^+$.

8{*2,2,4*}. No starting material available.

8{*2,3,1*}. As described using **7**{*2,3,1*} (138 mg, 0.257 mmol), 2 mL of CH_2Cl_2 and 0.5 mL of TFA. The reaction was stirred for 17.5 h. LC-MS = 1.05 min; ESI MS *m/z* 383 $[M+H]^+$.

8{*2,3,2*}. As described using **7**{*2,3,2*} (150 mg, 0.272 mmol), 2 mL of CH_2Cl_2 and 0.5 mL of TFA. The reaction was stirred for 17.5 h. LC-MS = 1.13 min; ESI MS *m/z* 397 $[M+H]^+$.

8{*2,3,3*}. As described using **7**{*2,3,3*} (177 mg, 0.321 mmol), 2 mL of CH_2Cl_2 and 0.5 mL of TFA. The reaction was stirred for 17.5 h. LC-MS = 1.08 min; ESI MS *m/z* 397 $[M+H]^+$.

8{*2,3,4*}. No starting material available.

8{*2,4,1*}. As described using **7**{*2,4,1*} (143 mg, 0.247 mmol), 2 mL of CH_2Cl_2 and 0.5 mL of TFA. The reaction was stirred for 17.5 h. LC-MS = 1.10 min; ESI MS *m/z* 425 $[M+H]^+$.

8{*2,4,2*}. As described using **7**{*2,4,2*} (53 mg, 0.089 mmol), 2 mL of CH_2Cl_2 and 0.5 mL of TFA. The reaction was stirred for 17.5 h. LC-MS = 1.12 min; ESI MS *m/z* 439 $[M+H]^+$.

8{*2,4,3*}. As described using **7**{*2,4,3*} (169 mg, 0.285 mmol), 2 mL of CH_2Cl_2 and 0.5 mL of TFA. The reaction was stirred for 17.5 h. LC-MS = 1.08 min; ESI MS *m/z* 439 $[M+H]^+$.

8{*2,4,4*}. As described using **7**{*2,4,4*} (142 mg, 0.223 mmol), 2 mL of CH_2Cl_2 and 0.5 mL of TFA. The reaction was stirred for 17.5 h. LC-MS = 1.08 min; ESI MS *m/z* 481 $[M+H]^+$.

9{*1-2,1-4,1-4*}. To 20 mL of CH_2Cl_2 in a dry roundbottom flask was added dropwise over 1 h a solution of one intermediate, **8**{*1-2,1-4,1-4*} (1 equiv.) in 20 mL of CH_2Cl_2 concurrent with the dropwise addition of a solution containing EDC•HCl (2.2 equiv.) and DMAP (0.2 equiv.) in 20 mL of CH_2Cl_2. The resulting solution was stirred for a period of time before removing the solvent under vacuum and purifying the macrocycle by column chromatography on silica gel.

9{*1,1,1*}. As described using **8**{*1,1,1*} (72 mg, 0.210 mmol), EDC•HCl (89 mg, 0.466 mmol), and DMAP (5 mg, 0.042 mmol). The reaction was stirred for 10.5 h. Column chromatography using a gradient of EtOAc/Hex (1:1 → 1:0) followed by EtOAc/MeOH (1:19 → 3:2) yielded 32 mg (47%) of a white solid. TLC R*f* (EtOAc/MeOH 1:1) = 0.25; LC-MS = 1.19 min; ESI MS *m*/*z* 323 $[M+H]^+$.

9{*1,1,2*}. As described using **8**{*1,1,2*} (95 mg, 0.270 mmol), EDC•HCl (113 mg, 0.593 mmol), and DMAP (7 mg, 0.054 mmol). The reaction was stirred for 21 h. Column chromatography using a gradient of EtOAc/Hex (1:1 → 1:0) followed by EtOAc/MeOH (1:19 → 3:2) yielded 32 mg (47%) of a white solid. TLC R*f* (EtOAc/MeOH 1:1) = 0.35; LC-MS = 1.24 min; ESI MS *m*/*z* 337 $[M+H]^+$.

9{*1,1,3*}. As described using **8**{*1,1,3*} (90 mg, 0.254 mmol), EDC•HCl (107 mg, 0.559 mmol), and DMAP (6 mg, 0.051 mmol). The reaction was stirred for 18 h. Column chromatography using a gradient of EtOAc/Hex (1:1 → 1:0) followed by EtOAc/MeOH (1:19 → 3:2) yielded 32 mg (47%) of a white solid. TLC R*f* (EtOAc/MeOH 1:1) = 0.30; LC-MS = 1.24 min; ESI MS *m*/*z* 337 $[M+H]^+$.

9{*1,1,4*}. As described using **8**{*1,1,4*} (85 mg, 0.216 mmol), EDC•HCl (91 mg, 0.474 mmol), and DMAP (5 mg, 0.043 mmol). The reaction was stirred for 17.5 h. Column chromatography using a gradient of EtOAc/Hex (1:1 → 1:0) followed by EtOAc/MeOH (1:19 → 3:2) yielded 32 mg (47%) of a white solid. TLC R*f* (EtOAc/MeOH 1:1) = 0.40; LC-MS = 1.43 min; ESI MS *m*/*z* 379 $[M+H]^+$.

9{*1,2,1*}. As described using **8**{*1,2,1*} (90 mg, 0.253 mmol), EDC•HCl (107 mg, 0.557 mmol), and DMAP (6 mg, 0.051 mmol). The reaction was stirred for 14 h. Column chromatography using a gradient of EtOAc/Hex (1:1 → 1:0) followed by EtOAc/MeOH (1:19 → 3:2) yielded no obvious fractions with the desired product as determined by LC-MS analysis.

9{*1,2,2*}. As described using **8**{*1,2,2*} (227 mg, 0.563 mmol), EDC•HCl (192 mg, 1.24 mmol), and DMAP (14 mg, 0.113 mmol). The reaction was stirred for 14 h. Column chromatography using a gradient of EtOAc/Hex (1:1 → 1:0) followed by EtOAc/MeOH (1:19 → 3:2) yielded 11 mg (32%). LC-MS = 1.19 min; ESI MS *m*/*z* 351 $[M+H]^+$.

9{*1,2,3*}. As described using **8**{*1,2,3*} (105 mg, 0.260 mmol), EDC•HCl (89 mg, 0.572 mmol), and DMAP (6 mg, 0.052 mmol). The reaction was stirred for 14 h. Column chromatography using a gradient of EtOAc/Hex (1:1 → 1:0) followed by EtOAc/MeOH (1:19 → 3:2) yielded no obvious fractions with the desired product as determined by LC-MS analysis.

9{*1,2,4*}. As described using **8**{*1,2,4*} (110 mg, 0.247 mmol), EDC•HCl (84 mg, 0.543 mmol), and DMAP (6 mg, 0.049 mmol). The reaction was stirred for 14 h. Column chromatography using a gradient of EtOAc/Hex (1:1 → 1:0) followed by EtOAc/MeOH (1:19 → 3:2) yielded 11 mg (12%). LC-MS = 1.47 min; ESI MS *m*/*z* 393 $[M+H]^+$.

9{*1,3,1*}. As described using **8**{*1,3,1*} (44 mg, 0.112 mmol), EDC•HCl (38 mg, 0.246 mmol), and DMAP (2 mg, 0.018 mmol). The reaction was stirred for 14 h. Column chromatography using a gradient of EtOAc/Hex (1:1 → 1:0) followed by EtOAc/MeOH (1:19 → 3:2) yielded no obvious fractions with the desired product as determined by LC-MS analysis.

9{*1,3,2*}. As described using **8**{*1,3,2*} (36 mg, 0.090 mmol), EDC•HCl (31 mg, 0.198 mmol), and DMAP (2 mg, 0.018 mmol). The reaction was stirred for 14 h. Column chromatography using a gradient of EtOAc/Hex (1:1 → 1:0) followed by EtOAc/MeOH (1:19 → 3:2) yielded 7 mg (22%). LC-MS = 1.56 min; ESI MS *m*/*z* 351 $[M+H]^+$.

9{*1,3,3*}. As described using **8**{*1,3,3*} (32 mg, 0.080 mmol), EDC•HCl (27 mg, 0.176 mmol), and DMAP (2 mg, 0.016 mmol). The reaction was stirred for 14 h. Column chromatography using a gradient of EtOAc/Hex (1:1 → 1:0) followed by EtOAc/MeOH (1:19 → 3:2) yielded no obvious fractions with the desired product as determined by LC-MS analysis.

9{*1,3,4*}. As described using **8**{*1,3,4*} (40 mg, 0.090 mmol), EDC•HCl (31 mg, 0.198 mmol), and DMAP (2 mg, 0.018 mmol). The reaction was stirred for 14 h. Column chromatography using a gradient of EtOAc/Hex (1:1 → 1:0) followed by EtOAc/MeOH (1:19 → 3:2) yielded 4 mg (11%). LC-MS = 1.43 min; ESI MS *m*/*z* 393 $[M+H]^+$.

9{*1,4,1*}. As described using **8**{*1,4,1*} (46 mg, 0.107 mmol), EDC•HCl (37 mg, 0.235 mmol), and DMAP (3 mg, 0.021 mmol). The reaction was stirred for 14 h. Column chromatography using a gradient of EtOAc/Hex (1:1 → 1:0) followed by EtOAc/MeOH (1:19 → 3:2) yielded no obvious fractions with the desired product as determined by LC-MS analysis.

9{*1,4,2*}. As described using **8**{*1,4,2*} (55 mg, 0.122 mmol), EDC•HCl (42 mg, 0.268 mmol), and DMAP (3 mg, 0.024 mmol). The reaction was stirred for 14 h. Column chromatography using a gradient of EtOAc/Hex (1:1 → 1:0) followed by EtOAc/MeOH (1:19 → 3:2) yielded 7 mg (14%). LC-MS = 1.43 min; ESI MS *m*/*z* 393 $[M+H]^+$.

9{*1,4,3*}. As described using **8**{*1,4,3*} (39 mg, 0.087 mmol), EDC•HCl (30 mg, 0.191 mmol), and DMAP (2 mg, 0.017 mmol). The reaction was stirred for 14 h. Column chromatography using a gradient of EtOAc/Hex (1:1 → 1:0) followed by EtOAc/MeOH (1:19 → 3:2) yielded 13 mg (37%). LC-MS = 1.43 min; ESI MS *m*/*z* 393 $[M+H]^+$.

9{*1,4,4*}. No starting material available.

9{*2,1,1*}. As described using **8**{*2,1,1*} (113 mg, 0.279 mmol), EDC•HCl (95 mg, 0.614 mmol), and DMAP(7 mg, 0.056 mmol). The reaction was stirred for 14 h. Column chromatography using a gradient of EtOAc/Hex (1:1 → 1:0) followed by EtOAc/MeOH (1:19 → 3:2) yielded no obvious fractions with the desired product as determined by LC-MS analysis.

9{*2,1,2*}. As described using **8**{*2,1,2*} (109 mg, 0.260 mmol), EDC•HCl (89 mg, 0.572 mmol), and DMAP (6 mg, 0.052 mmol). The reaction was stirred for 14 h. Column chromatography using a gradient of EtOAc/Hex (1:1 → 1:0) followed by EtOAc/MeOH (1:19 → 3:2) yielded 9 mg (9%). LC-MS = 1.34 min; ESI MS *m*/*z* 365 $[M+H]^+$.

9{*2,1,3*}. As described using **8**{*2,1,3*} (132 mg, 0.316 mmol), EDC•HCl (108 mg, 0.695 mmol), and DMAP (8 mg, 0.063 mmol). The reaction was stirred for 14 h. Column chromatography using a

gradient of EtOAc/Hex (1:1 → 1:0) followed by EtOAc/MeOH (1:19 → 3:2) yielded 9 mg (8%). LC-MS = 1.39 min; ESI MS *m*/*z* 365 $[M+H]^+$.

9{*2,1,4*}. No starting material available.

9{*2,2,1*}. As described using **8**{*2,2,1*} (105 mg, 0.251 mmol), EDC•HCl (86 mg, 0.552 mmol), and DMAP (6 mg, 0.050 mmol). The reaction was stirred for 14 h. Column chromatography using a gradient of EtOAc/Hex (1:1 → 1:0) followed by EtOAc/MeOH (1:19 → 3:2) yielded no obvious fractions with the desired product as determined by LC-MS analysis.

9{*2,2,2*}. As described using **8**{*2,2,2*} (110 mg, 0.254 mmol), EDC•HCl (87 mg, 0.559 mmol), and DMAP (6 mg, 0.051 mmol). The reaction was stirred for 14 h. Column chromatography using a gradient of EtOAc/Hex (1:1 → 1:0) followed by EtOAc/MeOH (1:19 → 3:2) yielded 7 mg (7%). LC-MS = 1.43 min; ESI MS *m*/*z* 379 $[M+H]^+$.

9{*2,2,3*}. As described using **8**{*2,2,3*} (128 mg, 0.295 mmol), EDC•HCl (101 mg, 0.649 mmol), and DMAP (7 mg, 0.059 mmol). The reaction was stirred for 14 h. Column chromatography using a gradient of EtOAc/Hex (1:1 → 1:0) followed by EtOAc/MeOH (1:19 → 3:2) yielded 9 mg (8%). LC-MS = 1.38 min; ESI MS *m*/*z* 379 $[M+H]^+$.
9{*2,2,4*}. No starting material available.

9{*2,3,1*}. As described using **8**{*2,3,1*} (111 mg, 0.257 mmol), EDC•HCl (88 mg, 0.565 mmol), and DMAP (6 mg, 0.051 mmol). The reaction was stirred for 14 h. Column chromatography using a gradient of EtOAc/Hex (1:1 → 1:0) followed by EtOAc/MeOH (1:19 → 3:2) yielded 2 mg (3%). LC-MS = 1.39 min; ESI MS *m*/*z* 365 $[M+H]^+$.

9{*2,3,2*}. As described using **8**{*2,3,2*} (118 mg, 0.272 mmol), EDC•HCl (93 mg, 0.598 mmol), and DMAP (7 mg, 0.054 mmol). The reaction was stirred for 14 h. Column chromatography using a gradient of EtOAc/Hex (1:1 → 1:0) followed by EtOAc/MeOH (1:19 → 3:2) yielded 11 mg (10%). LC-MS = 1.47 min; ESI MS *m*/*z* 379 $[M+H]^+$.

9{*2,3,3*}. As described using **8**{*2,3,3*} (139 mg, 0.321 mmol), EDC•HCl (110 mg, 0.706 mmol), and DMAP (8 mg, 0.064 mmol). The reaction was stirred for 14 h. Column chromatography using a gradient of EtOAc/Hex (1:1 → 1:0) followed by EtOAc/MeOH (1:19 → 3:2) yielded 11 mg (9%). LC-MS = 1.49 min; ESI MS *m*/*z* 379 $[M+H]^+$.

9{*2,3,4*}. No starting material available.

9{*2,4,1*}. As described using **8**{*2,4,1*} (114 mg, 0.247 mmol), EDC•HCl (84 mg, 0.543 mmol), and DMAP (6 mg, 0.049 mmol). The reaction was stirred for 14 h. Column chromatography using a gradient of EtOAc/Hex (1:1 → 1:0) followed by EtOAc/MeOH (1:19 → 3:2) yielded 8 mg (8%). LC-MS = 1.51 min; ESI MS *m*/*z* 407 $[M+H]^+$.

9{*2,4,2*}. As described using **8**{*2,4,2*} (42 mg, 0.089 mmol), EDC•HCl (30 mg, 0.196 mmol), and DMAP (2 mg, 0.018 mmol). The reaction was stirred for 14 h. Column chromatography using a

gradient of EtOAc/Hex (1:1 → 1:0) followed by EtOAc/MeOH (1:19 → 3:2) yielded 4 mg (11%). LC-MS = 1.61 min; ESI MS *m*/*z* 421 [M+H]$^+$.

9{*2,4,3*}. As described using **8**{*2,4,3*} (135 mg, 0.285 mmol), EDC•HCl (97 mg, 0.627 mmol), and DMAP (7 mg, 0.057 mmol). The reaction was stirred for 14 h. Column chromatography using a gradient of EtOAc/Hex (1:1 → 1:0) followed by EtOAc/MeOH (1:19 → 3:2) yielded no obvious fractions with the desired product as determined by LC-MS analysis.

9{*2,4,4*}. As described using **8**{*2,4,4*} (115 mg, 0.223 mmol), EDC•HCl (76 mg, 0.491 mmol), and DMAP (5 mg, 0.045 mmol). The reaction was stirred for 14 h. Column chromatography using a gradient of EtOAc/Hex (1:1 → 1:0) followed by EtOAc/MeOH (1:19 → 3:2) yielded no obvious fractions with the desired product as determined by LC-MS analysis.

Figure S1. LC-MS of **9**{*1,1,1*}.

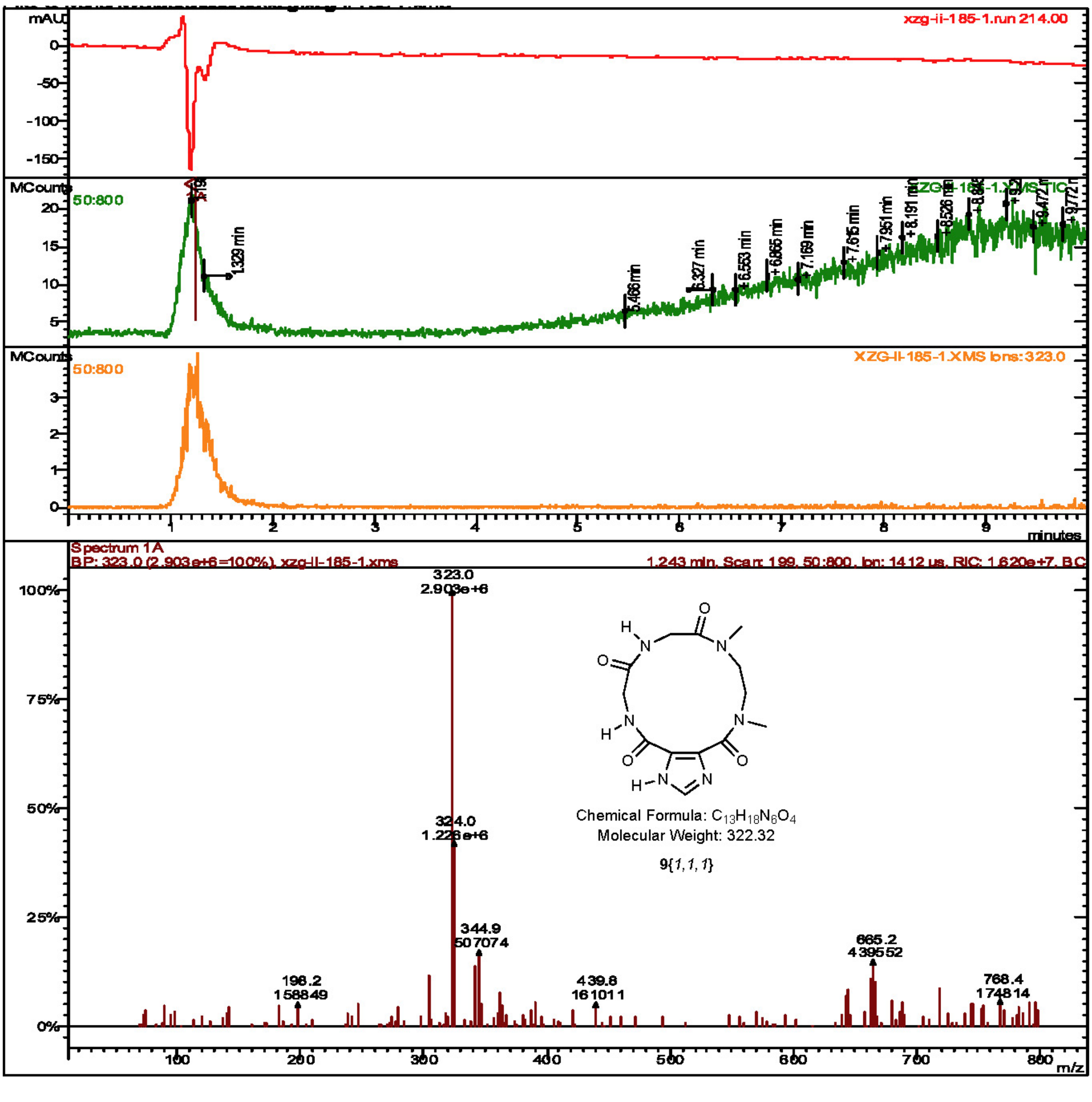

Figure S2. ^{1}H-NMR Spectra of **9**{*1,1,1*}.

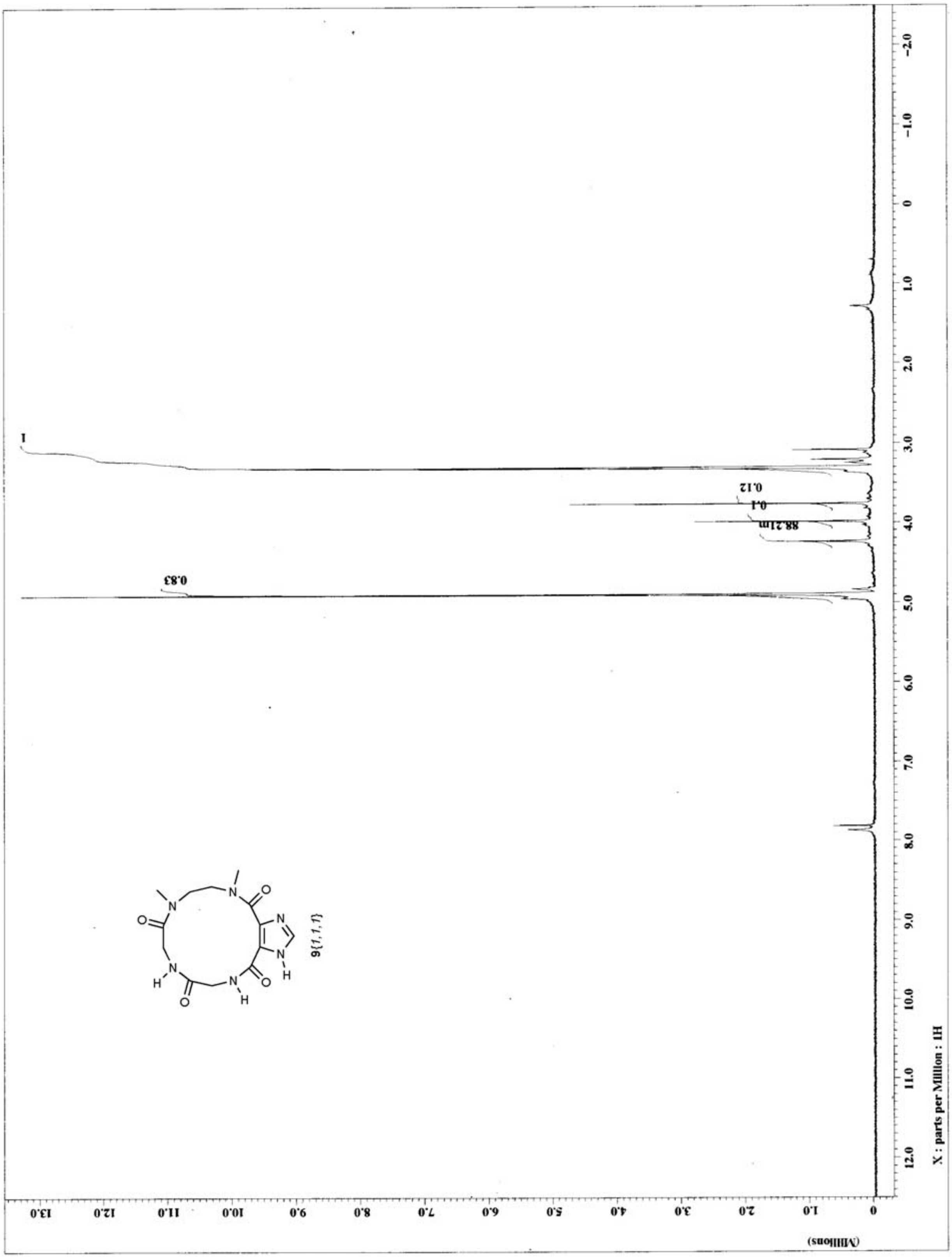

Figure S3. LC-MS of **9**{*1,1,2*}.

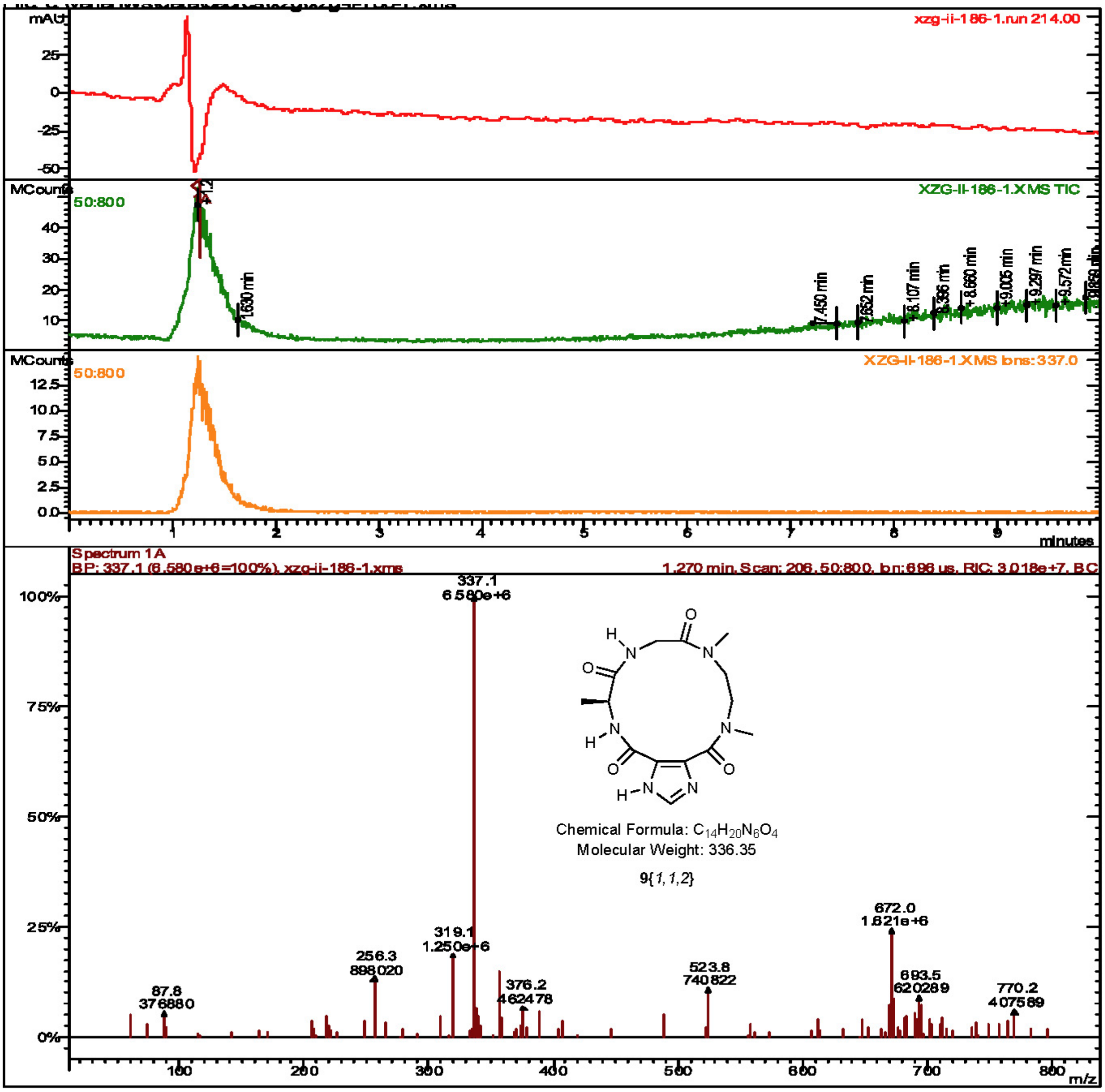

Figure S4. ^{1}H-NMR Spectra of **9**{*1,1,2*}.

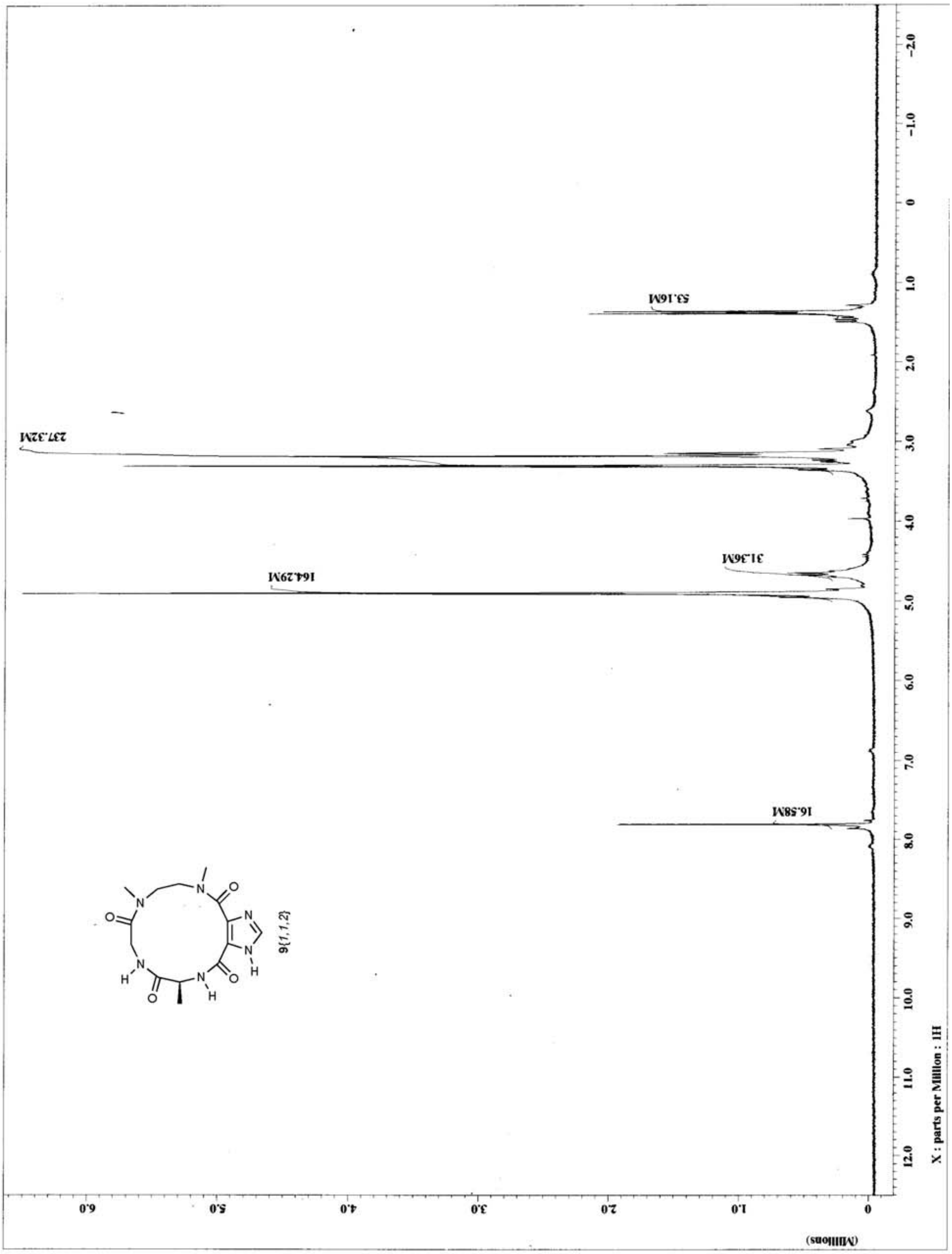

Figure S5. LC-MS of **9**{*1,1,3*}.

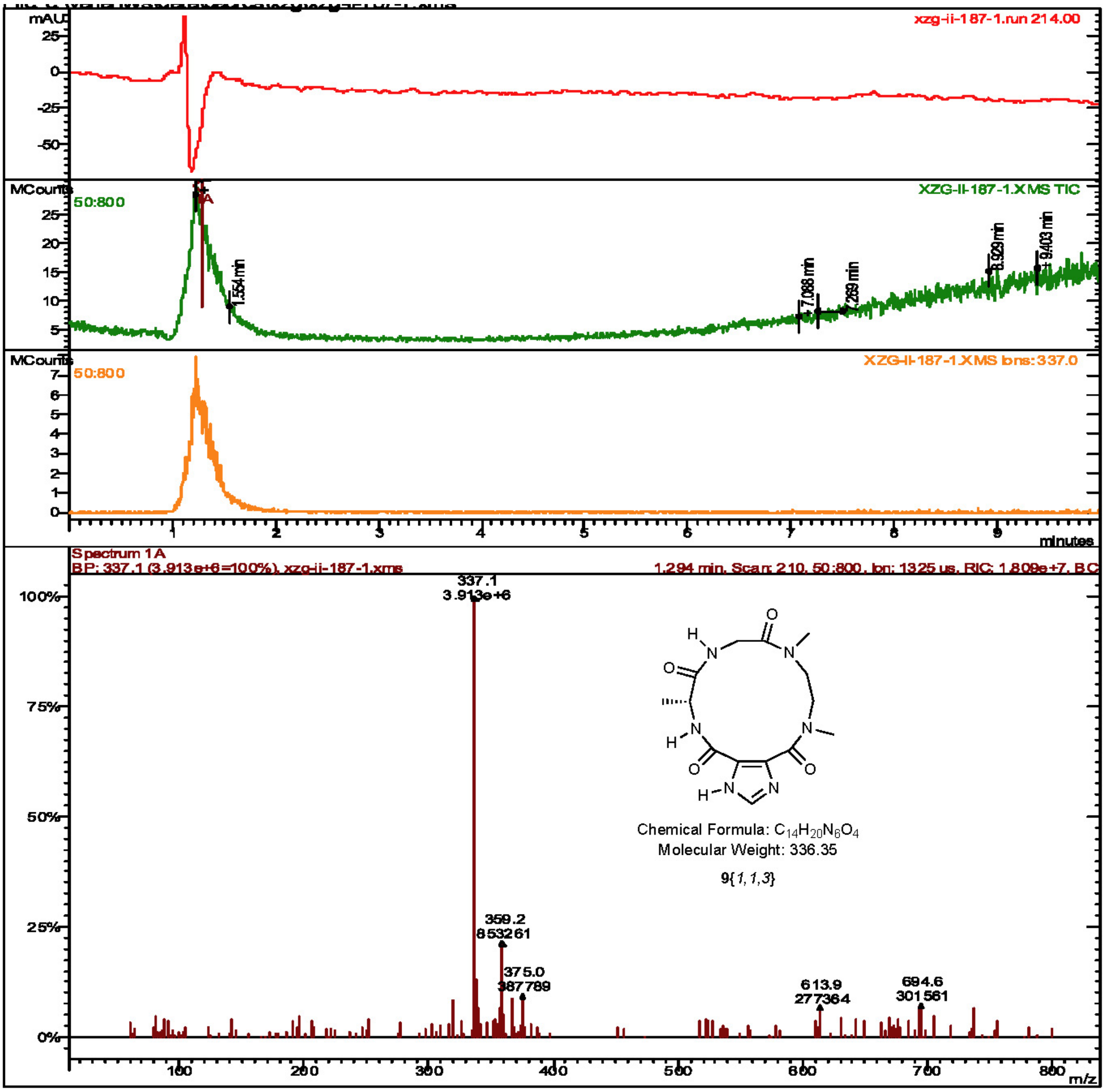

Figure S6. ^{1}H-NMR Spectra of **9**{*1,1,3*}.

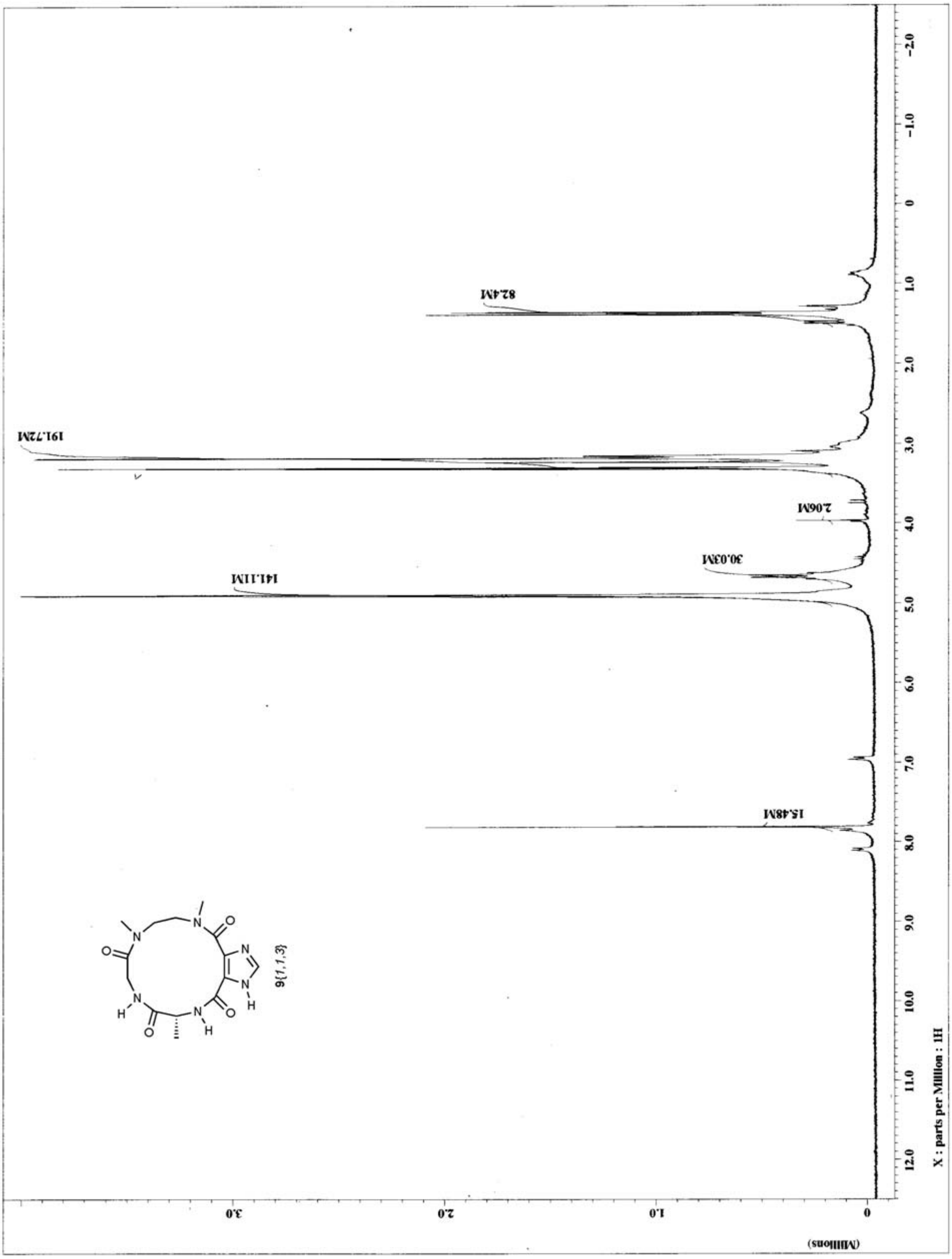

Figure S7. LC-MS of **9**{*1,1,4*}.

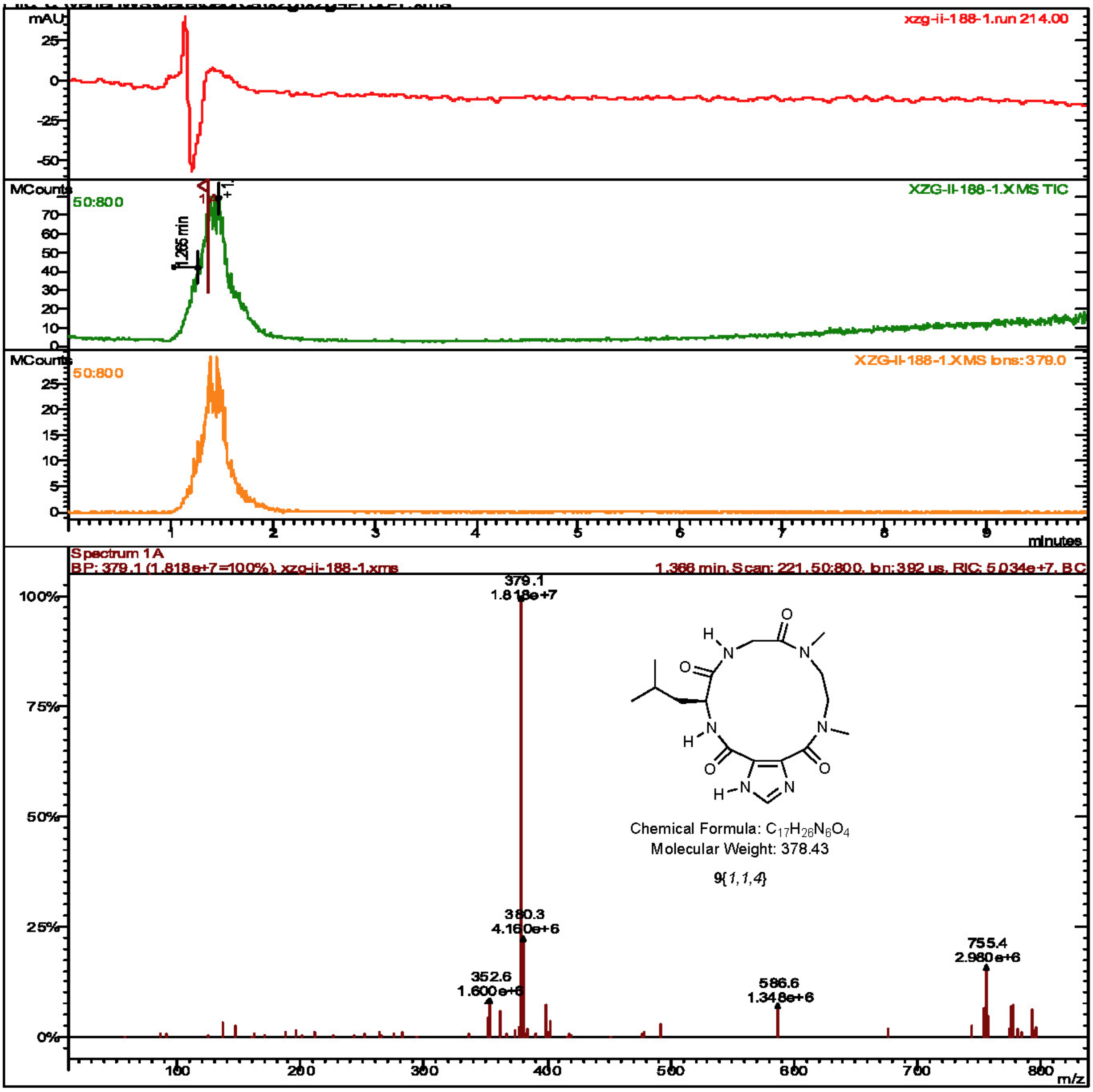

Figure S8. ^{1}H-NMR Spectra of **9**{*1,1,4*}.

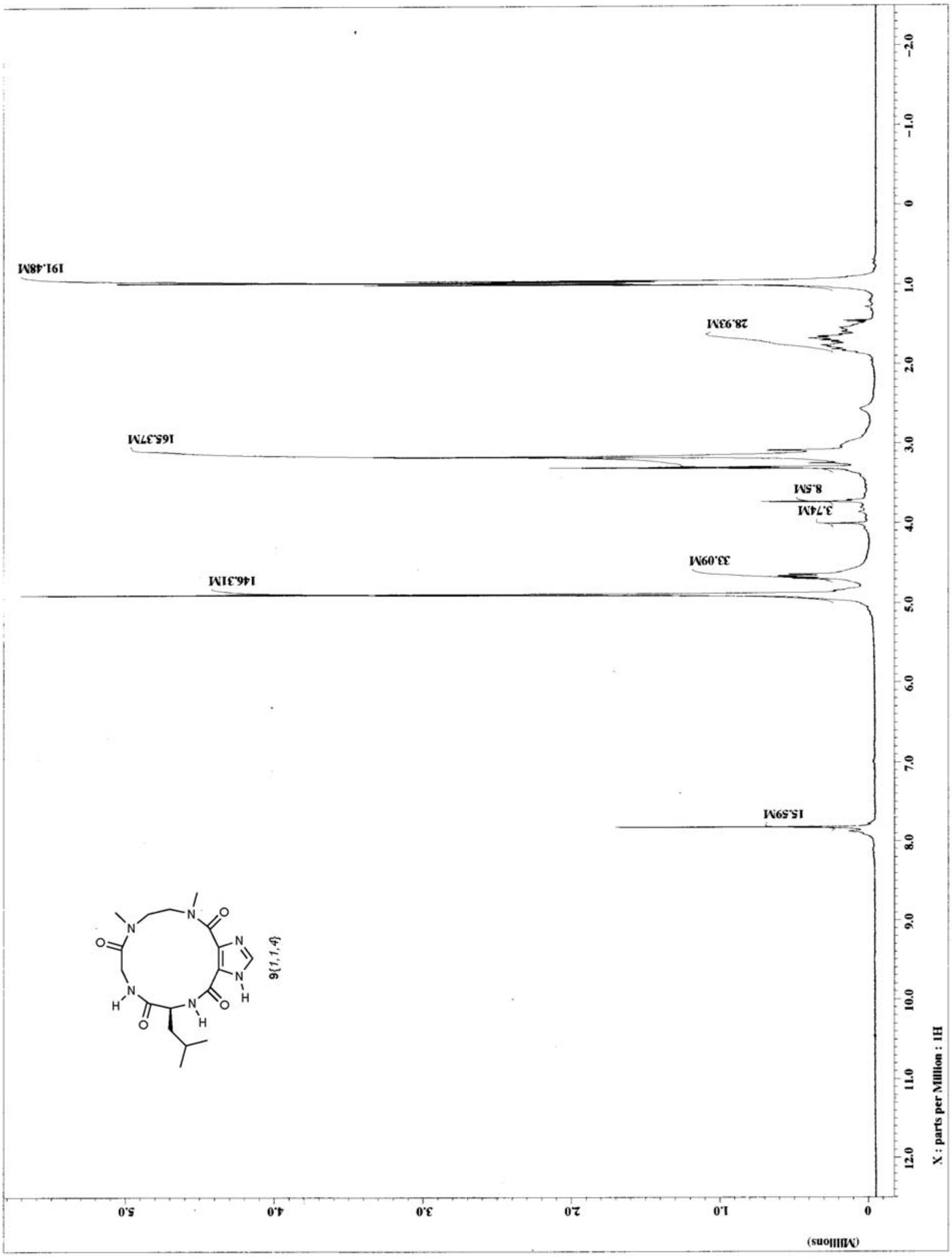

Figure S9. LC-MS of **9**{*1,2,2*}.

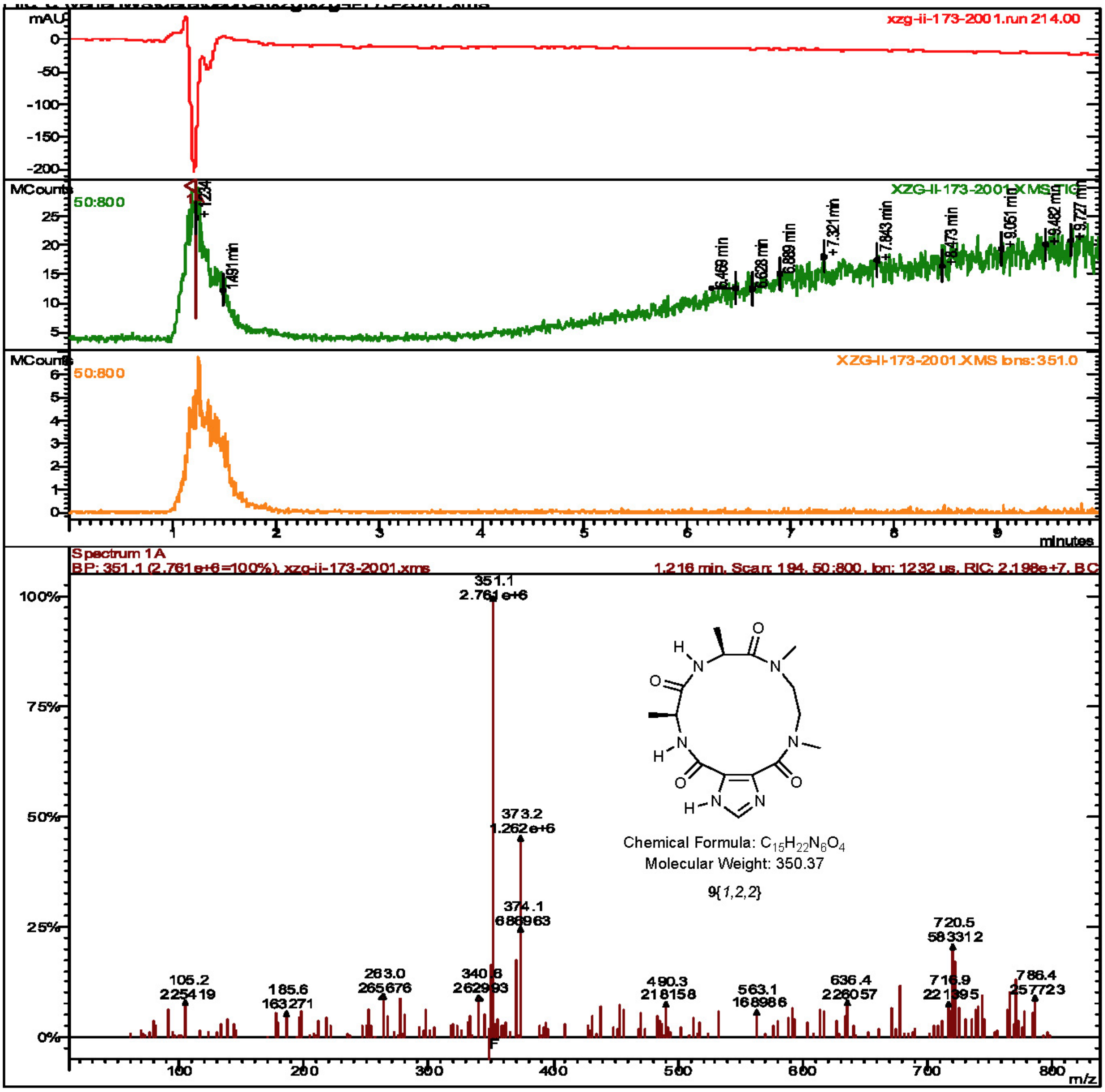

Figure S10. ^{1}H-NMR Spectra of **9**{*1,2,2*}.

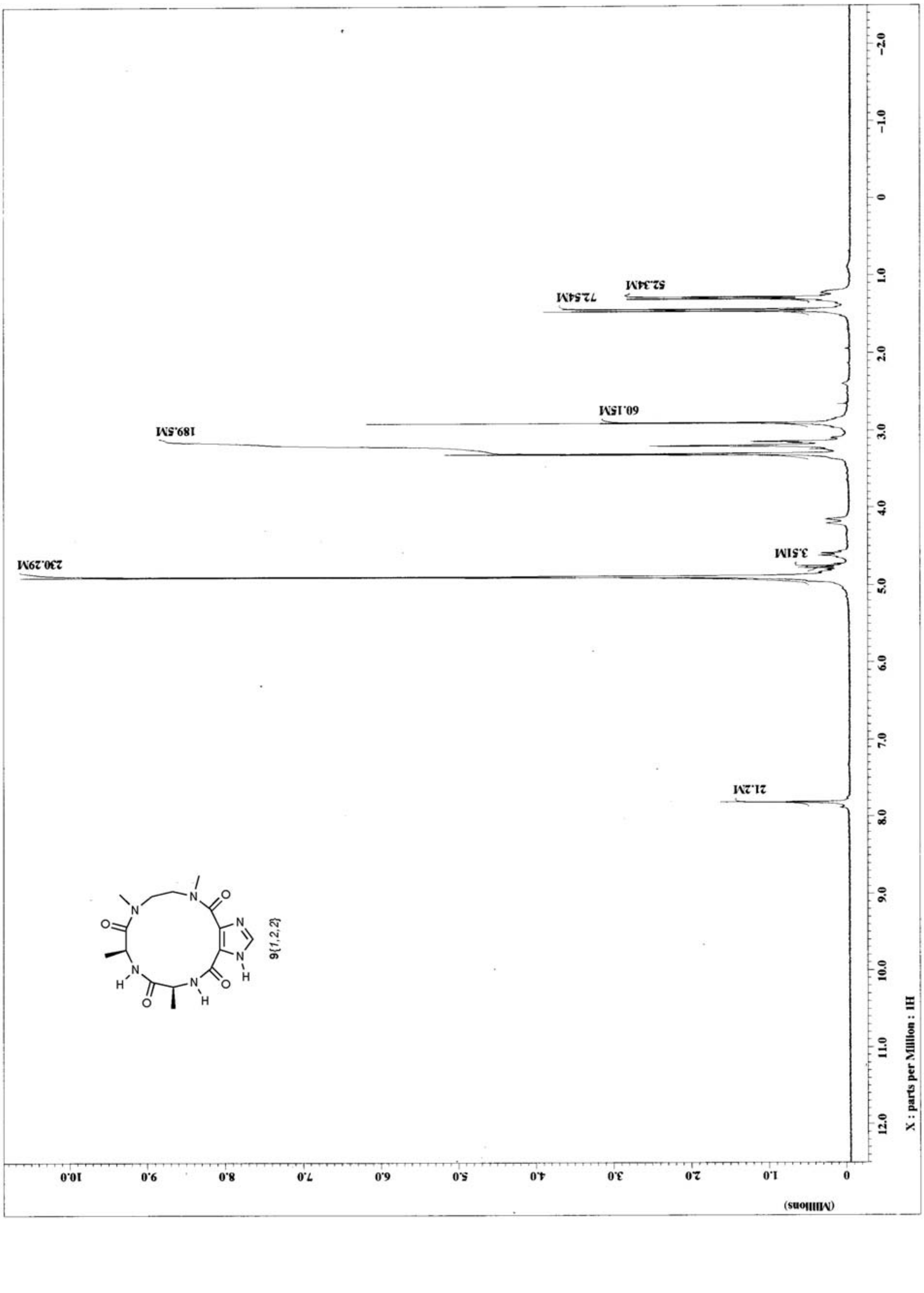

Figure S11. LC-MS of **9**{*1,2,4*}.

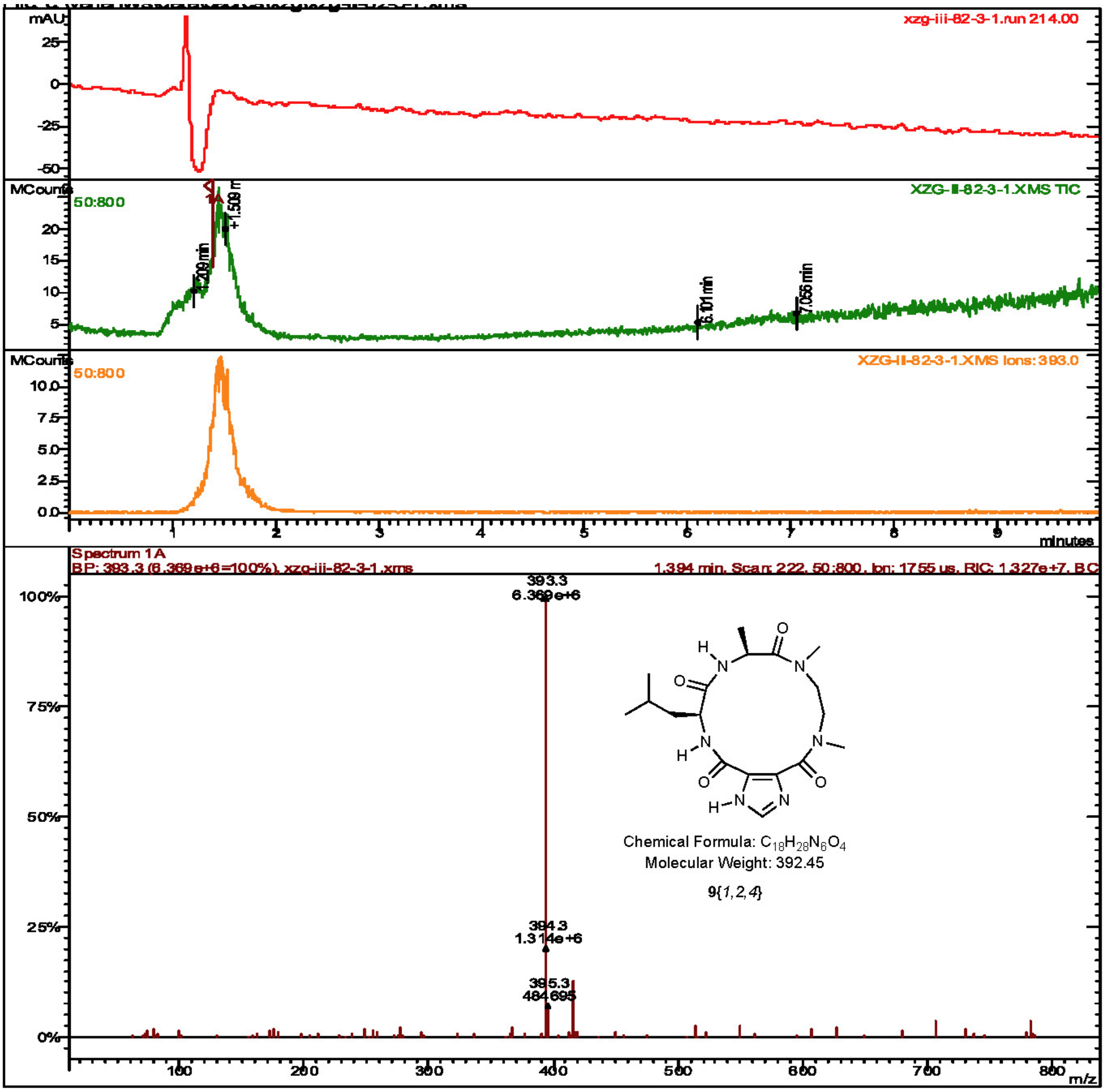

Figure S12. ^{1}H-NMR Spectra of **9**{*1,2,4*}.

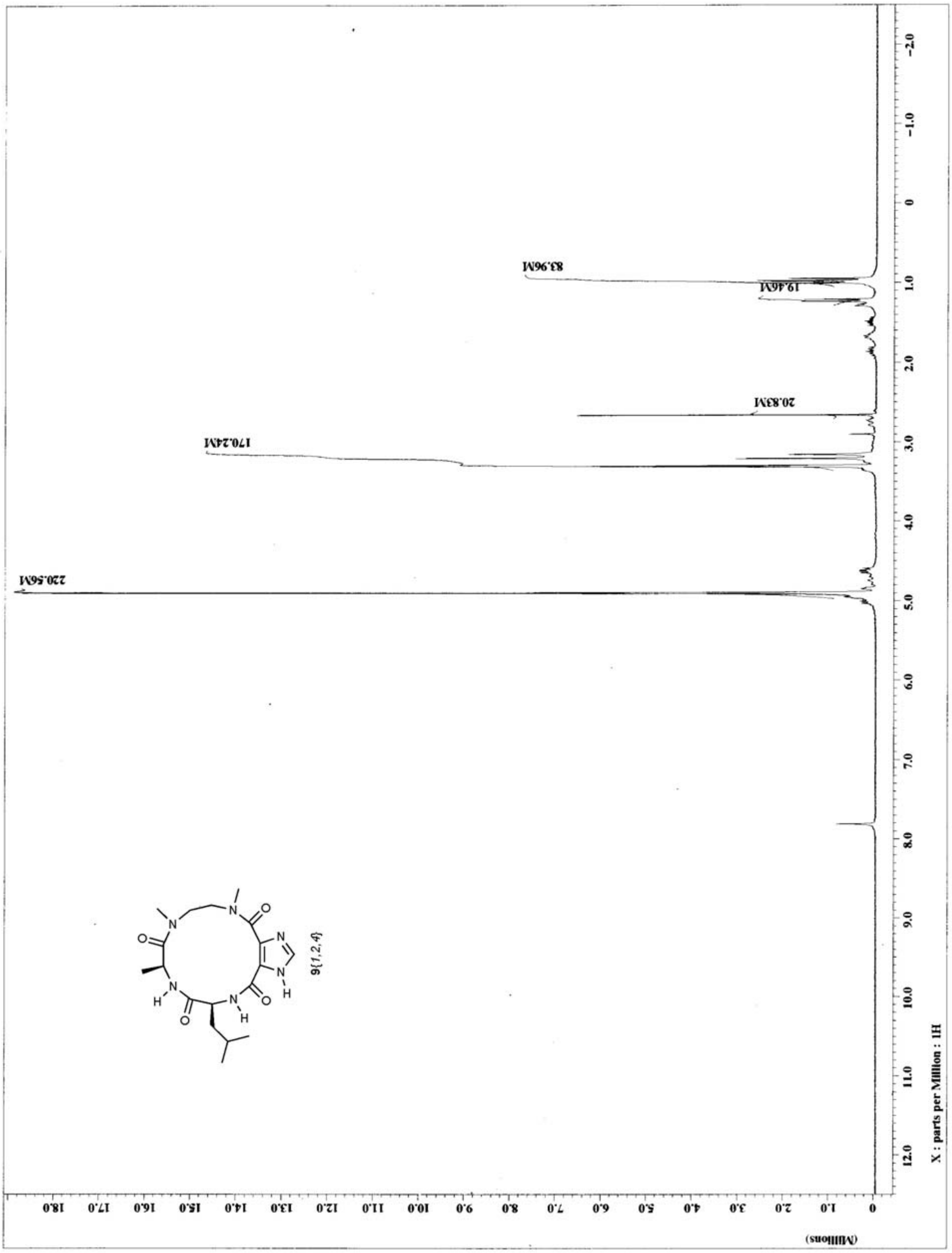

Figure S13. LC-MS of **9**{*1,3,2*}.

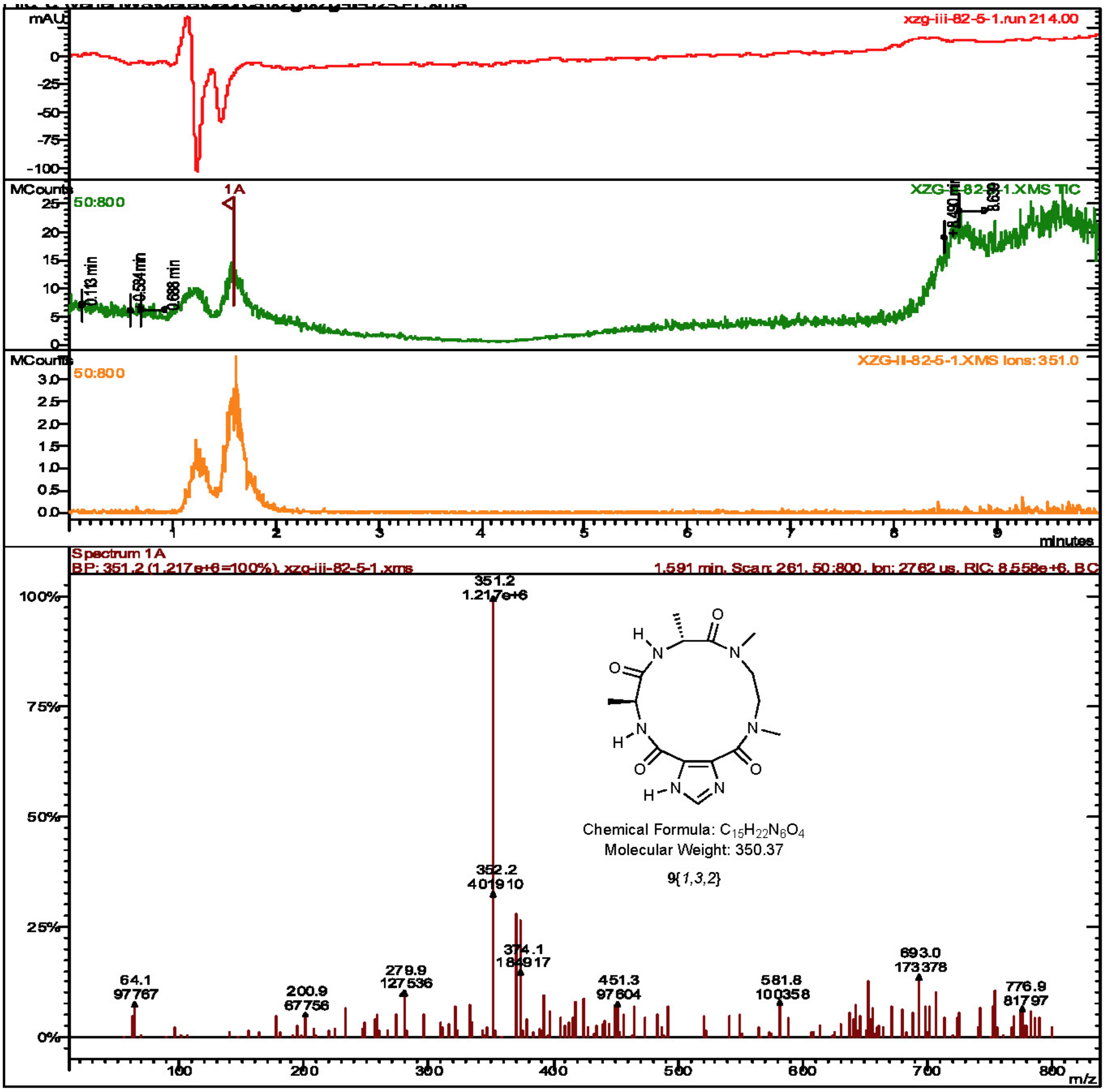

Figure S14. ^{1}H-NMR Spectra of **9**{*1,3,2*}.

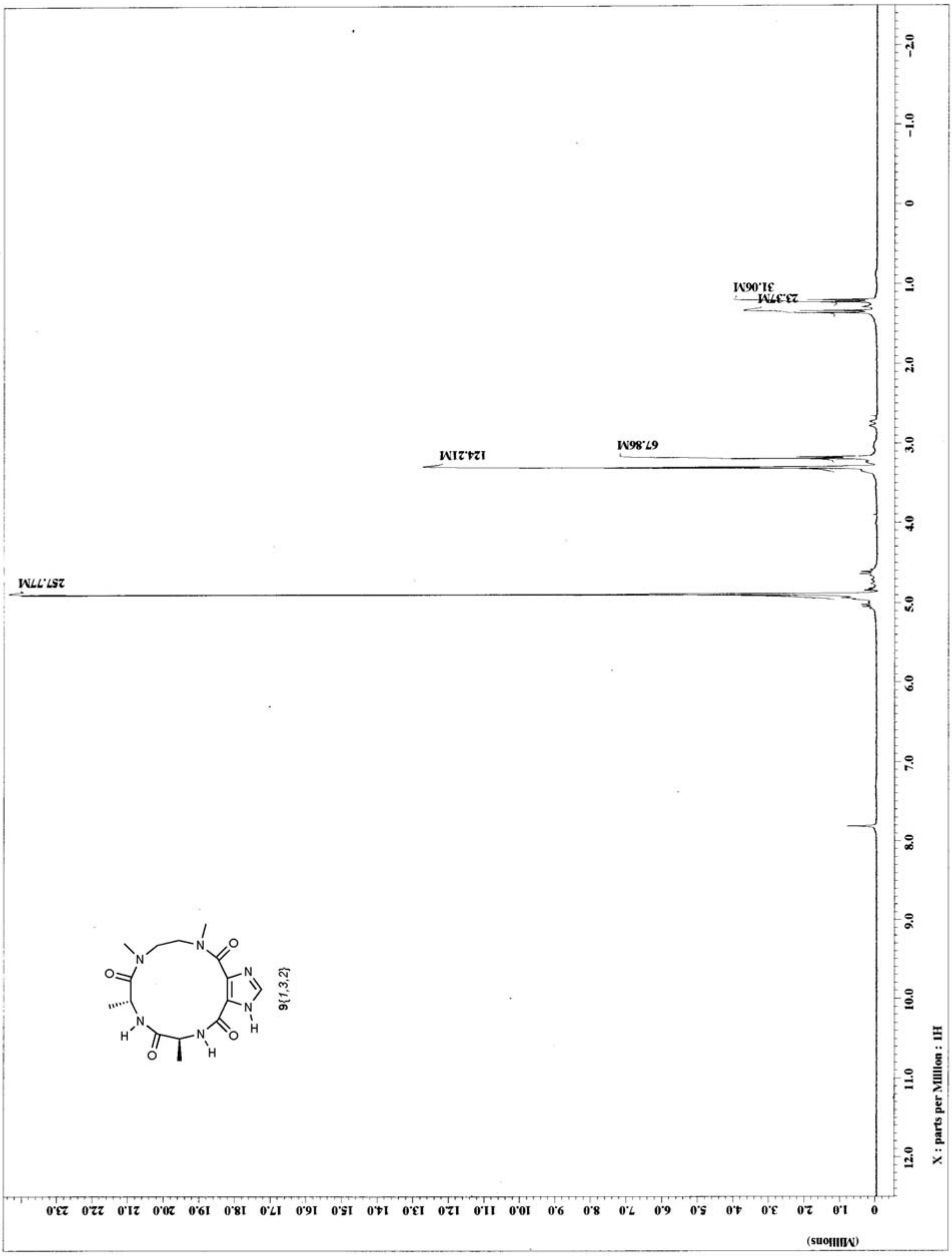

Figure S15. LC-MS of **9**{*1,3,4*}.

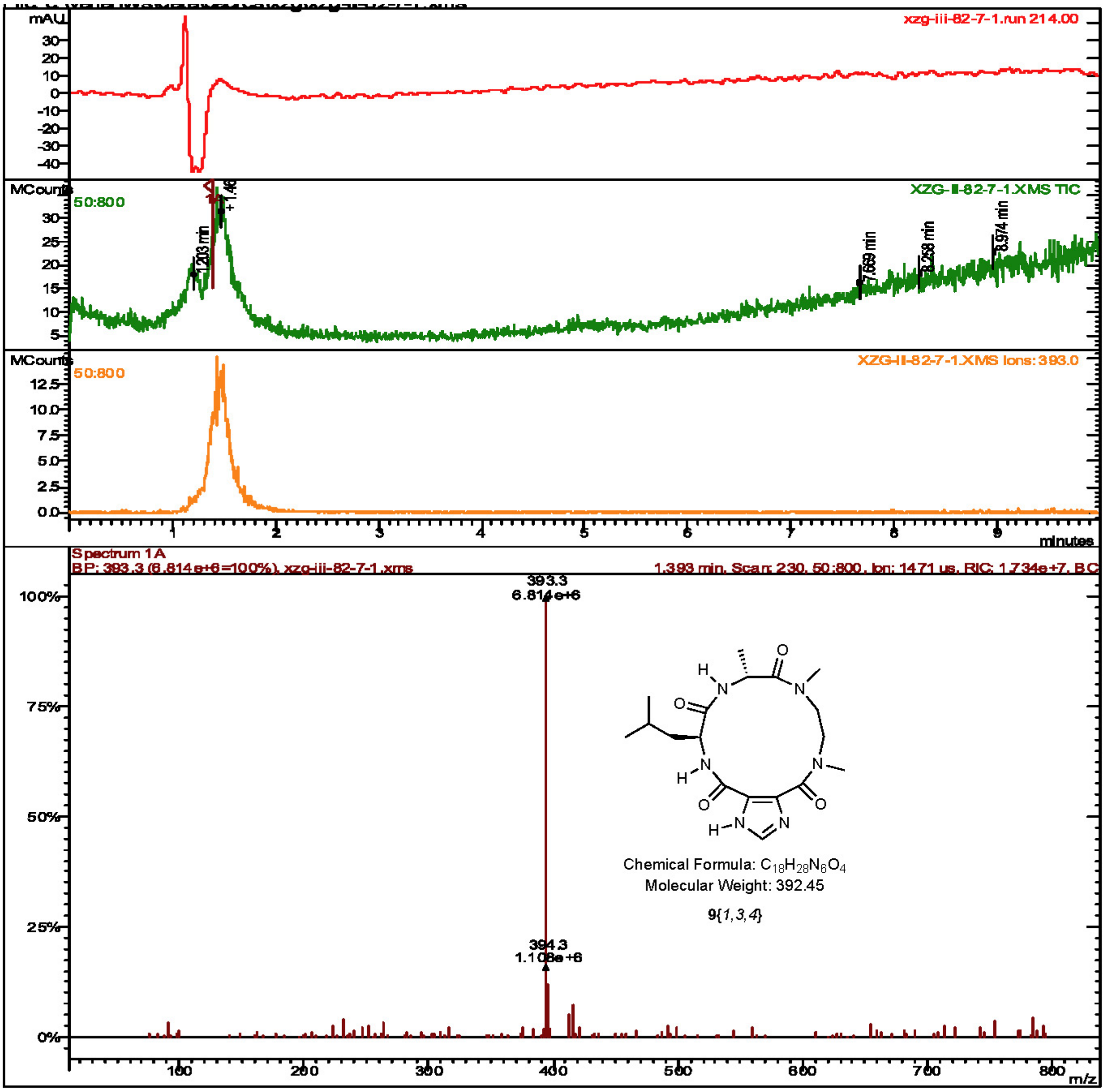

Figure S16. ^{1}H-NMR Spectra of **9**{*1,3,4*}.

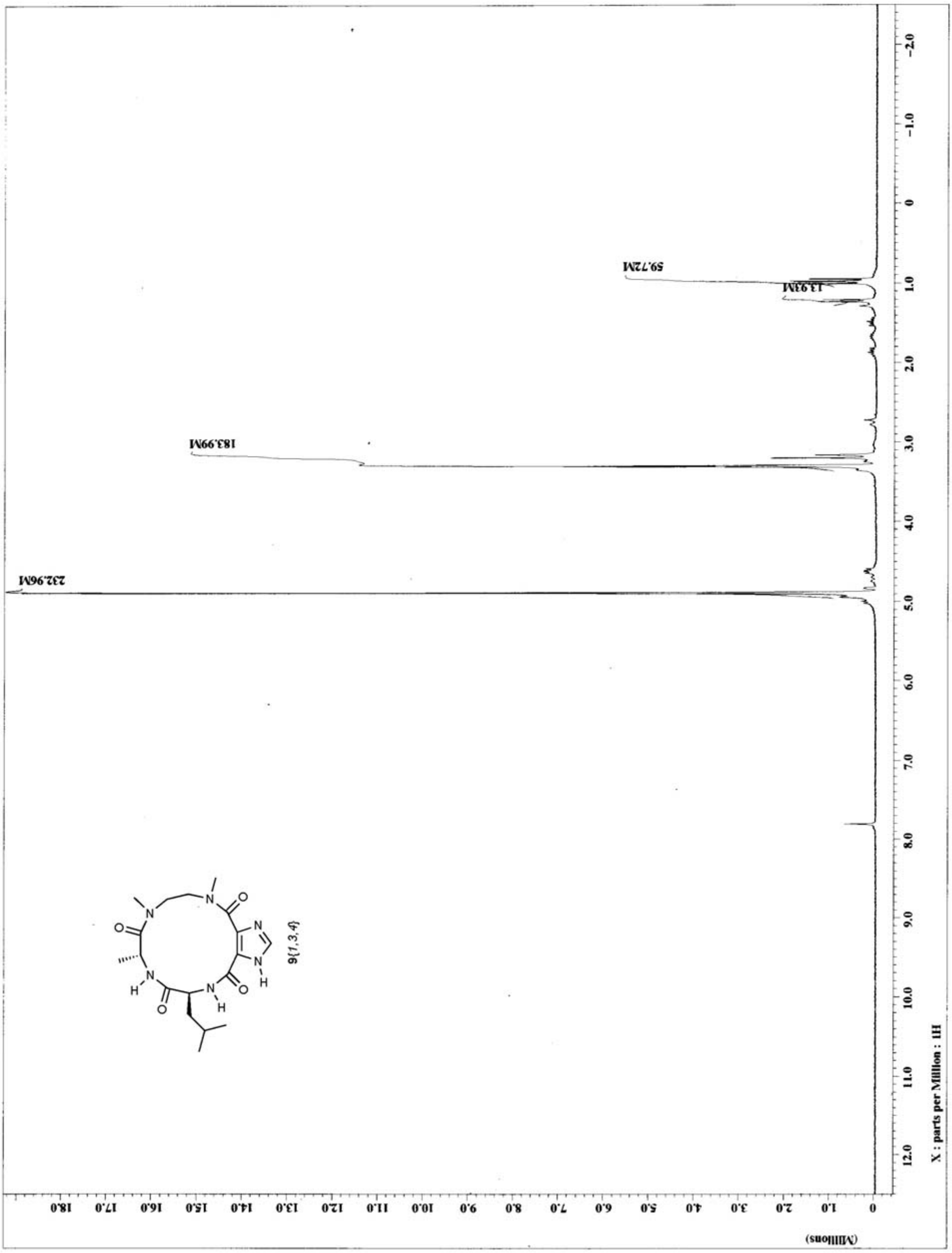

Figure S17. LC-MS of **9**{*1,4,2*}.

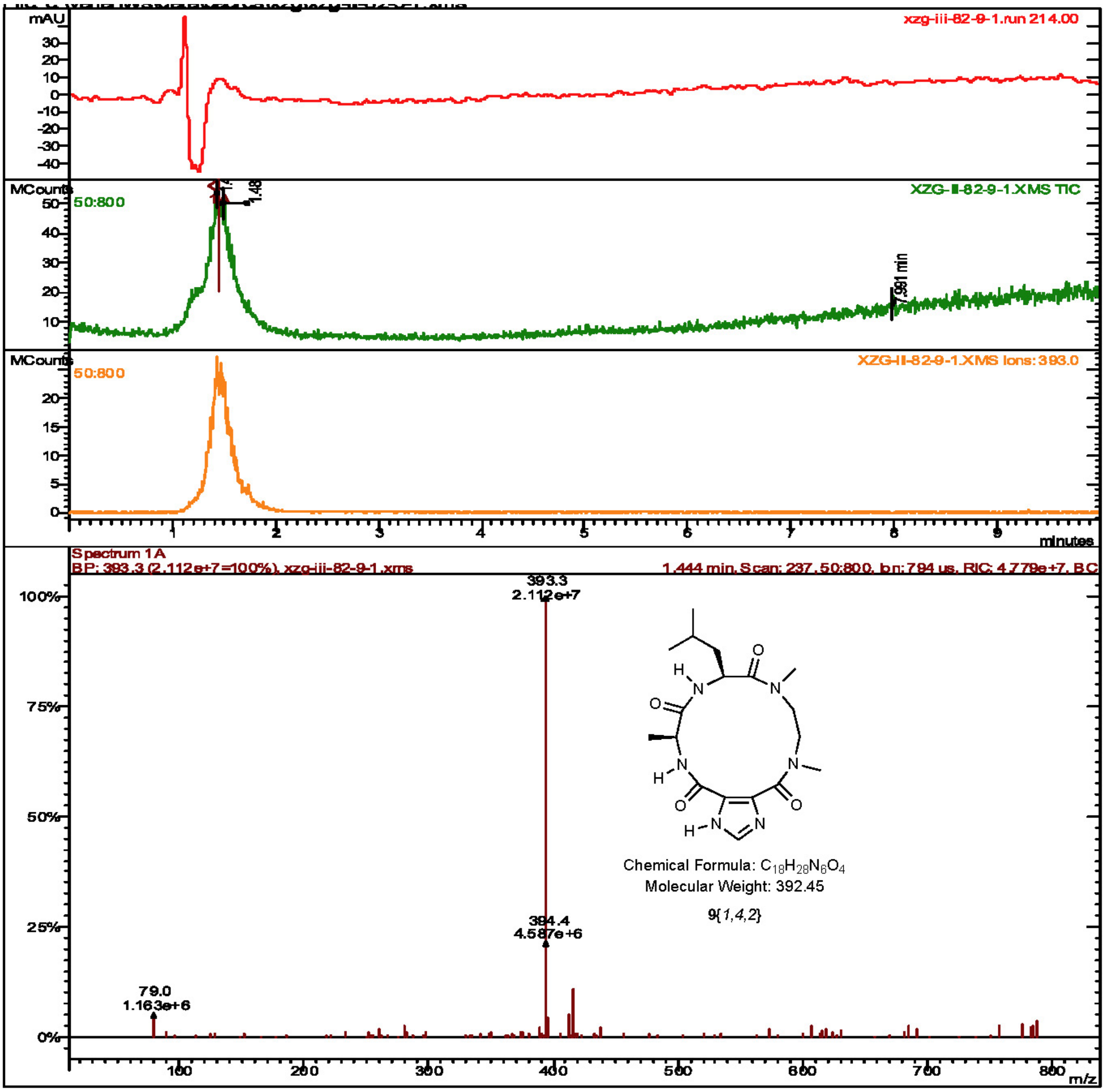

Figure S18. ^{1}H-NMR Spectra of **9**{*1,4,2*}.

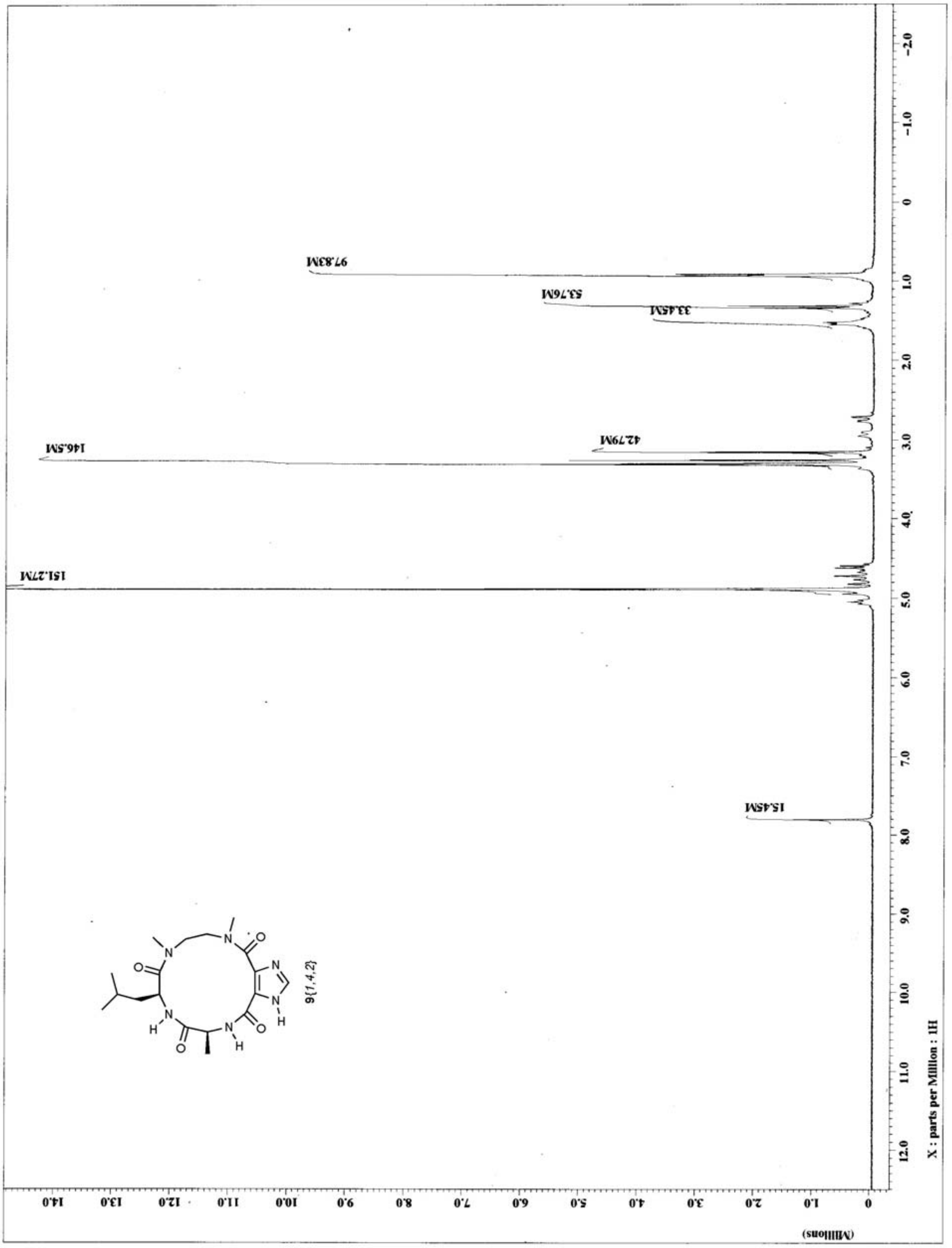

Figure S19. LC-MS of **9**{*1,4,3*}.

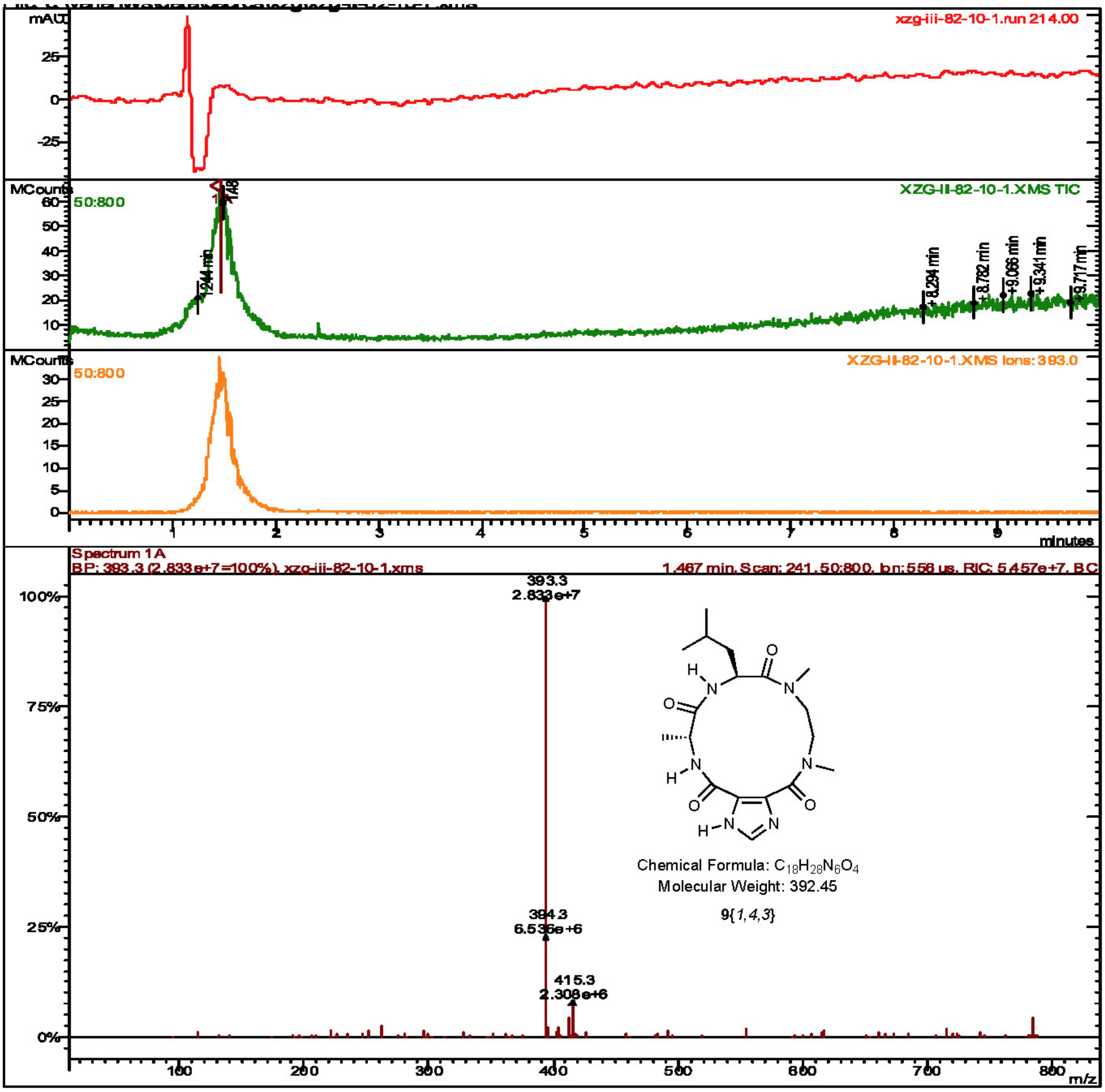

Figure S20. ^{1}H-NMR Spectra of **9**{*1,4,3*}.

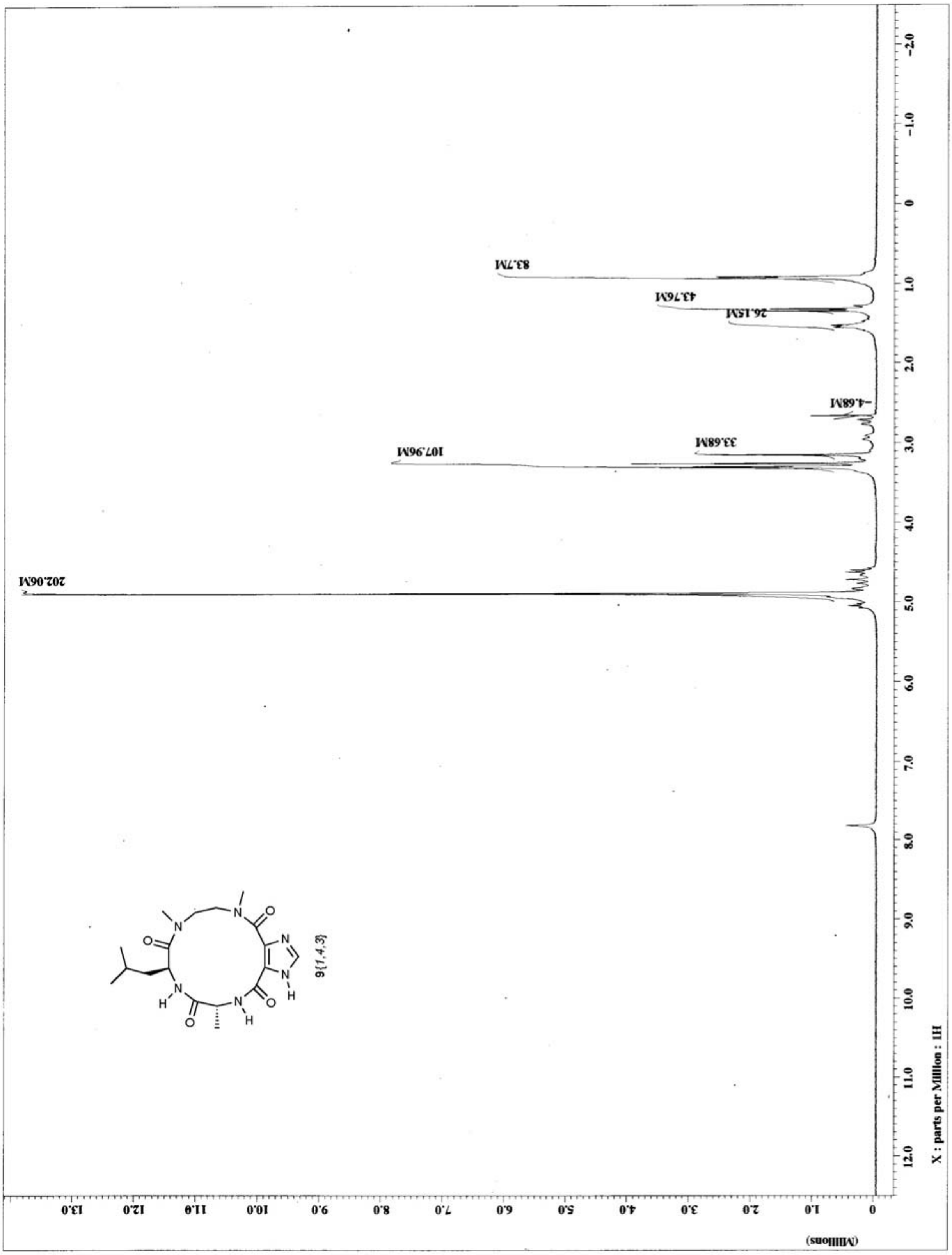

Figure S21. LC-MS of **9**{*2,1,2*}.

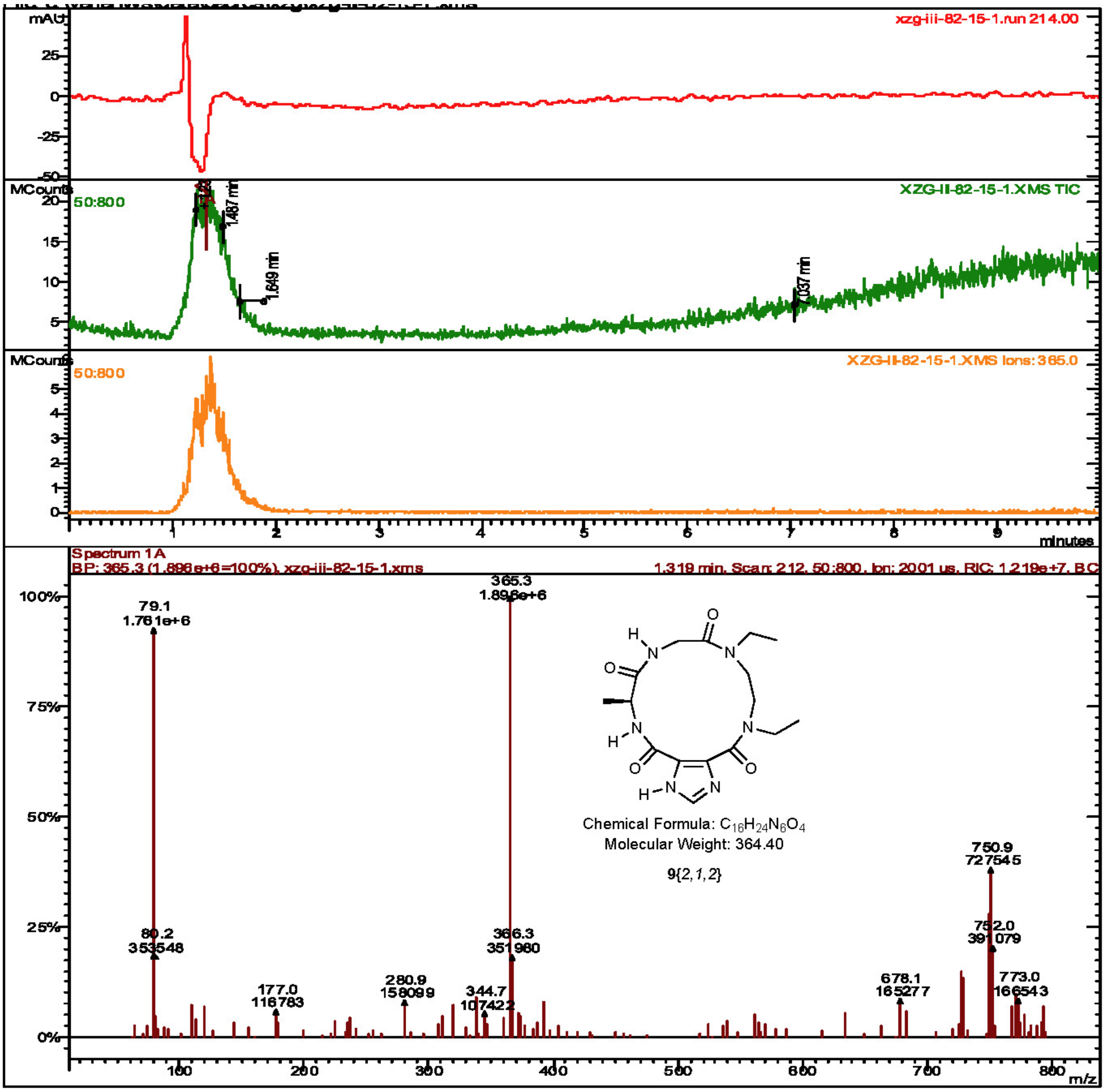

Figure S22. ^{1}H-NMR Spectra of **9**{*2,1,2*}.

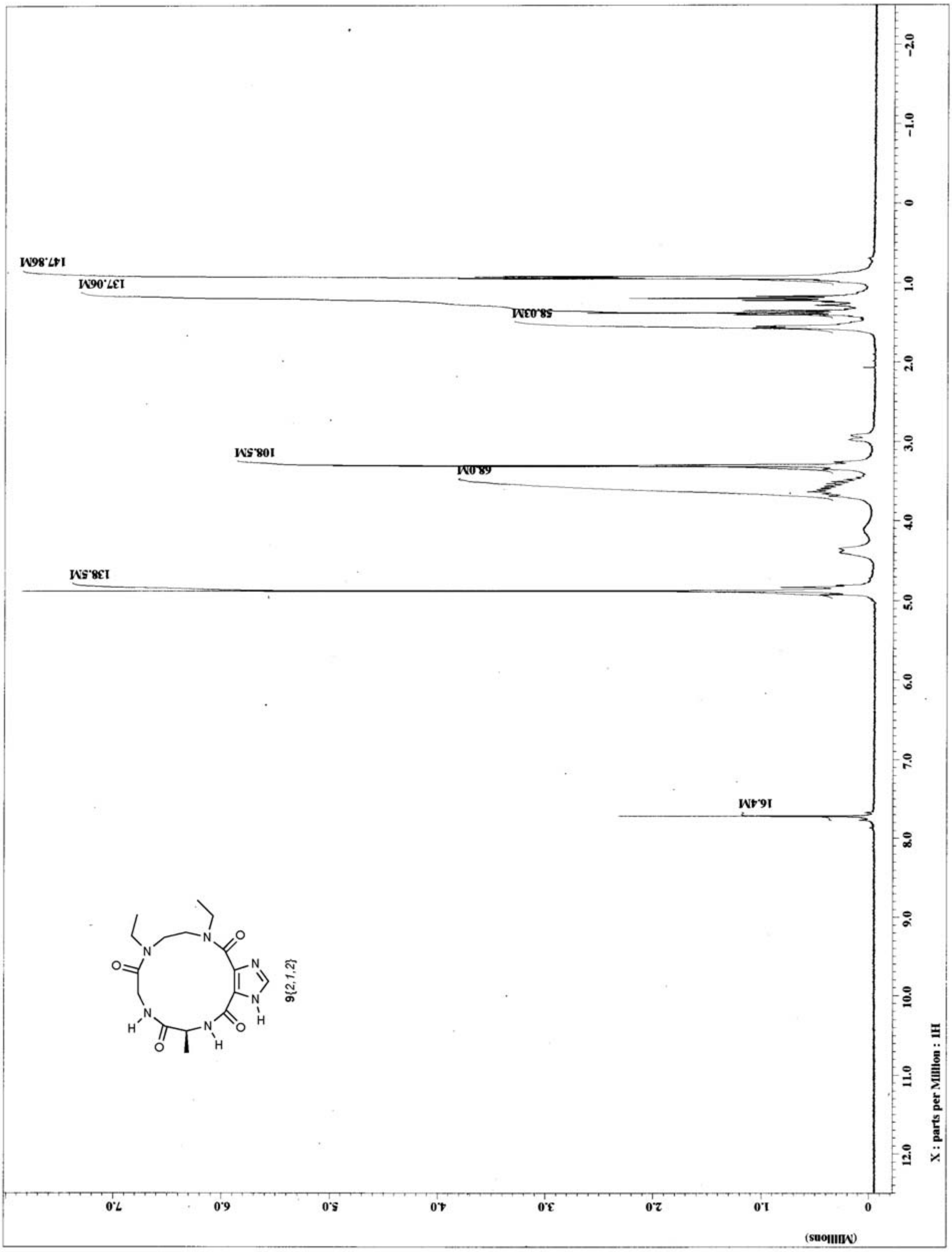

Figure S23. LC-MS of **9**{*2,1,3*}.

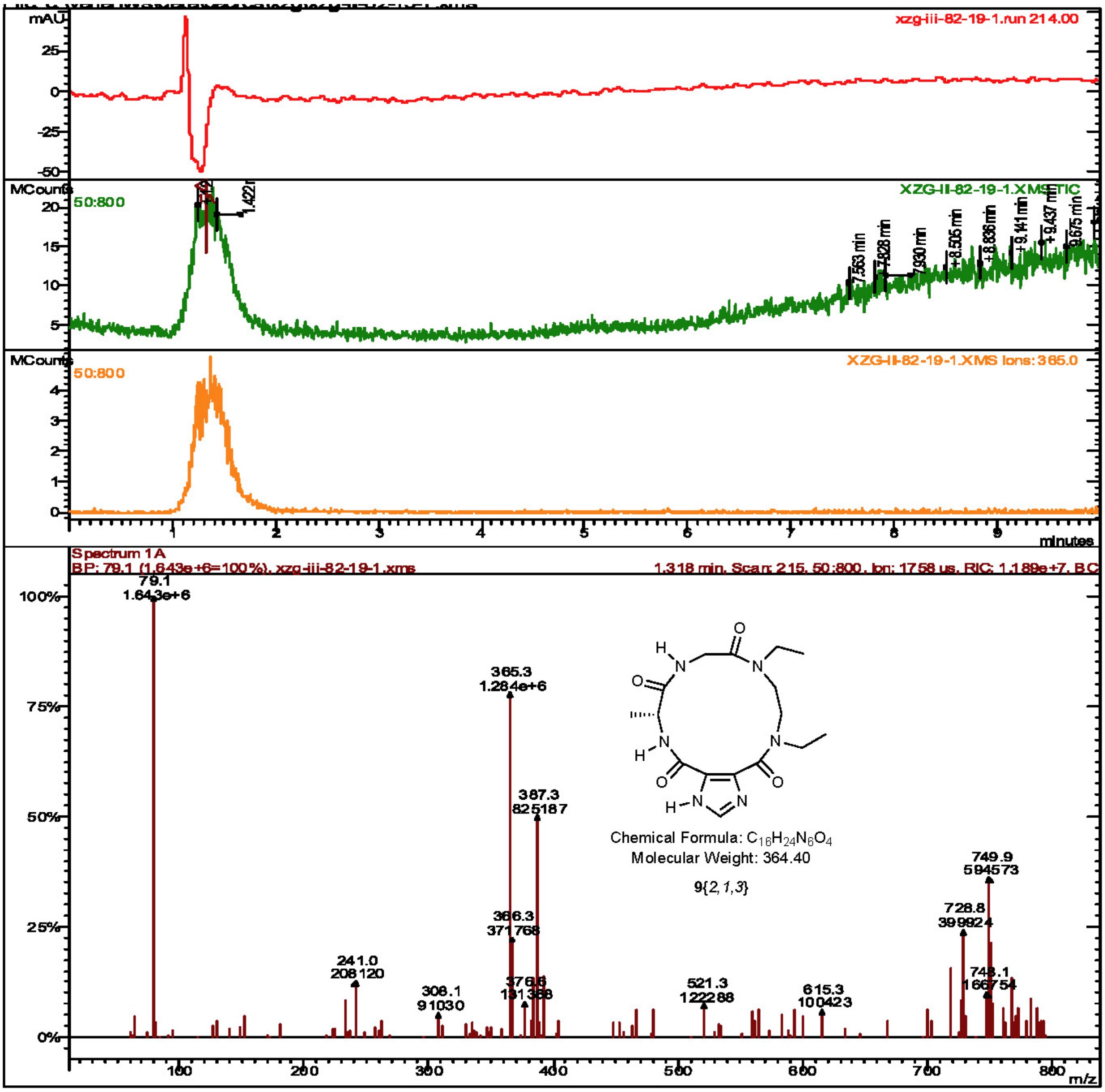

Figure S24. ^{1}H-NMR Spectra of **9**{*2,1,3*}.

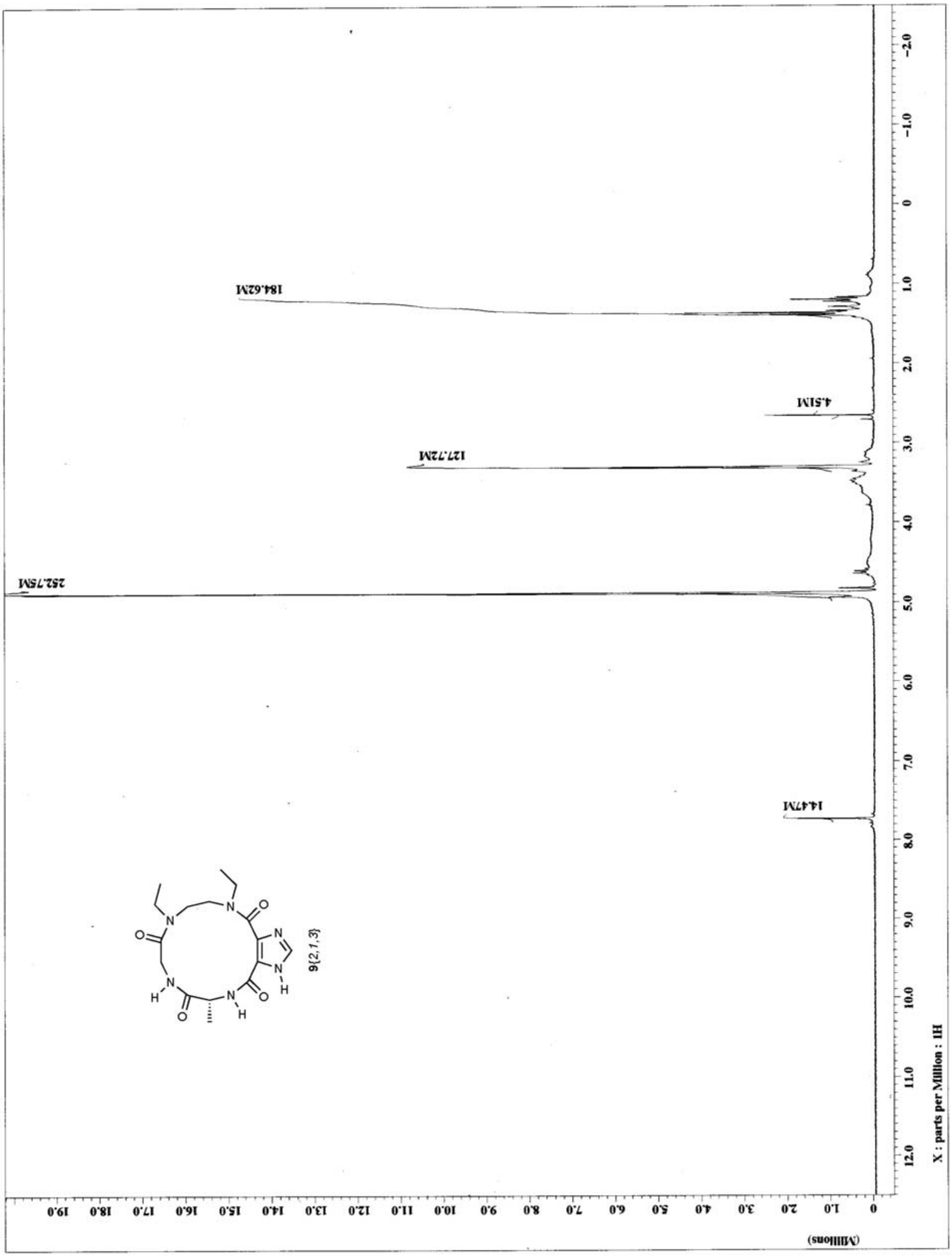

Figure S25. LC-MS of **9**{*2,2,2*}.

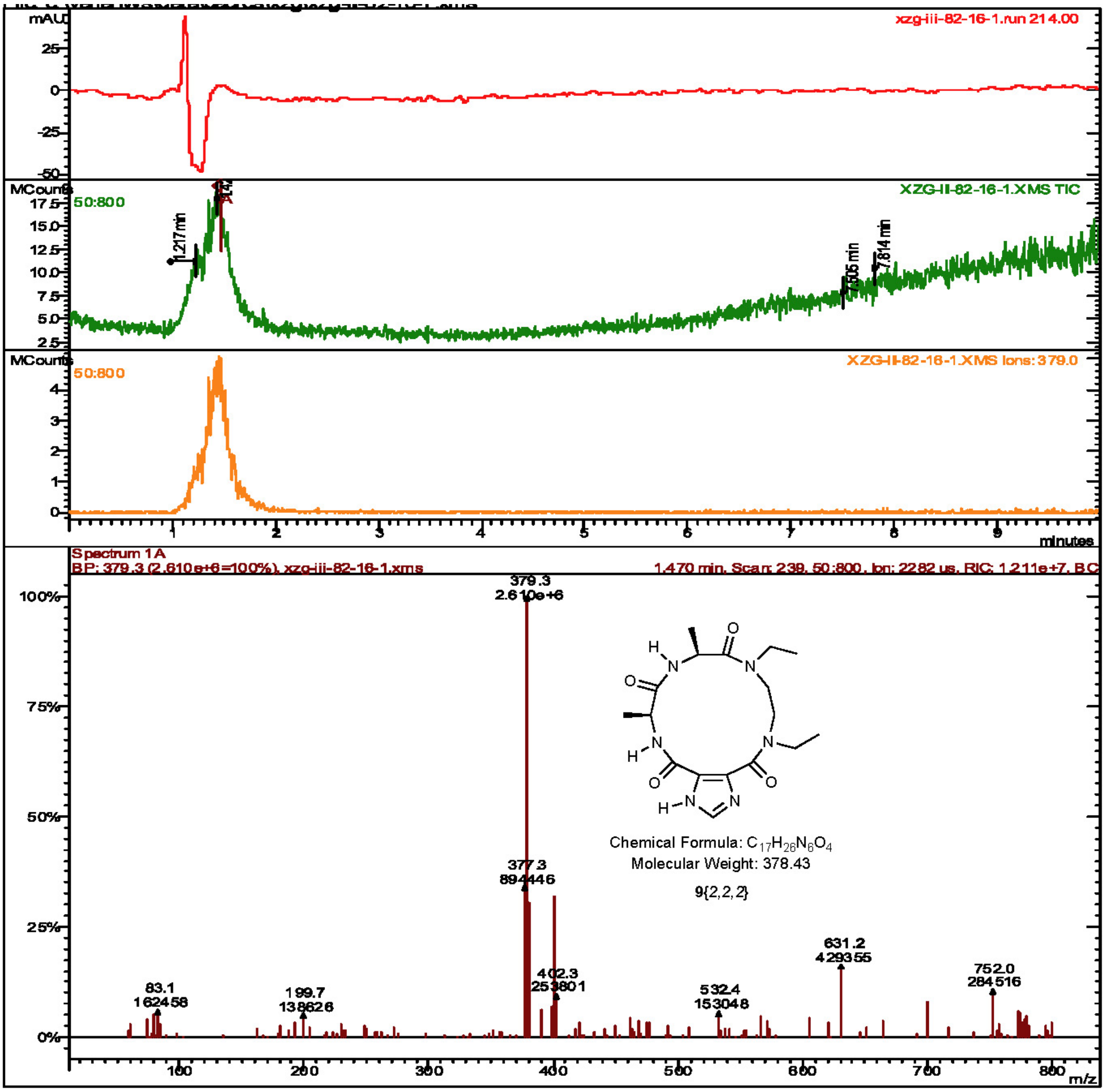

Figure S26. ^{1}H-NMR Spectra of **9**{*2,2,2*}.

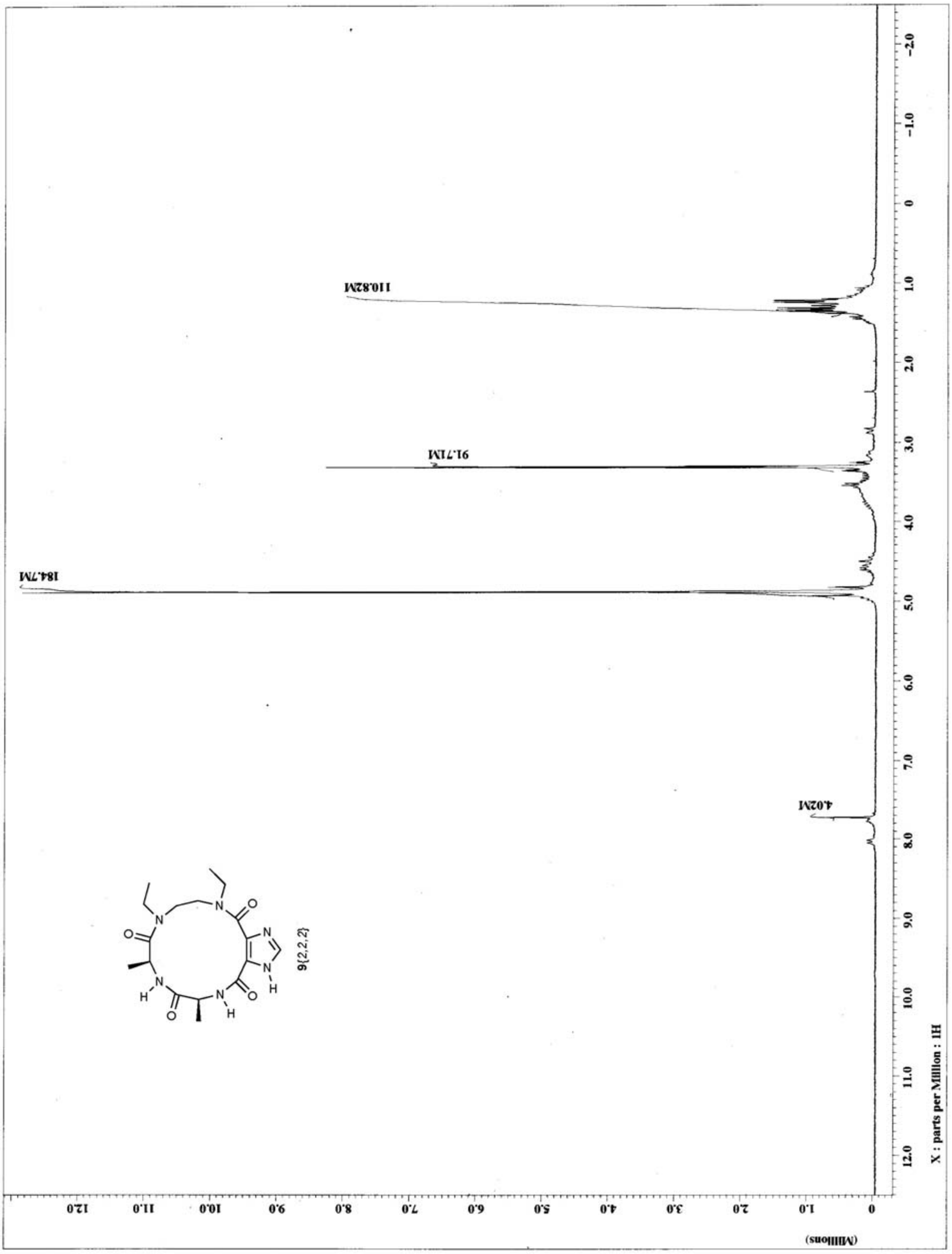

Figure S27. LC-MS of **9**{*2,2,3*}.

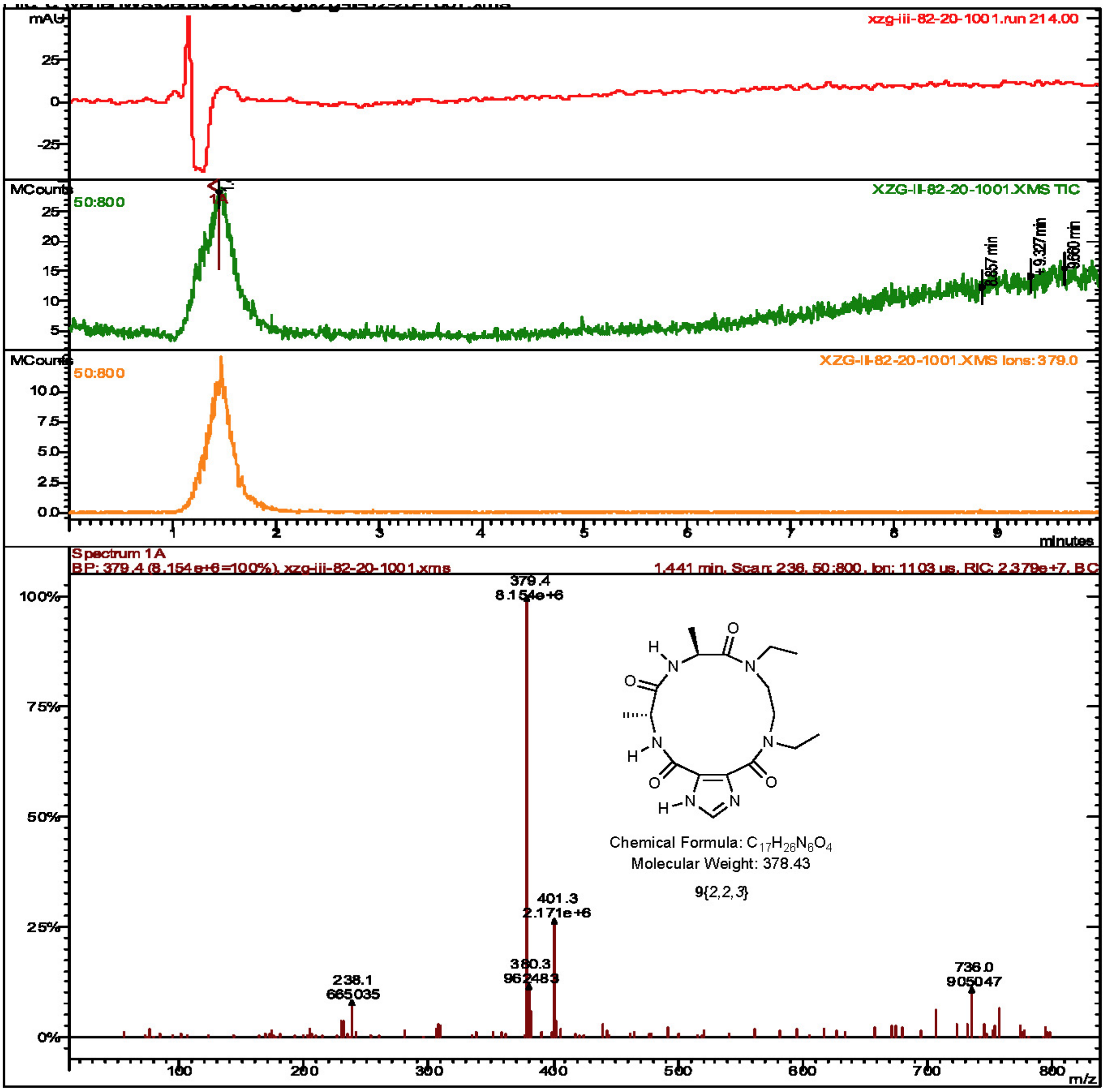

Figure S28. ^{1}H-NMR Spectra of **9**{*2,2,3*}.

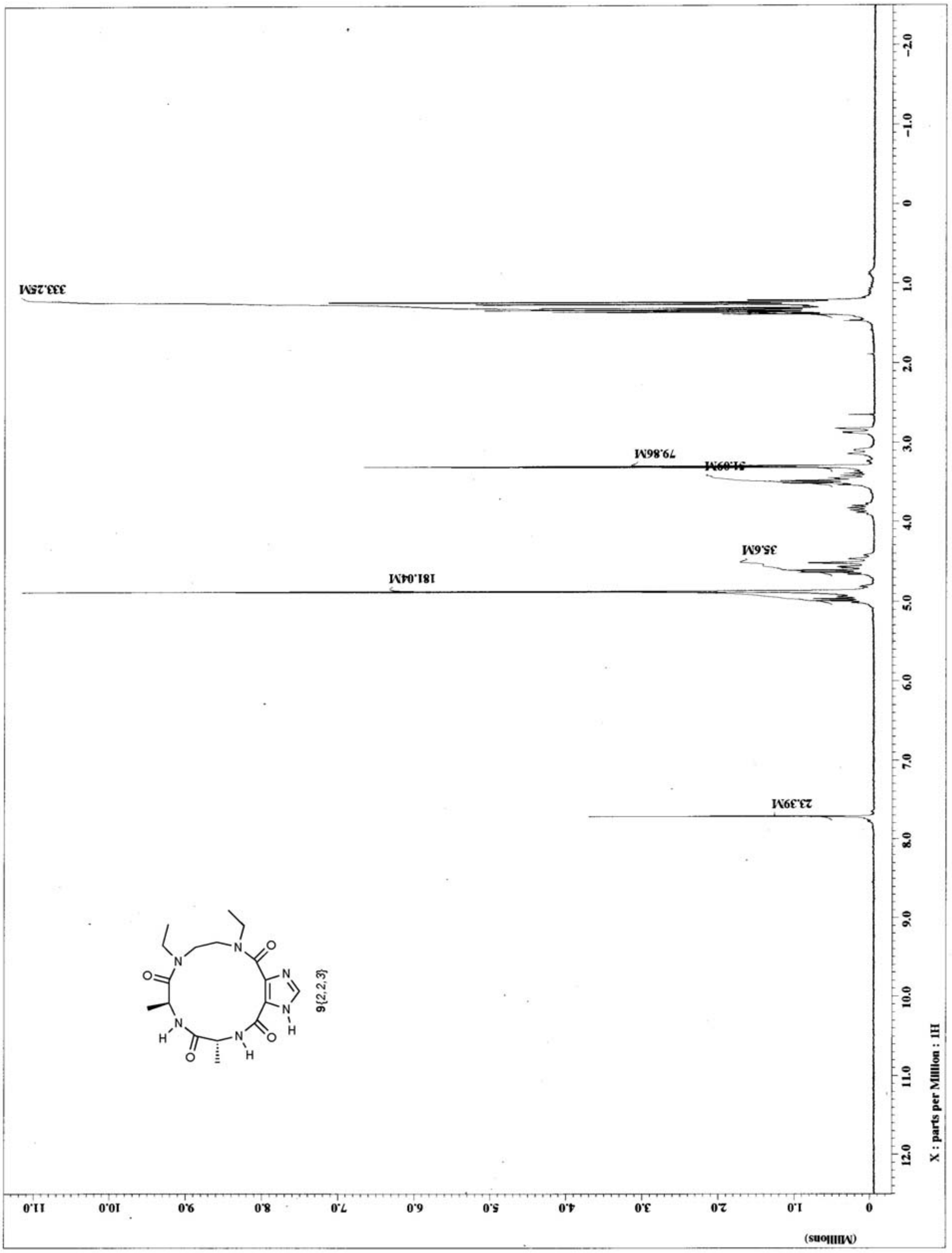

Figure S29. LC-MS of **9**{*2,3,1*}.

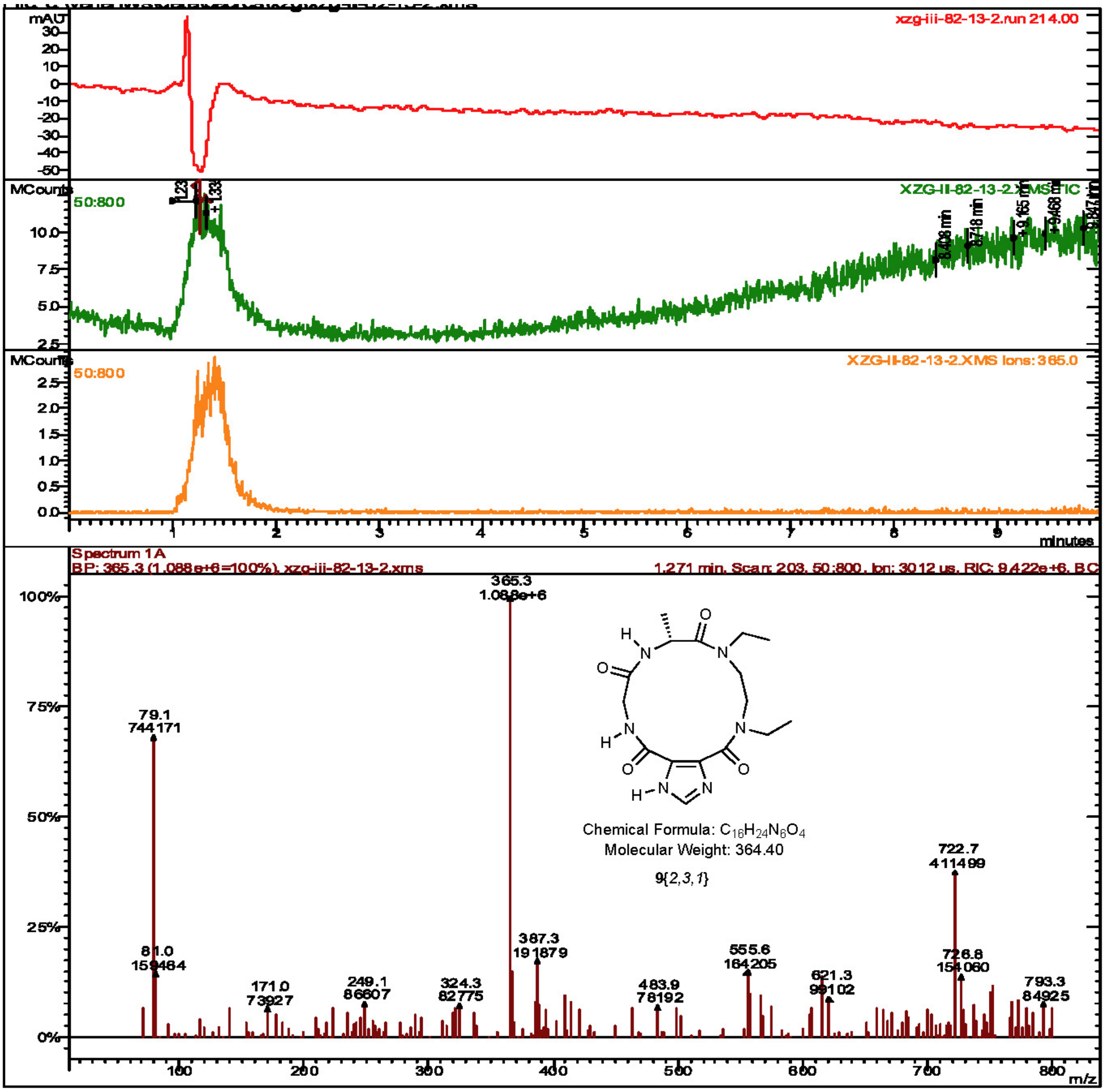

Figure S30. ^{1}H-NMR Spectra of **9**{*2,3,1*}.

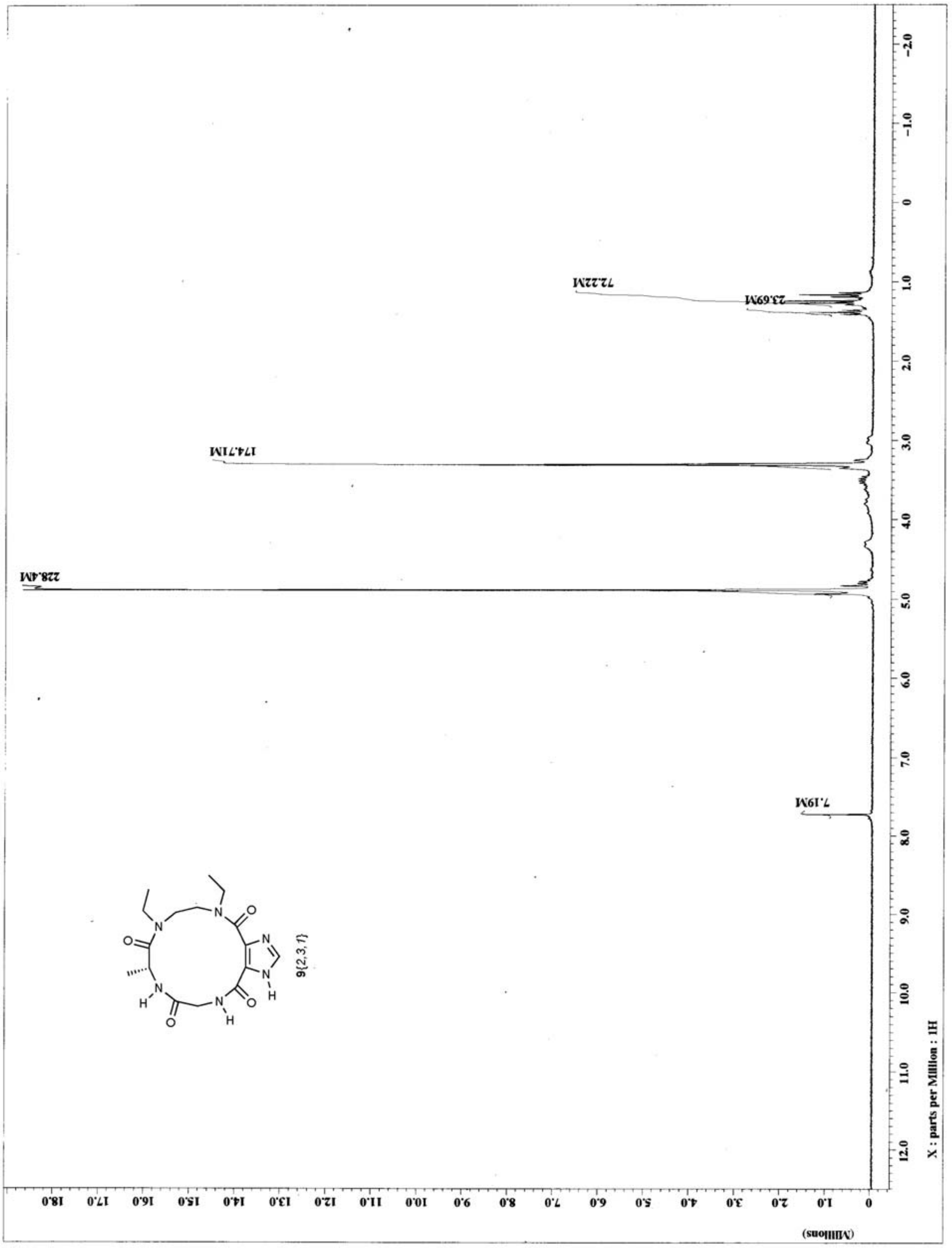

Figure S31. LC-MS of **9**{*2,3,2*}.

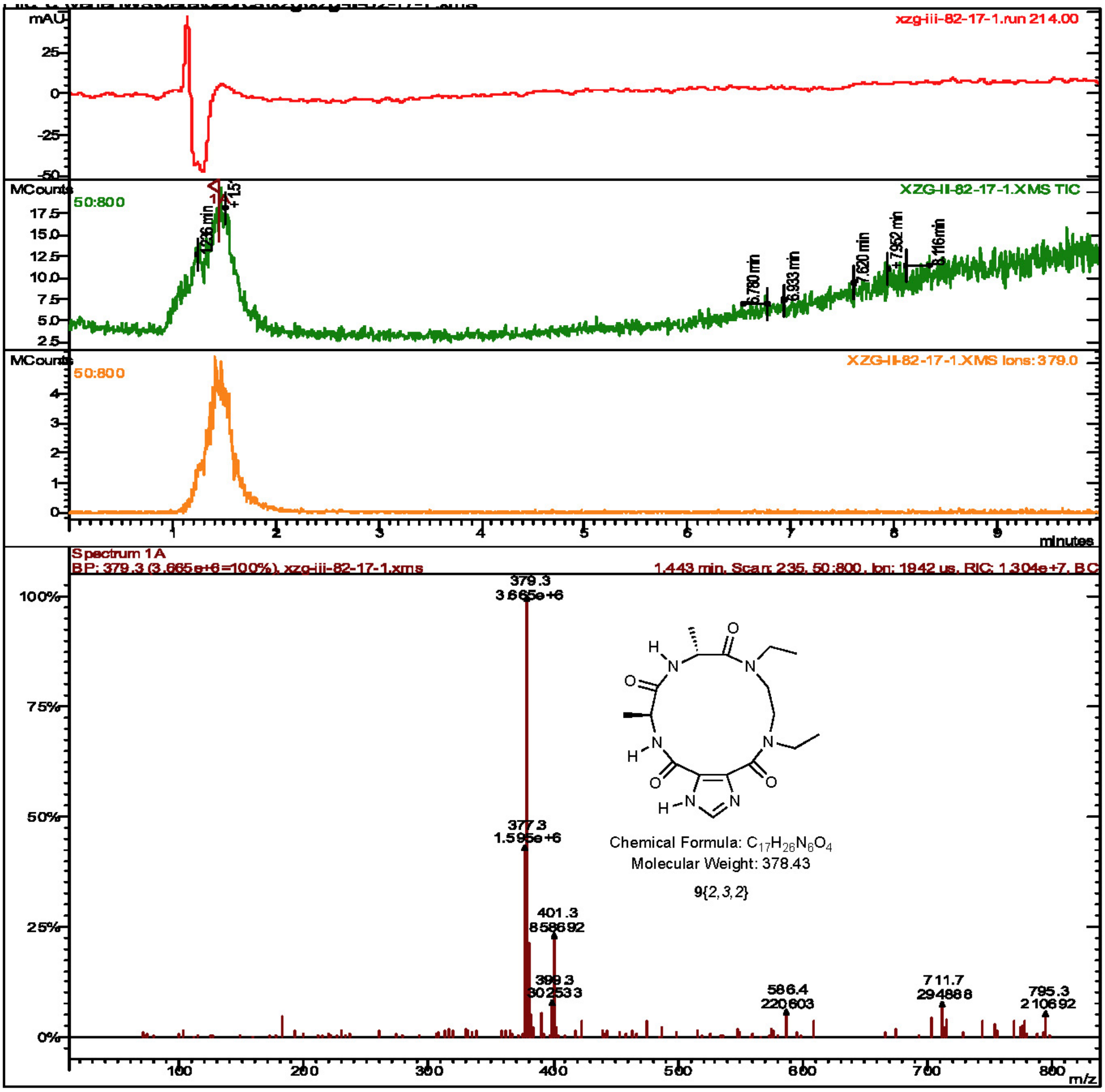

Figure S32. ^{1}H-NMR Spectra of **9**{*2,3,2*}.

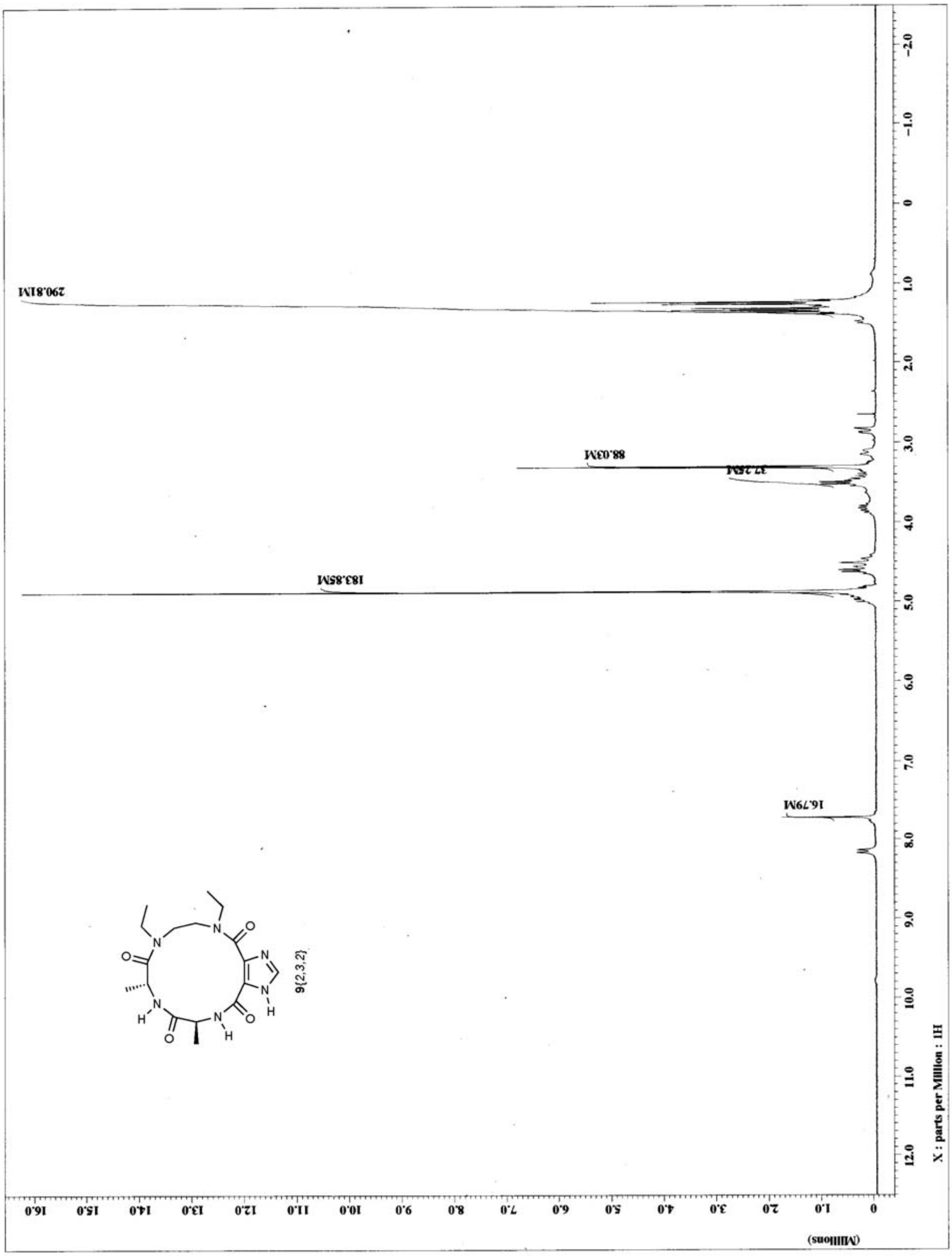

Figure S33. LC-MS of **9**{*2,3,3*}.

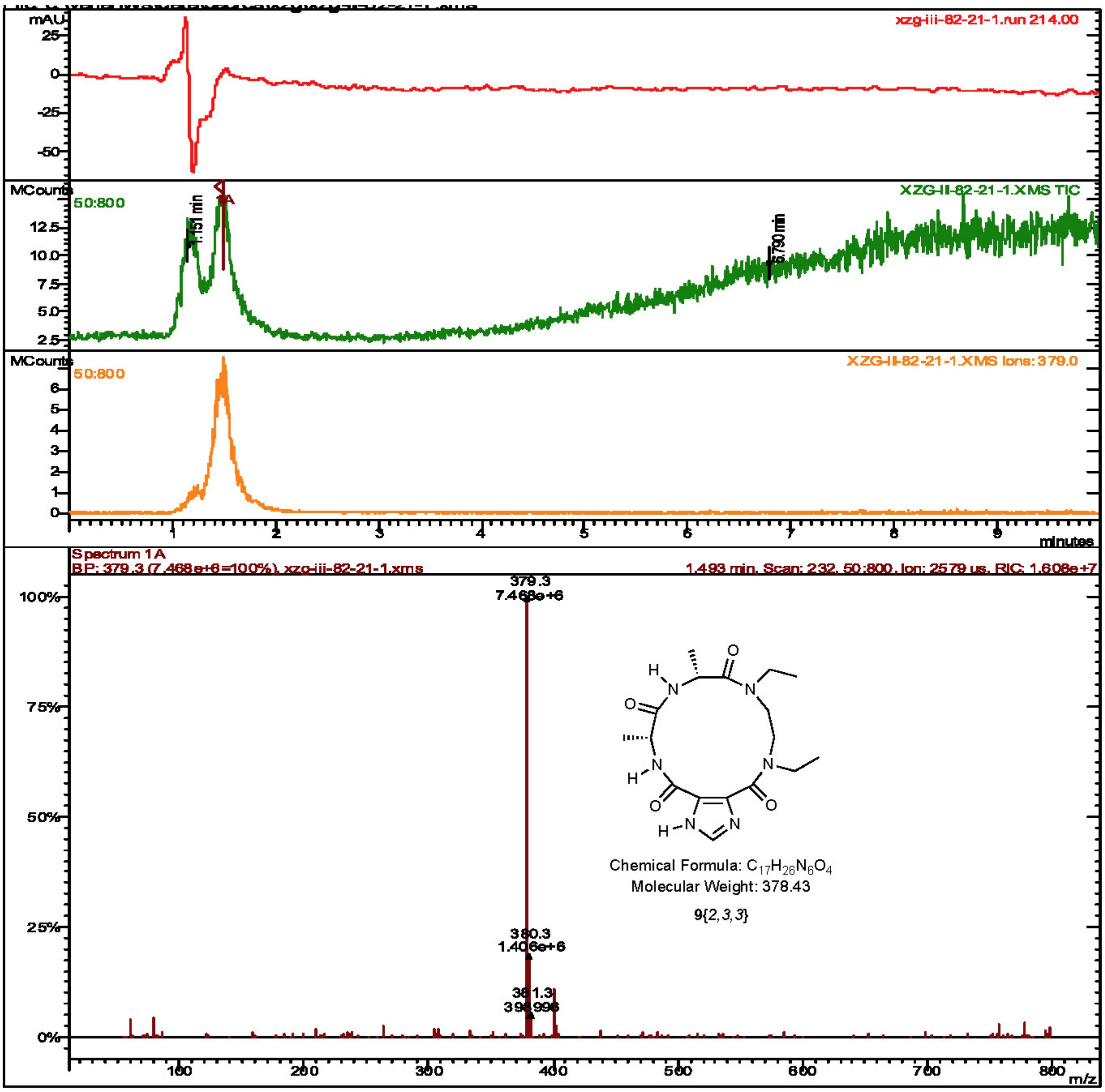

Figure S34. ^{1}H-NMR Spectra of **9**{*2,3,3*}.

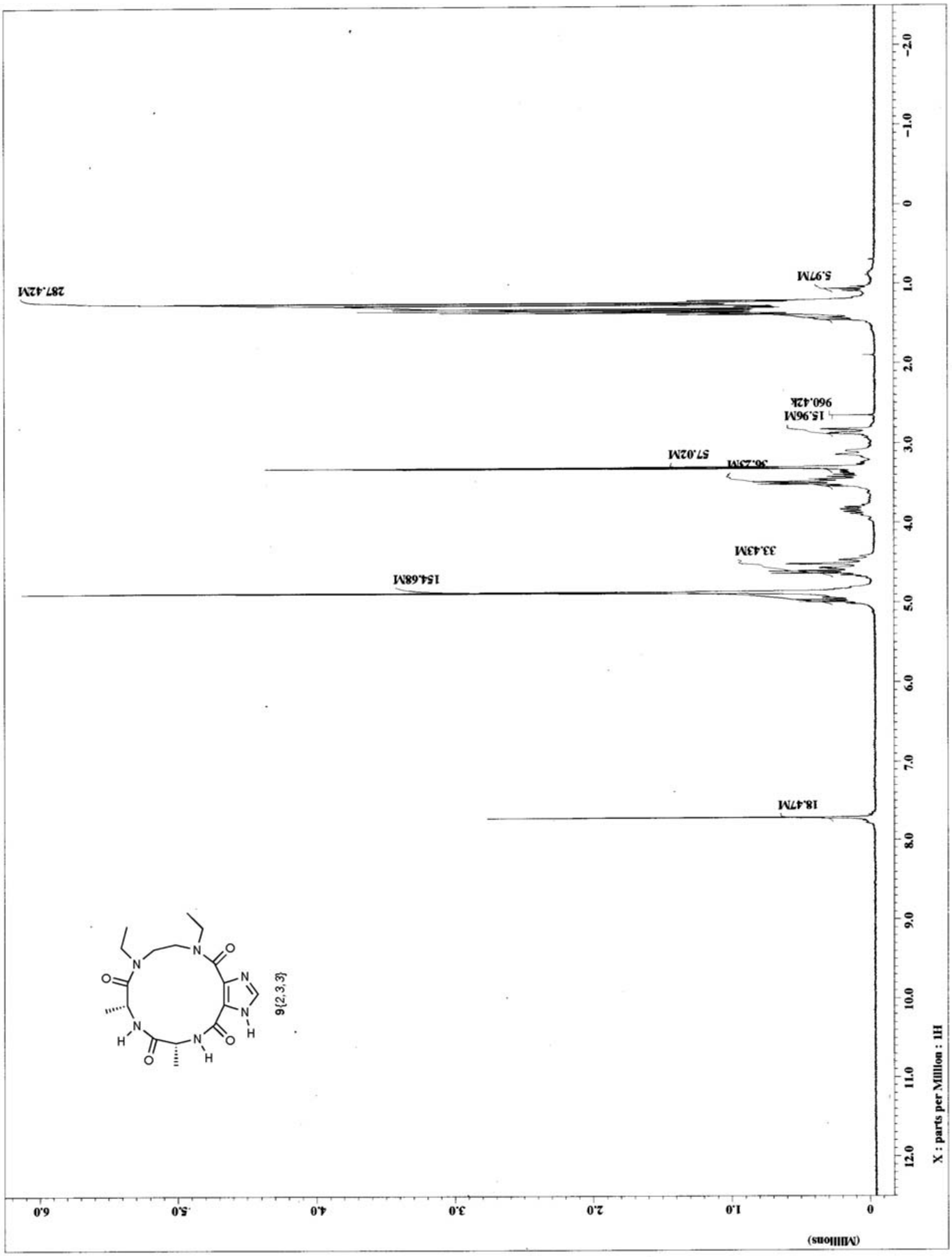

Figure S35. LC-MS of **9**{*2,4,1*}.

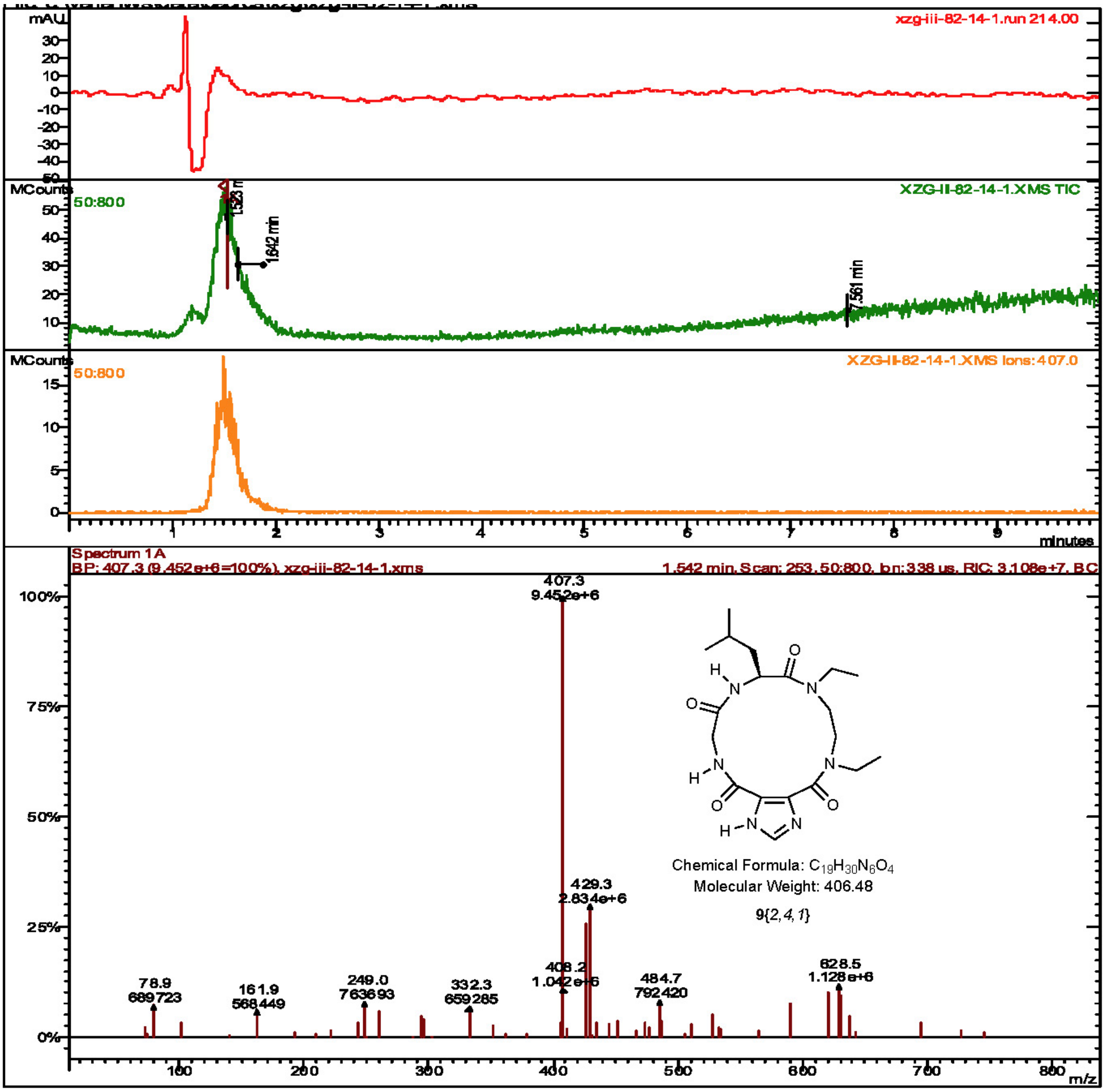

Figure S36. ^{1}H-NMR Spectra of **9**{*2,4,1*}.

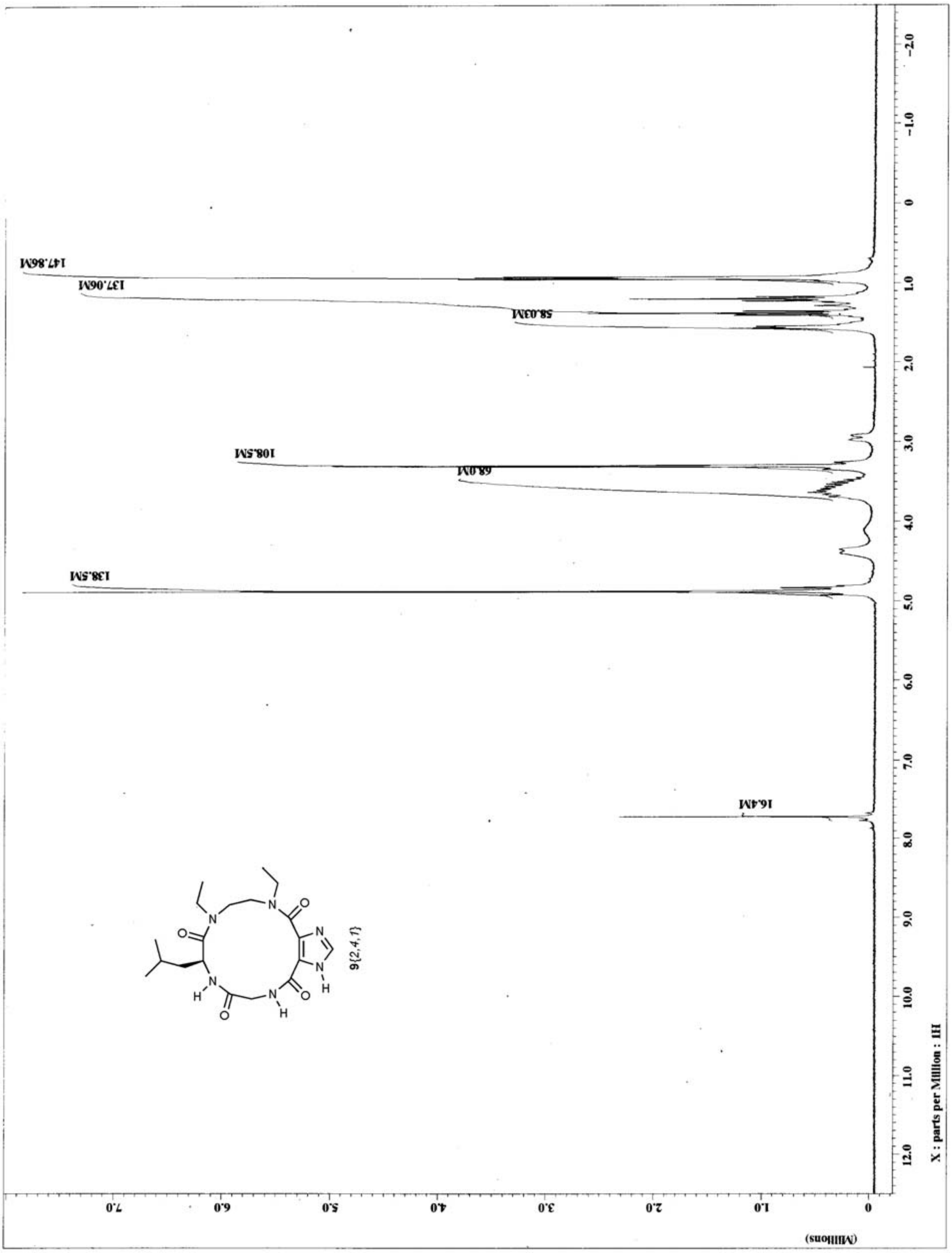

Figure S37. LC-MS of **9**{*2,4,2*}.

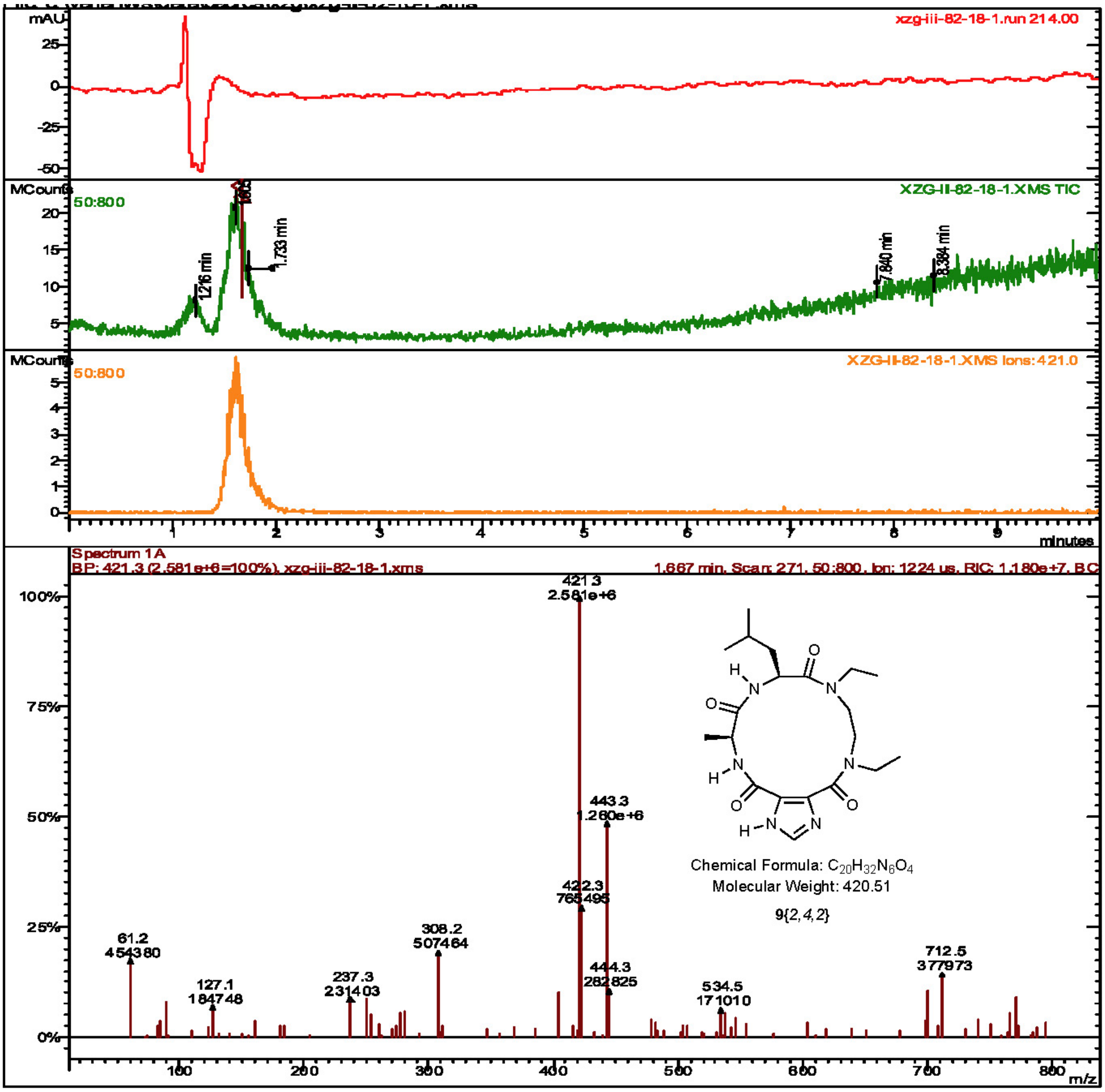

Figure S38. ^{1}H-NMR Spectra of **9**{*2,4,2*}.

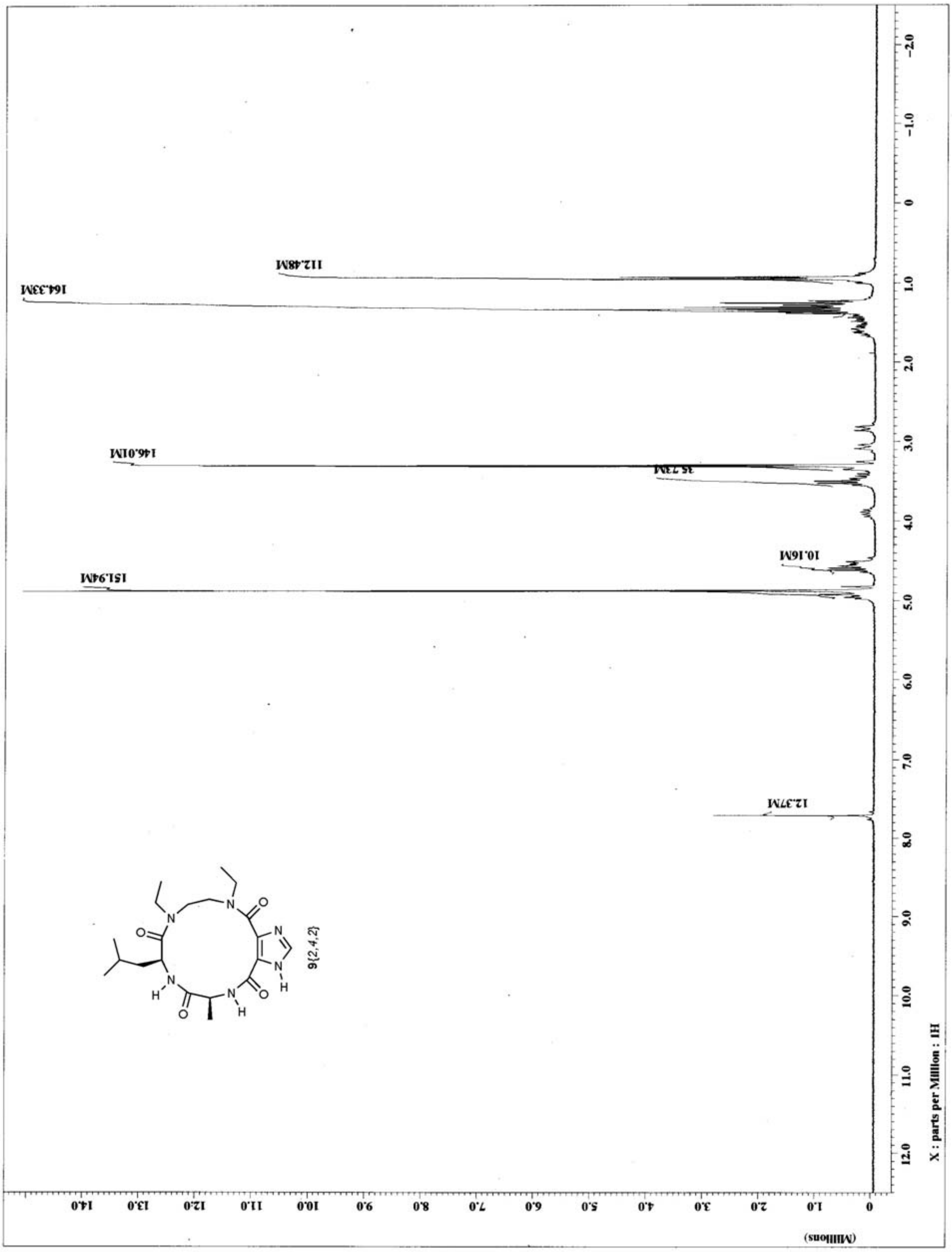

Supplement: Supplementary file 1 [file molecules-17-05346-s001.pdf]
